# Assessing Venous Congestion in Acute and Chronic Heart Failure: A Review of Splanchnic, Cardiac and Pulmonary Ultrasound: Part 1: Conventional B-Mode, Colordoppler, and Vexus Protocol

**DOI:** 10.3390/jcm14228147

**Published:** 2025-11-17

**Authors:** Francesco Giangregorio, Ester Centenara, Samanta Mazzocchi, Luigi Gerra, Francesco Tursi, Davide Imberti, Daniela Aschieri

**Affiliations:** 1 Department of Internal Medicine, Castel San Giovanni Hospital, Vle II Giugno, 1, 29015 Castel San Giovanni, Italy; e.centenara@ausl.pc.it (E.C.); s.mazzocchi@ausl.pc.it (S.M.); 2 Department of Cardiology, Piacenza Hospital, Via Taverna 49, 29121 Piacenza, Italy; l.gerra@ausl.pc.it (L.G.); d.aschieri@ausl.pc.it (D.A.); 3 Cardiac and Pneumological Rehabilitation Medicine, Codogno Hospital, 26845 Codogno, Italy; francesco.tursi@asst-lodi.it; 4 Department of Internal Medicine, Piacenza Hospital, Via Taverna 49, 29121 Piacenza, Italy; d.imberti@ausl.pc.it

**Keywords:** heart failure, splanchnic circulation, venous congestion, ultrasound, point-of-care ultrasound (POCUS), Doppler imaging, hepatic veins, portal vein, VExUS, congestion assessment, lung congestion, cardiac congestion

## Abstract

**Background/Objectives**: Heart failure (HF) causes systemic and regional haemodynamic alterations that extend beyond the heart, profoundly affecting splanchnic circulation. Venous congestion is a hallmark of heart failure (HF) and a major determinant of clinical deterioration and multiorgan dysfunction. The splanchnic venous system—comprising the portal, hepatic, and renal veins—acts as a key reservoir for intravascular volume redistribution. Conventional ultrasound (US), using grayscale and Doppler imaging, offers a direct, non-invasive approach to visualize these haemodynamic changes. This review, Part 1 of a two-part series, summarizes the current evidence and clinical applications of conventional US for assessing splanchnic, cardiac and pulmonary vascular alterations in patients with HF. **Methods**: A systematic review was performed in PubMed, Embase, and the Cochrane Library up to current date, following PRISMA 2020 guidelines. Eligible studies included adult human investigations evaluating splanchnic vascular changes in HF using B-mode, color Doppler, or pulsed Doppler ultrasonography. Exclusion criteria were pediatric, animal, or non-English studies and non-standard imaging methods. Data on ultrasonographic parameters, haemodynamic correlations, and prognostic value were extracted and qualitatively synthesized; **Results**: A total of 148 eligible studies (*n* ≈ 7000 patients) demonstrated consistent associations between HF severity and alterations in splanchnic, cardiac and pulmonary flow. Findings included increased bowel wall thickness, portal vein dilation with elevated pulsatility, and monophasic or reversed hepatic vein waveforms, all correlating with higher right atrial pressure and adverse clinical outcomes. The integration of these parameters into the Venous Excess Ultrasound (VExUS) framework enhanced detection of systemic venous congestion, in addition to the study of the cardiac and pulmonary circulation. **Conclusions**: Conventional ultrasound assessment of splanchnic vasculature provides valuable, reproducible insight into systemic congestion in HF. Incorporating hepatic and portal Doppler indices into standard evaluation protocols may improve risk stratification, optimize decongestion therapy, and guide management. Further prospective randomized and outcome-driven studies are required before VExUS-based therapeutic thresholds can be universally recommended and define prognostic thresholds.

## 1. Introduction

**Figure 1:**
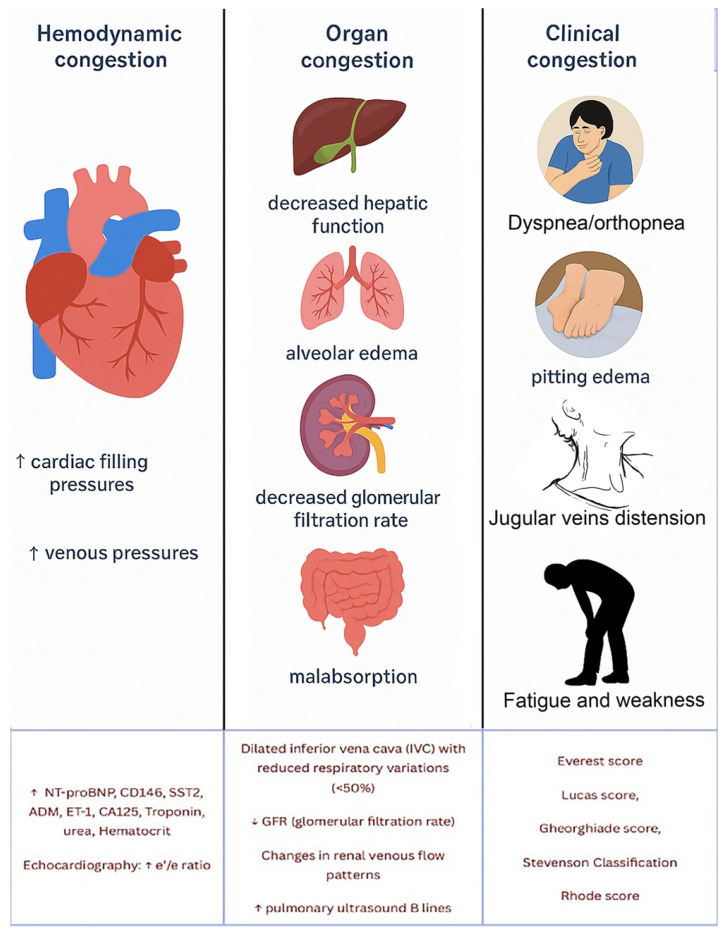
Aspects of fluid overload and their complex interplay typically seen in heart failure. This conceptual framework illustrates the transition from compensated haemodynamic stress to overt clinical decompensation, now with a clarified alignment between the stages of congestion and the diagnostic modalities that most directly interrogate them. The upper panel delineates the pathophysiological cascade. It begins with “Hemodynamic Congestion,” a subclinical state where neurohormonal activation leads to elevated ventricular filling pressures, yet the patient remains asymptomatic. This is a pivotal, often undetected phase where compensatory mechanisms are still operative. As the condition progresses, the sustained elevation in central venous and pulmonary capillary pressures culminates in “Organ Congestion,” the palpable manifestation of fluid extravasation into the interstitial and visceral spaces, leading to the classic signs and symptoms of heart failure. The lower panel is now explicitly structured to mirror this physiological progression, visually linking categories of congestion to their primary diagnostic tools. On the left, “Hemodynamic Congestion” is primarily identified by modalities that assess central pressures and cardiac function. Standard Echocardiography provides critical data on chamber dimensions, systolic function, and estimates of filling pressures (e.g., E/e′ ratio), while Circulating Biomarkers (like BNP and NT-proBNP) serve as indirect, systemically released indicators of myocardial wall stress. In contrast, the right side details the tools for direct visualization of “Organ Congestion.” This is the domain of point-of-care ultrasound, which moves beyond inference to image the actual consequences of elevated pressures. Pulmonary Ultrasound directly quantifies pulmonary interstitial edema through the enumeration of B-lines, a marker of “lung water.” Simultaneously, Abdominal Venous Doppler, epitomized by the VExUS protocol, provides a window into the systemic venous circuit, grading the severity of visceral congestion through characteristic flow patterns in the hepatic, portal, and intra-renal veins. This clarified alignment underscores a critical diagnostic paradigm: while haemodynamic tools predict the risk of organ-level pathology, organ-specific ultrasound confirms its existence. By visually and conceptually separating these domains, the figure emphasizes the complementary nature of these modalities in providing a comprehensive bedside assessment, from the initiating haemodynamic insult to its final organ-specific consequences. Diagnosis is achieved integrating multiple diagnostic modalities, with the use of various diagnostic tools, such as biomarkers (e.g., NT-proBNP, CD146, SST2, ADM, ET-1, CA125), echocardiography, lung ultrasound, and congestion assessment scores, to evaluate congestion comprehensively. The upward (↑) and downward (↓) arrows are standard notations for increases and decreases.

Cardiovascular physiology represents a finely tuned equilibrium between the pump function of the heart and the conduit and reservoir functions of the vascular system. The heart’s primary role is to pump blood [1], a task it accomplishes through a complex interplay of extrinsic and intrinsic control mechanisms. Extrinsic regulation involves the autonomic nervous system [2] and neuroendocrine pathways [3], which can rapidly adjust heart rate, contractility, and vascular tone in response to the body’s demands. Intrinsically, the heart adapts via the Frank-Starling mechanism, where an increase in preload enhances the force of contraction [4], and the Anrep effect, which describes a slow, intrinsic increase in contractile force in response to an acute rise in afterload [5]. Together, these systems work to maintain cardiac output and stroke volume, ensuring adequate tissue perfusion [6]. The arterial system is far more than a passive pipeline. It functions as a conduit and a critical pressure reservoir through the Windkessel effect [7]. In large elastic arteries, this effect unfolds in two distinct phases to ensure continuous, non-pulsatile blood flow to the capillaries. During ventricular systole, the arterial walls expand, absorbing a portion of the stroke volume and temporarily storing pressure energy as potential energy within stretched elastin fibers [7]. This passive distension acts as a hydraulic buffer, smoothing the pulsatile pressure waveform and reducing pulse pressure. During diastole, the elastic recoil of the arterial wall converts this stored potential energy back into kinetic energy, propelling blood forward and maintaining perfusion pressure even when the heart is not ejecting [8]. The regulation of blood pressure within this system involves dynamic physiological adaptations operating over short and long timescales [9]. Acutely, endothelial mediators like nitric oxide and endothelins interact with myogenic responses and autonomic baroreflexes to fine-tune vascular tone, controlling resistance and pulse wave reflection [10]. Chronically persistent haemodynamic and neurohormonal stress induces structural remodeling characterized by vascular smooth muscle hypertrophy, collagen deposition, and elastin fragmentation, which collectively increase arterial stiffness and pulse wave velocity [9,11]. These changes impair the baroreflex’s buffering capacity and enhance wave reflection, thereby sustaining hypertension and propagating target-organ damage throughout the cardiovascular system [12,13]. A foundational concept in understanding cardiac output regulation is Guyton’s model of circulatory function [14]. This paradigm posits that cardiac output is primarily determined by venous return, which is itself driven by the pressure gradient between the mean systemic filling pressure (MsfP) and the right atrial pressure (RAP) [15,16]. MsfP represents the pressure within the systemic circulation during circulatory arrest and is determined by the total blood volume and the compliance of the venous system, conceptualized as “stressed” and “unstressed” volumes [17,18]. Venous return can be augmented not only by increasing total blood volume but also by reducing venous compliance, effectively mobilizing unstressed volume into the circulatory system without altering the total quantity of blood [18]. Within this framework, the splanchnic circulation emerges as a protagonist in haemodynamic regulation. It is the body’s principal reservoir of unstressed volume [19]. Anatomically, it is supplied by the celiac trunk and superior and inferior mesenteric arteries and is drained via the portal system into the liver [20]. Due to its extensive venous capacitance in the spleen, mesentery, and hepatic sinusoids, the splanchnic bed can mobilize 600–700 mL of blood centrally in response to physiological stimuli such as exercise or pathological states like haemorrhage [21,22]. This compartment is under sophisticated control, regulated by extrinsic (autonomic, neurohumoral) and intrinsic (metabolic, paracrine) mechanisms [23]. Its high compliance and vast reservoir capacity make it uniquely sensitive to the perturbations of heart failure (HF). In this context, the splanchnic circulation becomes particularly prone to volume shifts and increased venous pressures, thereby amplifying the haemodynamic impact of elevated cardiac filling pressures and serving as a key contributor to the congestive phenotype [21]. In heart failure, venous congestion is not a monolithic event but a progressive phenomenon that evolves through distinct, albeit overlapping, stages. It begins with initial fluid volume expansion, driven by neurohormonal activation. This is followed by a subclinical phase of haemodynamic congestion, characterized by elevated ventricular filling pressures in the absence of overt symptoms [24]. Finally, the condition culminates in clinical congestion, with frank organ edema and symptoms such as dyspnea and peripheral edema due to fluid retention and redistribution [25] (Figure 1). This pathophysiological cascade underscores the disconnect between internal pressure overload and external clinical signs.

The clinical implications of this progression are profound. Clinical congestion remains a primary driver of hospitalizations and re-hospitalizations for patients with HF [25]. Alarmingly, a significant proportion of patients are discharged with persistent signs of congestion, often without achieving meaningful weight loss despite aggressive diuretic treatment [26]. A seminal post hoc analysis of two major trials by Lala et al. [27] found that only 52% of patients were free of clinical congestion (defined as orthopnea or edema) at discharge, and among those who were, 38% developed recurrent congestion within 60 days. These findings suggest that the resolution of physical signs during hospitalization may be a temporary victory and that conventional clinical or even haemodynamic indicators of decongestion may not reliably correlate with sustained symptom relief or improved long-term outcomes. This notion is supported by discordant findings across studies; while physical signs such as elevated jugular venous pressure and a third heart sound possess prognostic value, they often correlate poorly with invasive haemodynamic measurements, which are considered the gold standard for assessing volume overload [28]. Furthermore, studies have demonstrated that changes in symptoms, functional status, or exercise tolerance following treatment have a limited association with changes in underlying cardiac haemodynamics [29]. This highlights a critical lack of sensitivity and reliability in using physical signs alone to identify the presence or relief of congestion in relation to intracardiac pressures. Consequently, it forces a fundamental question in HF management: should clinical decisions be guided primarily by physical signs and symptoms, or by objective haemodynamic data?

The epidemiology of HF underscores the urgency of this question. HF affects approximately 6.3 million Americans according to the CDC [30], and it exhibits notable sex-based differences, being more prevalent in men but often more fatal in women when left untreated [31]. The modern classification of HF has been refined into four stages: At risk for HF (Stage A); Pre-HF (Stage B); Symptomatic HF (Stage C); and Advanced HF (Stage D) [32]. This staging emphasizes the progressive nature of the disease and the importance of early intervention. HF is also phenotyped based on the primarily affected ventricle. In left-sided HF (LHF), impaired systolic and/or diastolic function leads to reduced cardiac output and increased left atrial and pulmonary venous pressures, resulting in the classic symptoms of pulmonary congestion. In right-sided HF (RHF), dysfunction of the right ventricle causes elevated systemic venous pressure, manifesting as peripheral edema, ascites, and hepatic congestion. Chronic hepatic congestion, or congestive hepatopathy, is a major concern; elevated central venous pressure is transmitted retrogradely to the hepatic veins, while reduced cardiac output limits hepatic arterial perfusion. This combination of venous stasis and decreased oxygen delivery produces centrilobular hypoxia, hepatocellular necrosis, and perivenular fibrosis, which may progress over time to so-called cardiac cirrhosis in patients with long-standing HF [33].

**Figure 2:**
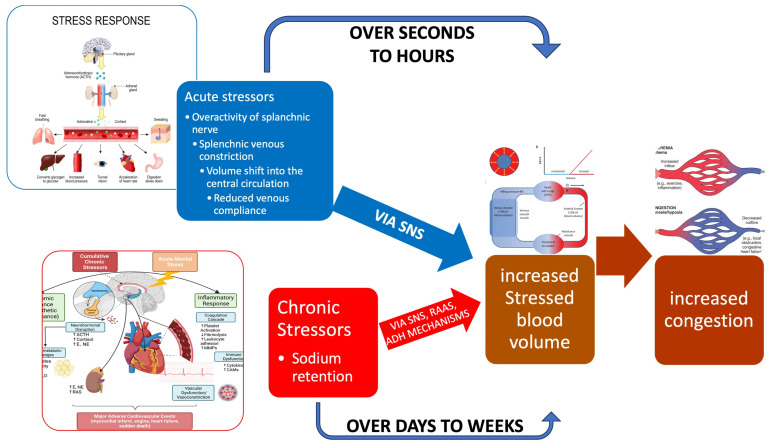
Fast vs. slow mechanisms of congestion. A relatively minor precipitant such as exercise causes a slight increase in sympathetic activity. Depicted on the right are the slow mechanisms that lead to sodium and water retention, causing splanchnic congestion and subsequent increased stressed blood volume. These processes occur slowly over days to weeks. Depicted on the left are the dynamic fast mechanisms that can occur rapidly: Splanchnic venous constriction by the sympathetic activation resulting in volume shifts from the splanchnic compartment to the central compartment, increasing stressed blood volume and causing congestion. These fast processes are often observed in the few days before decompensation. (Yaku et al. [21,42]). Abbreviations: SNS = sympathetic nervous system; RAAS = renin-angiotensin aldosterone system; ADH = antidiuretic hormone.

The splanchnic circulation is intricately involved in both forms of HF. In LHF, neurohormonally mediated venoconstriction can mobilize splanchnic blood volume centrally, transiently increasing left ventricular preload but potentially exacerbating pulmonary pressures. In RHF, venous congestion within the splanchnic bed is a direct consequence of elevated central venous pressure. This visceral congestion worsens outcomes by impairing gut barrier function, promoting bacterial translocation, increasing systemic inflammation, and disrupting the gut microbiome, thereby creating a vicious cycle that contributes to disease progression and is strongly associated with worsened kidney function and infections [21] (Figure 2).

The diagnosis of HF hinges on the recognition of clinical symptoms and signs of fluid overload, such as shortness of breath, fatigue, and leg swelling [34,35]. Echocardiography is the cornerstone of objective assessment, providing critical data on cardiac structure and function. The left ventricular ejection fraction (LVEF) is a key metric used for phenotyping: an EF below 40% indicates heart failure with reduced ejection fraction (HFrEF); an EF between 40% and 49% indicates HF with mildly reduced EF (HFmrEF); and an EF greater than 50% with additional evidence of diastolic dysfunction indicates HF with preserved ejection fraction (HFpEF) [33,36,37]. A more recent category, HF with improved EF (HFimpEF), describes patients with a baseline LVEF ≤ 40% that increases by ≥10 percentage points to a value > 40% [32], highlighting the potential for myocardial recovery. The management of these phenotypes differs, with only SGLT2 inhibitors demonstrating a robust reduction in the risk of HF hospitalization in HFpEF [38,39], while HFrEF benefits from a broader arsenal of neurohormonal antagonists [40,41].

**Figure 3:**
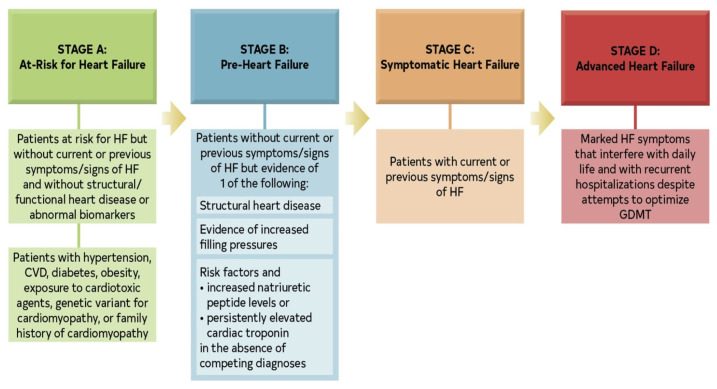
The American College of Cardiology and American Heart Association stages of Heart failure (ACC/AHA Stages); CVD, cardiovascular disease; GDMT, guideline-directed medical therapy; and HF, heart failure.

The New York Heart Association (NYHA) functional classification system [43] (Figure 3) remains a widely used tool for assessing the severity of symptoms and functional capacity, serving as an important predictor of mortality and guiding treatment intensity. HF can present as a new acute event (de novo HF) or as an exacerbation of chronic HF, with acute decompensation being the most common presentation and a major driver of healthcare utilization [44]. Across this spectrum, venous congestion has been consistently associated with worse outcomes, both in acute and chronic settings [45,46].

It is within this challenging clinical landscape that a significant paradigm shift is taking place. There is a growing movement beyond the reliance on traditional, often-insensitive clinical signs toward the use of point-of-care ultrasound for the objective detection of subclinical fluid overload [47]. This approach integrates imaging of the lungs and systemic veins to provide a quantifiable and reproducible window into the patient’s haemodynamic status. The 2023 ESC Cardiomyopathy guidelines [48] formally endorse this shift by integrating lung ultrasound (LUS) for the detection and grading of pulmonary congestion. This move institutionalizes a tool that directly visualizes subclinical congestion, enabling a more proactive and personalized management strategy, particularly in HFpEF which is prevalent within cardiomyopathy cohorts. The scientific literature now compellingly demonstrates that the degree of congestion quantified by ultrasound serves as a robust barometer for both short- and long-term risk. Consequently, these imaging techniques are transitioning from purely diagnostic tools to dynamic guides for therapeutic modulation, allowing for the titration of diuretic regimens based on objective, organ-specific data.

This review aims to systematically examine and synthesize current evidence on the use of conventional ultrasonography—specifically B-mode, colour Doppler, and pulsed Doppler techniques—for the evaluation of splanchnic vascular alterations in patients with heart failure (HF). It seeks to elucidate how ultrasound-based assessment of the hepatic, portal, and mesenteric circulation can provide a non-invasive and dynamic representation of systemic venous congestion, complementing standard echocardiographic and biomarker-based approaches. By integrating data from existing studies, this review intends to identify consistent ultrasonographic patterns—such as portal vein dilation and pulsatility, hepatic vein waveform changes, and bowel wall thickening—that correlate with right atrial pressure, haemodynamic status, and adverse clinical outcomes. The central hypothesis underpinning this review is that the ultrasonographic evaluation of the splanchnic vasculature yields clinically meaningful insights into the pathophysiology and severity of venous congestion in heart failure. It is proposed that these sonographic parameters not only reflect systemic haemodynamic burden but may also serve as valuable prognostic markers and therapeutic guides. Furthermore, the review postulates that integrating splanchnic ultrasound findings into multiparametric frameworks—such as the Venous Excess Ultrasound (VExUS) scoring system—could enhance early detection, improve risk stratification, and optimize decongestive management in both acute and chronic heart failure.

## 2. Materials and Methods

### 2.1. Search Strategy

This systematic review was conducted in accordance with the PRISMA 2020 guidelines. The completed PRISMA 2020 checklist and PRISMA 2020 abstract checklist are available as Supplementary File S1 and Supplementary File S2, respectively. The study was conceptualized by F.G. and D.I. The methodology was designed by D.A. and F.T.; F.G. conducted the software-based literature search, investigation, formal analysis, data curation, and visualization, and wrote the original draft. Validation was performed by E.C., S.M., L.G.; D.I., D.A., and F.T. F.G. also managed project administration and reviewed and edited the manuscript. L.G. provided supervision. All authors have read and agreed to the final manuscript. A comprehensive and systematic search in PubMed was conducted to identify relevant literature, utilizing a preplanned, reproducible search strategy. F.G designed, conducted the database search and analysis The search terms used were: (“Splanchnic Vascularization” OR “Splanchnic Circulation” OR “Abdominal Blood Flow”) combined with (“Heart Failure” [Mesh] OR “cardiac failure” OR “ventricular dysfunction” OR “HFpEF” OR “HFrEF” and “ultrasound” or “colordoppler” OR “Pulsed Doppler” OR “VExUS” OR “Venous Excess Ultrasound” OR “Renal ultrasound” OR “Lung ultrasound” OR “Echocardiography”). We applied no restrictions regarding the date of publication, covering all articles published up to July 2025.

### 2.2. Study Selection

**Figure 4:**
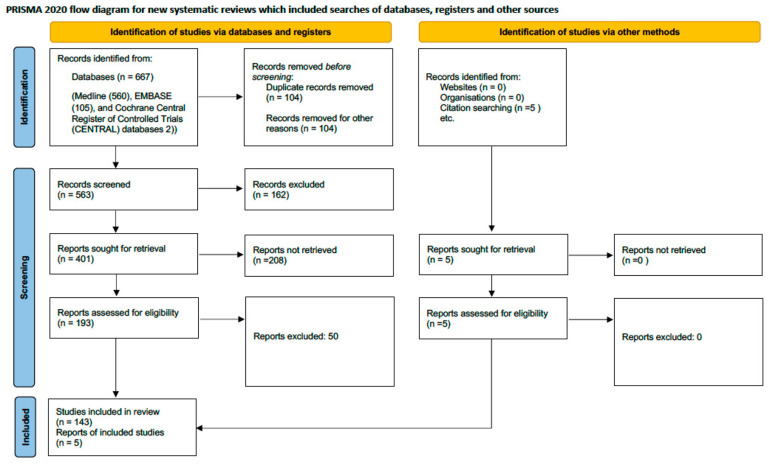
PRISMA 2020 flow diagram illustrating the study selection process [49]. The PRISMA 2020 flow diagram is more than an illustration; it is a cornerstone of methodological transparency. By visually mapping the journey from initial identification of records to the final included studies, it provides an immediate, accessible account of the selection process. This allows readers to quickly assess the scope and rigor of the systematic review, making the diagram an indispensable tool for both reporting and critical appraisal.

We systematically screened titles, abstracts, and full texts to determine their eligibility according to Prisma 2020 statement [49] (Figure 4). The inclusion criteria were: (1) language: articles published in English, Spanish, or Italian; (2) type of study: experimental, observational, and systematic review articles, published as original research in peer-reviewed journals, and limited to human studies; (3) population: adult patients diagnosed with any type of heart failure; (4) focus: studies examining changes in splanchnic vascularization and their impact on heart failure progression or management; (5) outcomes: studies that measured physiological parameters of splanchnic circulation and related these to heart failure outcomes. The exclusion criteria included: (1) case reports, opinion papers, editorials, and studies available only as abstracts; (2) paediatric studies; (3) studies focusing on non-heart failure populations; (4) studies utilizing non-standard methods of assessing splanchnic, cardiac and pulmonary vascularization.

### 2.3. Data Extraction

The study selection process followed the PRISMA 2020 guidelines [49]. Adherence to the PRISMA 2020 guidelines [49] was confirmed throughout the review process, ensuring methodological transparency, reproducibility, and completeness of reporting. All stages—from literature search and study selection to data extraction and synthesis—were conducted in accordance with PRISMA’s structured framework to enhance the rigor and reliability of the systematic analysis.

The initial search strategy identified a total of 667 records from major electronic databases: Medline (*n* = 560), EMBASE (*n* = 105), and the Cochrane Central Register of Controlled Trials (*n* = 2). Supplementary searches contributed an additional 5 records from other sources. Following the removal of 104 duplicates and 104 records excluded for language, 563 unique records underwent title and abstract screening. This screening phase excluded 162 records (because of the limited sample size), leaving 401 reports sought for retrieval. Of these, 208 could not be retrieved (for clinical settings), resulting in 193 full-text reports from the database search being assessed for eligibility. From the database-derived reports, 50 were excluded after full-text assessment for not meeting the eligibility criteria. In contrast, all 29 supplementary reports were deemed eligible. Ultimately, this process culminated in the inclusion of 143 studies, which are documented across the 5 supplementary reports.

The selection process is detailed in the PRISMA 2020 flow diagram (Figure 4). Reviewer disagreements regarding study eligibility and data interpretation were resolved through discussion and consensus with a third independent reviewer to ensure objectivity and methodological consistency. Reference management was performed using Endnote 21, version 21.0.1 (Clarivate Analytics), running under the macOS 15.7 operating system which facilitated accurate data management and duplication control.

#### Risk of Bias and Certainty Assessment

The pronounced heterogeneity among the included studies—spanning design, imaging protocols, and measured outcomes—precluded a meaningful quantitative synthesis and the application of standardized risk-of-bias tools (e.g., Newcastle–Ottawa Scale [50], ROB 2.0 [51]). These tools are most effective when comparing methodologically similar studies. Our approach instead prioritized a rigorous qualitative synthesis. We systematically identified and narratively described key methodological limitations (such as sample size, blinding, and potential for confounding) as they pertained to the findings of individual studies and the overall body of evidence. This nuanced appraisal is woven throughout Section 3 and Section 4, providing a transparent account of the evidence’s strengths and limitations

This review acknowledges the lack of a prospectively registered protocol as a limitation, a measure that guards against bias in hypothesis-driven analyses. However, as a broad, exploratory synthesis of a heterogeneous field, its narrative approach was inherently iterative, making a rigid pre-specified protocol less practical. The risk of selective reporting was lower given the aim to map all evidence, not test a single hypothesis. To ensure rigor, we adhered strictly to PRISMA 2020 guidelines, with an internally defined, pre-planned methodology and full transparency, providing a verifiable audit trail. This foundational overview paves the way for future, protocol-driven reviews as the field matures.

### 2.4. Data Synthesis and Analysis Framework

Given the pronounced methodological and clinical heterogeneity among the included studies—spanning diverse designs (e.g., prospective cohorts, case–control, cross-sectional), patient populations (acute vs. chronic HF, varying HF phenotypes), ultrasound acquisition protocols, and reported outcomes—a formal meta-analysis was deemed inappropriate. Instead, a systematic narrative synthesis was conducted, adhering to established guidelines for the synthesis of heterogeneous evidence.

The synthesis was structured around a pre-defined analytical framework focused on answering the following key questions:

- What are the specific ultrasonographic parameters (B-mode, Doppler) used to assess the splanchnic, cardiac, and pulmonary circulations in heart failure?
- What is the evidence for the association between these parameters and objective measures of haemodynamic congestion (e.g., right atrial pressure, pulmonary capillary wedge pressure)?
- What is the prognostic value of these parameters for clinical outcomes (e.g., mortality, heart failure hospitalization, worsening renal function)?
- How are these parameters integrated into multiparametric protocols (e.g., VExUS), and what is the evidence for the utility of such integrated approaches?

The findings were organized and are presented in Section 3 according to the following thematic categories:

Splanchnic Circulation: Synthesizing evidence on:

- B-Mode Parameters: Bowel wall thickness, inferior vena cava (IVC) diameter and collapsibility, hepatic vein dilation.
- Doppler Parameters: Hepatic vein waveform patterns (triphasic, biphasic, monophasic), portal vein pulsatility and flow direction, intra-renal venous flow patterns.
- Multiparametric Scoring Systems: Synthesizing evidence on the development, validation, and clinical application of the Venous Excess Ultrasound (VExUS) score and its extended (eVExUS) applications.
- Cardiac Evaluation: Synthesizing evidence on the specific echocardiographic parameters (e.g., E/e′ ratio, TAPSE, RVSP) most consistently linked to systemic congestion in the context of the included studies.
- Pulmonary Congestion: Synthesizing evidence on the use of lung ultrasound (B-lines) for diagnosing and monitoring congestion, and its correlation with other systemic congestion markers.

For each finding reported in the Results, the synthesis explicitly states the source to ensure a clear distinction between evidence derived from the included studies and general pathophysiological background. The strength and consistency of evidence across studies are narratively described, and notable contradictions or gaps in the literature are highlighted.

## 3. Results

This section presents a qualitative synthesis of the evidence extracted from the 148 included studies. The following descriptions of ultrasonographic parameters and their pathophysiological correlates are based on the consistent findings reported across the reviewed literature, unless otherwise stated with a direct citation. General background knowledge on ultrasound physics and physiology has been minimized to focus on the synthesized evidence.

A total of 148 eligible studies, encompassing approximately 7000 patients, were included in the final qualitative synthesis. These studies collectively examined splanchnic vascular alterations in acute and chronic heart failure using conventional B-mode, color Doppler, and pulsed Doppler ultrasound techniques. The included literature demonstrated consistent associations between the severity of heart failure and measurable changes in splanchnic haemodynamics, such as increased bowel wall thickness, portal vein dilation and pulsatility, and alterations in hepatic venous flow patterns.

HF and liver disease often coexist, with conditions like “congestive hepatopathy” (liver damage due to elevated right heart pressures) and “cardiogenic liver injury” (liver ischemia due to poor blood flow) contributing to a worsened prognosis. Diagnosis of cardiogenic liver injury is typically based on elevated liver enzymes and imaging, but non-invasive techniques like shear wave elastography may offer earlier detection [52]

Splanchnic circulation plays a key role in regulating blood volume and systemic blood pressure in cirrhotic patients with portal hypertension. Modulating splanchnic circulation has gained attention in liver transplant management, as it can reduce venous congestion, restore central blood flow, and optimize blood volume during surgery. Pharmacologic splanchnic modulation using vasoconstrictors like vasopressin or terlipressin minimizes excessive portal blood flow post-transplant, a critical factor since high portal flow hinders liver regeneration and recovery. Surgical interventions, such as splenic artery ligation, splenectomy, or portocaval shunting, can also achieve this effect. Additionally, splanchnic vasoconstriction supports perioperative renal function by reducing portal pressure and mitigating hyperdynamic circulation, potentially protecting against acute kidney injury in liver transplant patients [53].

The systematic search and selection process, detailed in the PRISMA flow diagram (Figure 4), yielded 148 studies for qualitative synthesis. These studies encompassed approximately 7000 patients with heart failure. Table 1 summarizes the key characteristics of the included literature.

Narrative Summary:

The evidence base is characterized by a temporal evolution from foundational, single-modality Doppler studies in the 1980s–2000s (e.g., investigating portal vein pulsatility) to a recent proliferation of research into integrated, multi-organ point-of-care ultrasound (POCUS) protocols, particularly following the formalization of the VExUS score after 2020. The field is dominated by prospective and retrospective observational cohorts, with a notable lack of large-scale randomized controlled trials evaluating ultrasound-guided therapy. Patient populations are heterogeneous, covering the full acuity spectrum from critical care to outpatient management. The most frequently reported outcome was prognostic stratification for mortality and heart failure hospitalization, underscoring the clinical focus on risk assessment. This methodological and clinical heterogeneity precluded a quantitative meta-analysis but provided a robust foundation for a comprehensive qualitative synthesis of the role of ultrasound across the entire pathophysiological and clinical spectrum of heart failure.

The synthesized evidence reveals a field in rapid and purposeful evolution. The trajectory moves from foundational studies establishing the pathophysiological basis of splanchnic Doppler changes to a contemporary landscape dominated by multi-parametric, prognostic, and phenotyping applications. Two strong trends are particularly notable: first, a significant portion of recent literature specifically addresses the challenge of quantifying congestion in Heart Failure with Preserved Ejection Fraction (HFpEF), where these tools offer a critical window into otherwise elusive haemodynamics. Second, the research has decisively shifted from validating isolated parameters to championing integrated, multi-organ assessment protocols (e.g., VExUS), reflecting the clinical reality of congestion as a systemic syndrome. Despite this progress, the field remains anchored in observational data, highlighting a crucial need for future randomized controlled trials to translate this robust prognostic evidence into proven, guided-management strategies that improve hard patient outcomes.

### 3.1. Ultrasound Measurements of Splanchnic Circulation

#### 3.1.1. B-Mode

##### Bowel-Wall Thickening

HF is often associated with a loss of appetite, and when combined with liver and intestinal congestion, it can lead to complications such as iron malabsorption, malnutrition, and cachexia [77]. Chronic HF patients may also experience increased colonic wall thickness, possibly due to edema and reduced blood flow to the intestines. This change in the gut may alter the microbiota, triggering systemic inflammation that can worsen heart failure and increase the risk of mortality [78,79].

A study by Ikeda et al. [61] explored the relationship between intestinal wall edema, cardiac function, and clinical outcomes in 168 hospitalized HF patients using spiral CT. Their multivariate analysis found that factors like elevated C-reactive protein, lower estimated glomerular filtration rate, reduced lymphocyte count, a higher E/E′ ratio, and altered defecation frequency were independently associated with increased colonic wall thickness (CWT). Moreover, increased CWT was linked to a higher incidence of adverse clinical outcomes, indicating that it reflects reduced cardiac function and can predict poorer long-term outcomes.

Additionally, bowel wall thickness can be assessed through ultrasound, with increased thickness correlating with higher congestion and worse prognosis in HF patients [77,80].

**Figure 5:**
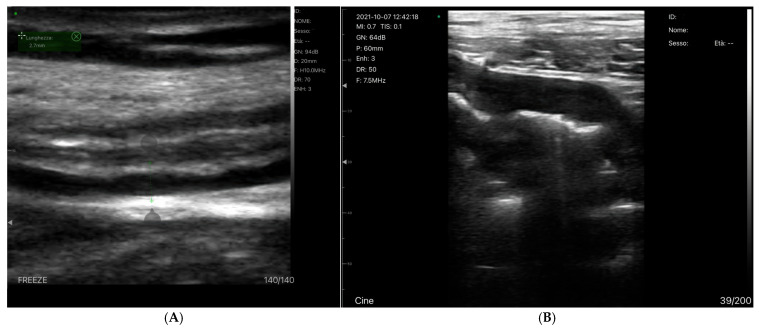
The wall thickness of the ascending colon was significantly different between healthy individuals (Controls) (**A**) and patients diagnosed with acute heart failure (the study group) (**B**), while the wall thickness of the gastric antrum and jejunum showed no significant difference. The ascending colon’s wall thickness is particularly affected because it is supplied by both the superior mesenteric artery (SMA) and inferior mesenteric artery, making it highly dependent on collateral circulation. In acute heart failure (AHF), any interruption of blood flow can impair this delicate system, leading to hypoperfusion, ischemic injury, and potentially intestinal necrosis. The colon is more vulnerable to hypotension and reduced blood flow from AHF compared to other parts of the gastrointestinal tract due to its poorer autoregulatory capacity.

Recently, a Chinese study [68] found that the wall thickness of the ascending colon was significantly different between patients diagnosed with acute heart failure (the study group), and healthy individuals (Controls), while the wall thickness of the gastric antrum and jejunum showed no significant difference. The ascending colon’s wall thickness is particularly affected because it is supplied by both the superior mesenteric artery (SMA) and inferior mesenteric artery, making it highly dependent on collateral circulation. In acute heart failure (AHF), any interruption of blood flow can impair this delicate system, leading to hypoperfusion, ischemic injury, and potentially intestinal necrosis. The colon is more vulnerable to hypotension and reduced blood flow from AHF compared to other parts of the gastrointestinal tract due to its poorer autoregulatory capacity. Other parts of the GI tract have better mechanisms to maintain adequate perfusion. Reduced blood flow to the colon can damage the intestinal barrier, increase epithelial permeability, and promote bacterial translocation, especially of anaerobic bacteria. This contributes to systemic inflammation, edema, and thickening of the colon wall (Figure 5).

A recent Chinese study [68] explored gastrointestinal structural and functional alterations in patients with acute heart failure, using ultrasound as the primary investigative tool. By comparing patients with acute heart failure to healthy controls, the authors examined differences in gastrointestinal wall thickness, vascular dimensions, motility patterns, and self-reported symptoms. The results demonstrated that patients with acute heart failure experienced a heavier burden of gastrointestinal symptoms, particularly related to lower abdominal discomfort and defecatory difficulties. Ultrasound revealed clear evidence of splanchnic congestion, with enlarged hepatic and mesenteric veins and thickened intestinal walls. In parallel, dynamic assessments showed a marked reduction in gastric and intestinal motility, suggesting impaired peristaltic activity across multiple segments of the gastrointestinal tract. Importantly, the study identified significant correlations between vascular parameters and gastrointestinal function: wall thickening of the stomach, jejunum, and colon was positively associated with hepatic venous dilation, while motility indices correlated with superior mesenteric blood flow velocities. These findings link venous congestion and altered mesenteric perfusion to structural changes and functional decline of the gastrointestinal tract. Symptom severity, particularly reflux and abdominal complaints, also showed meaningful associations with both vascular and motility parameters.

Overall, the evidence supports the concept that acute heart failure exerts measurable effects on the gastrointestinal system, mediated through venous congestion and impaired perfusion. The use of ultrasound allowed noninvasive characterization of these alterations, highlighting its potential role as a bedside tool to evaluate gastrointestinal involvement in heart failure. Clinically, these insights reinforce the importance of considering gut function in the management of acute decompensated heart failure and suggest that ultrasound-based monitoring could help guide tailored therapeutic strategies. While these findings highlight the gut as a target organ in HF, the measurement of bowel wall thickness is not yet standardized for routine clinical use and is primarily featured in research settings. In contrast, Doppler-based flow assessments have more robust validation

##### Venous Congestion

**Figure 6:**
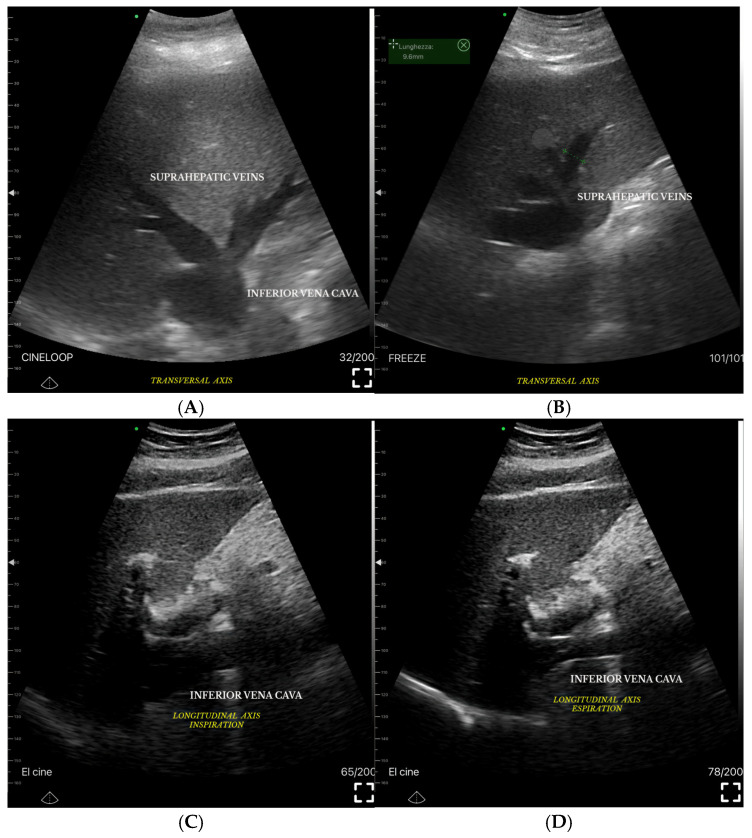
The Cava Vein can be seen in transverse epigastric scan along its short axis (**A**). In the same scan, the Suprahepatic veins can also be appreciated (**B**). By rotating the probe 90°, the inferior Vena Cava can be seen along its long axis (**C**) with a larger caliber during inspiration (due to the depression created inside the abdomen during this maneuver) and a smaller caliber during expiration (**D**).

***Grayscale findings***: Grayscale ultrasonography is the primary method for imaging chronic liver disease (CLD). Key findings include hepatomegaly and enlargement of the venous structures, such as the Inferior Vena Cava (IVC), Sovrahepatic Veins (SV) and Portal Vein (PV). These veins often show reduced or absent collapsibility during inspiration, which is an important indicator of the patient’s fluid status [81]. The IVC’s diameter and collapsibility can be measured using B-mode or M-mode, with normal IVC collapsibility greater than 50% [82] (Figure 6).

The collapsibility index (IVC-CI) helps assess fluid status, with specific measurements correlating to RAP (Table 1). For example, a maximum IVC diameter < 2.1 cm with >50% collapse indicates RAP of 0–5 mm Hg [83]. In liver disease, especially cirrhosis and fibrosis, changes in the liver parenchyma cause alterations in the venous profile, making veins appear thin and serpentine [84]. Portal hypertension is diagnosed when the portal vein pressure exceeds 12 mm Hg or when the pressure gradient between the portal and hepatic veins is >4–6 mm Hg. In chronic hepatic disease, this portal hypertension is typically caused by increased resistance in the right atrium, which affects venous outflow [55].

While direct measurement of hepatic venous pressure gradient (HVPG) can be done through interventional radiology, ultrasound (US) offers a non-invasive way to detect portal hypertension [85]. US signs of portal hypertension include dilatation of the portal vein (>13 mm), porto-systemic collaterals, reversed blood flow in the portal vein, reduced respiratory variation in the splenic and superior mesenteric veins, reduced portal vein velocity, increased congestion index, splenomegaly, and ascites [86,87]. These features help in diagnosing the severity of portal hypertension in CLD [88] (Table 2).

#### 3.1.2. Ecocolordoppler and Spectral Velocity Variations

Doppler ultrasonography (US) is usually the first-line modality for evaluating flow in liver vessels [59]: The vessels usually studied are the portal vein, the suprahepatic veins and the hepatic artery.

##### Portal Vein

Based on a review of the provided literature, the incidence of liver dysfunction and cirrhosis in the context of heart failure is a significant and clinically relevant phenomenon. The relationship is bidirectional, where cardiac dysfunction can lead to hepatic injury, and pre-existing liver disease can influence cardiac outcomes. In patients with chronic heart failure, the prevalence of congestive hepatopathy, a chronic liver condition that can progress to fibrosis, is estimated to range substantially from 15% to 65% [89,90]. This condition develops in the setting of long-standing systemic venous congestion, which is a hallmark of right-sided or biventricular heart failure. Conversely, in the acute setting, the incidence of acute cardiogenic liver injury, also known as ischemic hepatitis or “shock liver,” is estimated to be between 20% and 30% in patients presenting with acute heart failure [91]. This acute injury results from a combination of passive venous congestion and sudden arterial hypoperfusion due to cardiac, circulatory, or pulmonary failure. Furthermore, the prognostic significance of liver dysfunction in heart failure patients is underscored by studies such as that by Wang et al. [92], which demonstrates that the Albumin-Bilirubin (ALBI) score, a marker of liver function, is independently associated with an increased risk of all-cause mortality in intensive care unit patients with heart failure, highlighting the critical interplay between these two organs.

The anatomy of the portal vein (PV) is assessed using B-mode imaging, with the PV located in the hepatoduodenal ligament, behind the hepatic artery and bile duct. It can be identified by tracing the splenic vein to the right until it joins the superior mesenteric vein [72]. If the PV is hard to visualize in a supine position, the patient should be examined laterally. This method shows the PV in 97% of cases, and failure to visualize it may suggest portal vein thrombosis. However, B-mode imaging is not highly accurate for detecting thrombosis or tumour invasion, so Doppler imaging is recommended for confirmation. The absence of colour flow in the PV on Doppler imaging is highly sensitive and specific for diagnosing thrombosis [93].

**Figure 7:**
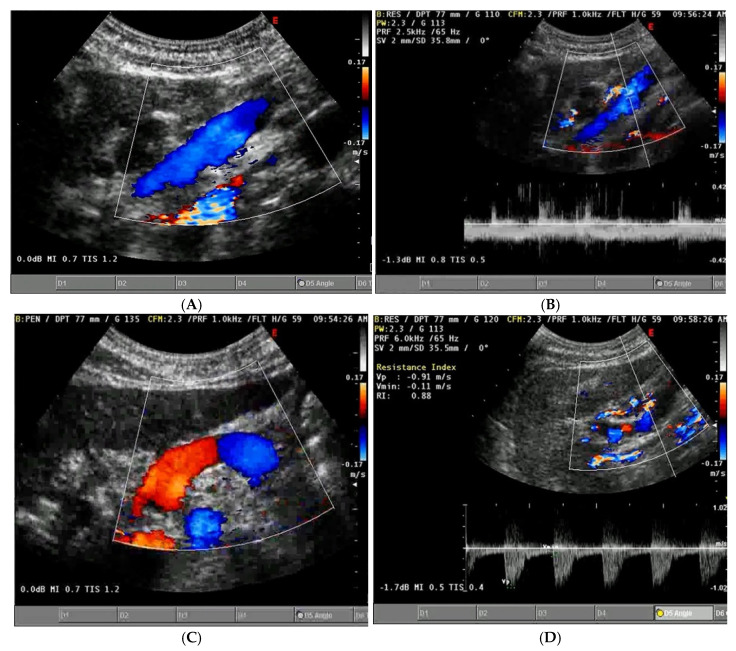
The Portal Vein can be examined via the right subcostal approach: the blue color in this scan indicates a hepatopetal flow (**A**); the evaluation of the same vein with pulsed Doppler confirms a hepatopetal and still biphasic flow (**B**); the Splenic Vein shows a double coloration of the flow on colour Doppler (dependent on the direction of the flow relative to the vessel scanning point) but still demonstrates hepatopetal vascularization (**C**); the pulsed Doppler study of the hepatic artery reveals a high resistance index (**D**).

The normal diameter of the PV is less than 10 mm, with slight increases due to food intake or respiration. In portal hypertension, the PV dilates to over 13 mm and shows little change with respiration. Congestive heart failure can also cause PV dilation, but the IVC will also dilate and the blood flow will be pulsatile in both the portal and hepatic veins. A normal hepatic vein waveform is triphasic, while in portal hypertension, it is often biphasic or monophasic [94]. (Figure 7). The direction of blood flow in the PV is typically towards the liver (hepatopetal) and can be monitored using colour Doppler, with an accuracy of 83% in determining direction of portal blood flow when compared to gold standard angiography [95]. Hepatofugal flow (away from the liver) suggests portal hypertension [96].

**Figure 8:**
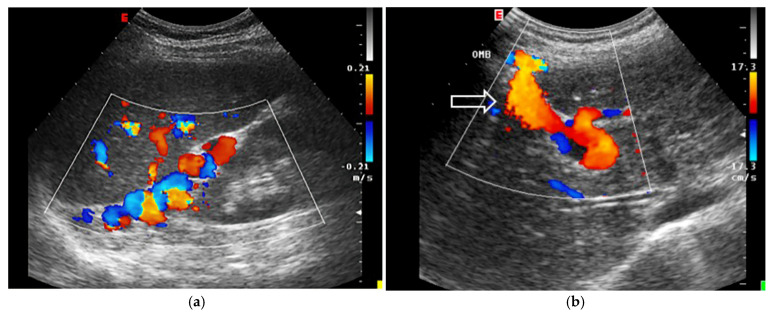
Left (**a**): coronary vein: This Doppler ultrasound image shows the left coronary vein with increased caliber and blood flow velocity, consistent with portal hypertension. The presence of hepatofugal flow and prominent collateral circulation indicates elevated portal venous pressure, suggesting portosystemic shunting and advanced hemodynamic changes secondary to chronic liver disease. and (**b**) Paraumbelical vein: The ultrasound demonstrates a patent paraumbilical vein, indicating recanalization due to portal hypertension. This finding reflects the development of portosystemic collateral circulation, allowing blood to bypass the liver. Paraumbilical vein patency is a characteristic sonographic sign of longstanding portal hypertension and correlates with elevated portal venous pressure.

Additionally, collaterals such as the left coronary vein, paraumbilical vein, splenorenal collaterals, and gastroesophageal collaterals should be assessed (Figure 8). A diameter of over 5 mm in the left coronary vein suggests portal hypertension [97], and a dilated paraumbilical vein is a sensitive indicator of the condition if hepatofugal flow is present [98].

The velocity of blood flow in the portal vein (PV) is measured using Doppler tracings, with normal flow ranging from 15 to 20 cm/s (0.15–0.20 m/s) [99]. After assessing PV diameter and flow velocity, other haemodynamic measurements can be made. In portal hypertension, blood flow increases while velocity decreases [100], and the congestion index [101], which combines both velocity and PV diameter, is a more reliable marker for diagnosing portal hypertension (PHT).

To understand portal venous flow, two key concepts are important. First, normal flow should always be antegrade (toward the transducer), which creates a waveform above the baseline. Second, hepatic venous pulsatility is partially transmitted to the portal veins through the hepatic sinusoids, explaining the cardiac variability seen in the portal venous waveform. It is also important to note that the flow velocity in the portal vein is relatively low (16–40 cm/s) compared to the hepatic artery [59].

The normal portal venous waveform gently undulates and remains above the baseline [102]. The peak velocity (V1) occurs during systole, while the trough velocity (V2) occurs at end diastole. This variation is influenced by atrial contraction at end diastole, which creates back pressure transmitted through the hepatic veins and sinusoids, leading to a decrease in forward portal venous flow (the trough). In cases like tricuspid regurgitation, portal venous pulsatility may increase, making the waveform resemble an inverted hepatic venous waveform [103].

The degree of waveform undulation can be quantified with a pulsatility index (PI). The PI for the portal vein is calculated differently than for the hepatic artery, using the formula PI = V2/V1, with V1 typically greater than 0.5. Lower PIs indicate higher pulsatility. The terms “antegrade” and “hepatopetal” both describe the normal flow direction in the portal vein [70].

A linear relationship between RAP and portal vein pulsatility index has been observed in patients with acute exacerbations of congestive heart failure (CHF) [65]. A case series by Denault et al. found portal vein Doppler assessment to be a promising tool for detecting end-organ venous congestion in post-cardiac surgery patients. However, variations in the portal vein can occur due to factors like body shape and intrathoracic pressure [104]. Venous congestion causes IVC distension, hepatic venous flow abnormalities, and portal vein pulsatility, along with renal venous Doppler flow issues. These findings were incorporated into the VEXUS scoring system [70,75].

Abnormal (pathologic) portal venous flow can manifest in four main ways:

1. **Increased Pulsatility (Pulsatile Waveform)**: A pulsatile portal venous flow occurs when there is a significant difference between peak systolic and end-diastolic velocities. This is due to abnormal transmission of pressure through the hepatic sinusoids, often caused by conditions like tricuspid regurgitation, right-sided heart failure (CHF), or arteriovenous shunting (as seen in cirrhosis or hereditary haemorrhagic telangiectasia (Figure 9A) [105]. Pulsatility can be differentiated clinically, with right-sided CHF and tricuspid regurgitation identifiable through the hepatic venous waveform and grayscale US showing dilated hepatic veins, unlike in cirrhosis, where hepatic veins are compressed.
2. **Slow Portal Venous Flow**: Slow flow occurs when back pressure restricts forward flow, typically indicating portal hypertension. In these cases, peak velocity is less than 16 cm/s [106]. Causes of portal hypertension include cirrhosis, portal vein thrombosis (prehepatic), and right-sided heart failure (posthepatic). The most specific findings include the development of portosystemic shunts (like a recanalized umbilical vein) and slow or reversed (hepatofugal) flow.
3. **Hepatofugal (Retrograde) Flow**: Hepatofugal flow happens when the pressure in the portal vein exceeds that of the liver, causing flow to reverse and appear below the baseline. This is another indicator of portal hypertension, which can be caused by various conditions, including cirrhosis, right-sided heart failure and other portal vein obstructions [107] (Figure 9).

**Figure 9:**
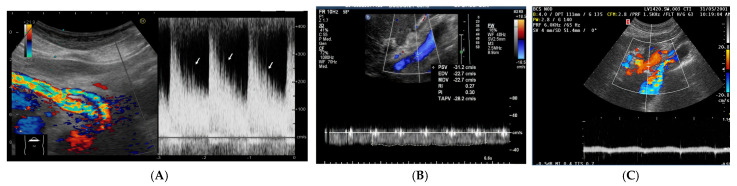
(**A**) Increased pulsatility due to arteriovenous shunting in a case of hereditary haemorrhagic telangiectasia; (**B**) Reduced Portal Flow in a case of cirrhosis (**C**) Hepatofugal Flow of Portal Vein is a late sign of Portal Hypertension. It happens when the pressure in the portal vein exceeds that of the liver, causing flow to reverse and appear below the baseline. This is another indicator of portal hypertension, which can be caused by various conditions, including cirrhosis, right-sided heart failure and other portal vein obstructions.
4. **Absent (Aphasic) Portal Venous Flow:**

**Figure 10:**
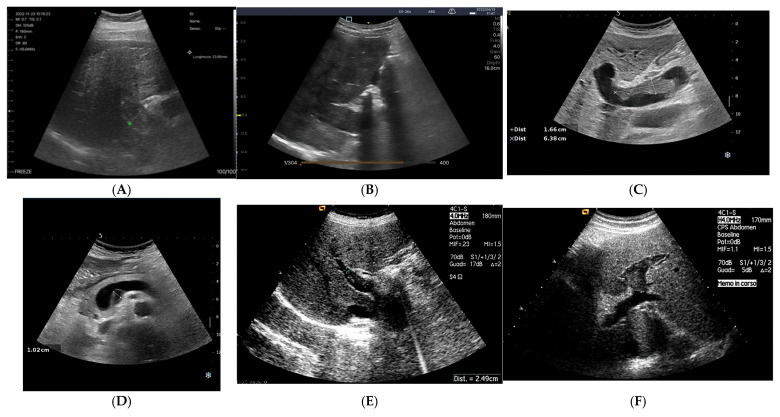
Portal Thrombosis: (**A**) Complete benign (or vascular) Thrombosis; (**B**) Complete malignant; (**C**) Partial Portal Vein Thrombosis; (**D**) Partial Splenic Vein Thrombosis; (**E**) Partial Right branch Portal Vein Thrombosis; (**F**) Partial Left branch Portal Vein Thrombosis.

Absent flow in the portal vein may result from stagnant flow due to severe portal hypertension or occlusive disease, often from thrombosis (either benign—Figure 10A—or malignant—Figure 10B). In cases of occlusive thrombosis, the portal vein will be completely blocked, showing no flow on Doppler [108]. However, in severe portal hypertension, absent flow can occur when the flow is neither hepatopetal nor hepatofugal (stagnant), increasing the risk of portal vein thrombosis. Tumour thrombus (malignant thrombosis) in the portal vein is often associated with a liver mass, and colour Doppler can show arterial (pulsatile) waveforms within the thrombus, known as the “thread and streak sign.” Cavernous transformation, the development of collateral vessels around an occluded portal vein, typically occurs in benign thrombosis and is less common in malignant cases due to the short lifespan of patients with tumour thrombus [108].

Finally, an Italian Study [109] demonstrated the effectiveness of contrast-enhanced ultrasonography (CEUS) and spiral computed tomography (CT) in detecting and characterizing portal vein thrombosis associated with hepatocellular carcinoma (HCC). The study included 50 patients with biopsy-confirmed portal vein thrombi, detected using ultrasonography (US) and colour Doppler US. Among the thrombi, 13 affected the main portal trunk, and 37 involved segmental branches. Both CEUS and CT were performed within a week of the biopsy. Diagnoses of thrombosis (present/absent) and its nature (malignant/benign) were made by experienced readers and compared to pathological findings for accuracy.

Results showed that CEUS detected all 50 thrombi (100%) and correctly characterized 49 of them (98%). In contrast, CT detected 34 thrombi (68%) and correctly characterized 23 of those (68%). CEUS outperformed CT significantly in both thrombus detection (*p* < 0.0001) and characterization (*p* = 0.0001). The study concluded that CEUS is significantly superior to CT for detecting and characterizing portal vein thrombosis complicating HCC and should be considered in the staging of these tumours.

##### Hepatic Veins

To understand the hepatic venous waveform, two key concepts must be recognized [110]. First, the majority of hepatic venous flow is antegrade, meaning it flows away from the liver toward the heart. This flow is typically displayed below the baseline in waveform analysis. Second, changes in pressure within the right atrium influence hepatic veins, and imagining being inside the right atrium helps predict blood flow direction and speed throughout the cardiac cycle.

The waveform can be decoded by understanding the pressure changes in the right atrium during the cardiac cycle [99]. An increase in RAP, such as during atrial contraction near the end of diastole, causes the waveform to slope upward, while a decrease in RAP, such as during early systole, causes the wave to slope downward.

**Figure 11:**
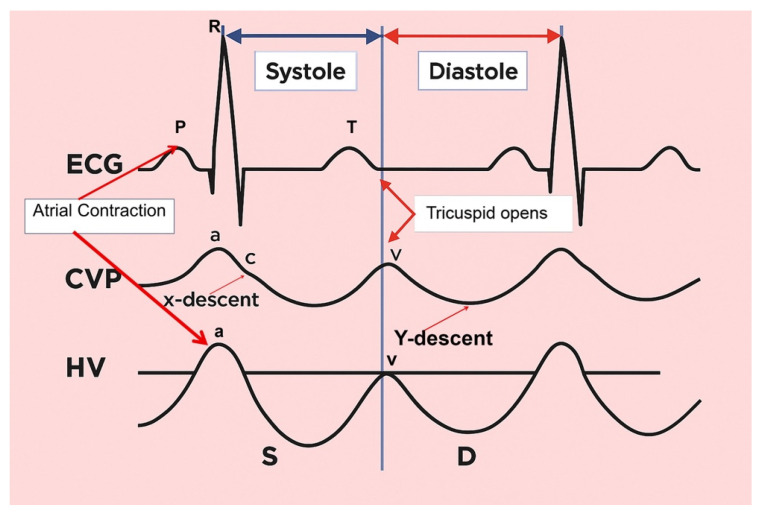
In a normal time-correlated analysis of the electrocardiogram (ECG), central venous pressure (CVP) tracing, and hepatic venous (HV) waveform, the following correlations are observed: The peak of the retrograde a wave corresponds to atrial contraction at the end of diastole. The trough of the antegrade S wave aligns with the peak negative pressure created by the downward motion of the atrioventricular septum during early to midsystole. The peak of the upward-facing v wave correlates with the opening of the tricuspid valve, marking the transition from systole to diastole. This peak may cross above the baseline (indicating retrograde flow) or stay below the baseline (indicating antegrade flow); The trough of the antegrade D wave corresponds to rapid early diastolic right ventricular filling. The overall shape of the hepatic venous waveform resembles a “W,” which can be remembered using the mnemonic “waveform.” This pattern helps in understanding the normal cyclical changes in hepatic venous flow as related to cardiac events.

**Figure 12:**
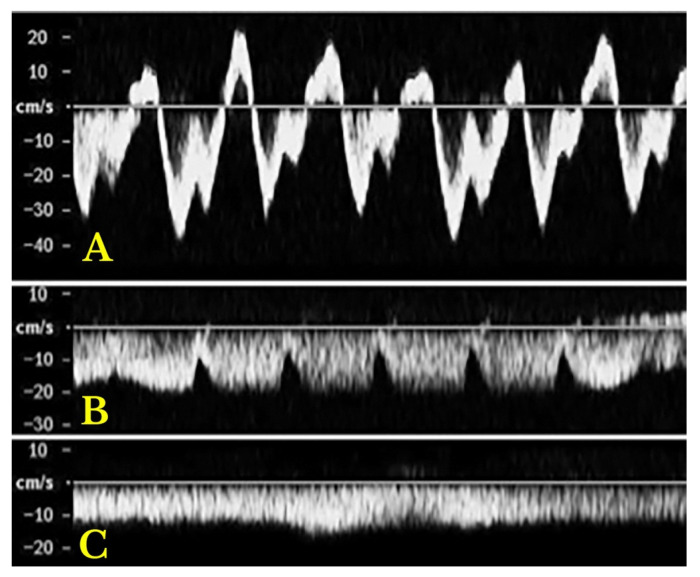
Hepatic vein Doppler waveforms are used to assess blood flow in the hepatic veins. A normal waveform is **triphasic** (**A**), characterized by two hepatofugal peaks (flow away from the liver) and one hepatopetal peak (flow towards the liver) at the top of the panel. Abnormal waveforms include: **Biphasic** (**B**): Lacks the hepatopetal peak, indicating altered flow dynamics, often associated with portal hypertension. **Monophasic** (**C**): Shows a flat waveform, indicating significant disruption to normal blood flow, often seen in advanced portal hypertension or other severe vascular conditions.

The hepatic venous waveform consists of several waves [59] (Figure 11):

1. **The a wave**: Caused by increased RAP during atrial contraction, this upward wave peaks with maximal retrograde flow. It is wider and taller than the v wave in normal states.
2. **The S wave**: Generated by a decrease in RAP during systole, this downward wave represents antegrade flow. It reaches its lowest point at midsystole and is the largest downward wave in the cycle.
3. **The v wave**: This upward wave is produced by increased RAP due to systemic venous return. Its peak marks the transition from systole to diastole, and the wave slopes downward as pressure is relieved during early diastolic right ventricular filling.
4. **The D wave**: The final wave, caused by a decrease in RAP during rapid early diastolic filling, represents antegrade flow. It is smaller than the S wave and reaches its lowest point during maximal diastolic velocity (Figure 12 and Table 3).

Normally, hepatic venous flow is antegrade (toward the heart) and phasic (Table 3).

Variations in flow characteristics affect both venous and arterial vessels [111]. In heart failure (HF), typical findings include marked phasicity in the inferior vena cava (IV) flow, which may even reverse direction. The portal vein (PV) can show increased pulsatility, phases of reverse flow, and interruptions in the flow [112]: the normal spectral Doppler pattern of IV flow is triphasic, consisting of four waves—each linked to different phases of the cardiac cycle and right atrial activity: the “a”, “S”, “v”, and “D” waves (Figure 11). The “a” wave is a positive peak, representing retrograde flow during end-diastolic atrial contraction. The “S” wave is a negative peak, indicating anterograde flow during ventricular systole. The “v” wave is typically an ascending peak and corresponds with the opening of the tricuspid valve, signalling the transition from systole to diastole. The “D” wave is a negative wave, reflecting anterograde flow during early diastolic filling. In HF, both anterograde and retrograde speeds increase, leading to more pulsatile IV flow. In cases of tricuspid regurgitation, both the “a” and “v” waves are increased, while the “S” wave decreases, potentially reversing in severe cases, forming an “a-S-v” complex. Right heart failure (RHF) often shows increased “a” and “v” waves but maintains an appropriate ratio between the “S” and “D” waves [112].

The PV normally shows an anterograde (hepatopetal) flow with smooth undulations caused by cardiac activity, with systolic flow speeds ranging between 16 and 40 cm/s and a pulsatility index (PI) greater than 0.5. In HF, both tricuspid insufficiency and RHF increase pulsatility (<0.5 PI, indicating greater pulsatility), which is transmitted through dilated sinusoids into the PV [55]. Reduced velocity (<12.8 cm/s) in the portal trunk is typical in hepatic cirrhosis and can predict decompensation in compensated cirrhosis, while reverse portal flow indicates poor prognosis in decompensated cirrhosis [85]. Several studies [69,73] suggest that PV pulsatility can detect elevated RAP and estimate its level, with the pulsatility ratio inversely correlated with RAP. Hepatic congestion, ascites, and tricuspid regurgitation are associated with higher PV pulsatility, indicating worsening right heart function [69,113]. Therefore, the PV pulsatility ratio can be a useful sign in assessing HF and monitoring therapeutic responses, especially with bedside ultrasonography [69].

In advanced chronic heart failure (HF) and chronic pulmonary hypertension, severe venous congestion can be difficult to manage, and Doppler profiles indicating venous congestion are often linked to poorer prognoses. A study by Iida et al. (2016) [56] investigated intra-renal venous Doppler profiles in 217 chronic HF patients, finding that certain patterns—such as the short interruption (with both S and D waves) and prolonged interruption (only the D wave)—were independently associated with worse outcomes. The short interruption pattern had a hazard ratio (HR) of 6.85, and the prolonged interruption pattern had a HR of 17.8, both with high statistical significance (*p* < 0.001). These patterns were also linked to worse morbidity and survival in 205 patients with suspected pulmonary hypertension undergoing right heart catheterization, and a lower estimated glomerular filtration rate [114].

Regarding portal vein Doppler assessment, Moriyasu et al. (1986) [54] first described the portal vein pulsatility index (PI) in cirrhotic patients, and later research by Goncalvesova et al. (2010) [69] found that increased pulsatility in portal flow in patients with exacerbated HF corresponds with elevated right ventricular filling pressure. This can help detect elevated RAP and estimate RAP levels. In a study by Ikeda et al. (2018) [115], a higher portal pulsatility index and congestion index at discharge were associated with complications. Similarly, Bouabdallaoui et al. (2020) [67] found that a moderate or severe antegrade pulsatile uninterrupted pattern detected at discharge after a decompensation episode in HF patients was linked to an increased risk of all-cause mortality. Table 3 summarizes these concepts.

#### 3.1.3. Synopsis of the Study of Splanchnic System Congestion: Venous Excess Ultrasound Score (VExUS) and Extended VExUS

Recently, the use of Point-of-Care Ultrasound (POCUS) for haemodynamic monitoring has become a routine practice in perioperative care, primarily focusing on assessing cardiovascular function and fluid responsiveness [116]. While traditional evaluations consider factors like mean arterial pressure and forward flow, understanding systemic venous congestion—indicating increased RAP—is also essential for comprehensive haemodynamic assessment [117]. Venous congestion can result from heart failure, pulmonary vascular resistance, or obstructive conditions, and can lead to organ injury such as kidney hypoperfusion, congestive hepatopathy, and congestive encephalopathy [75].

The integration of these parameters into scoring systems, such as the Venous Excess Ultrasound (VExUS) score, demonstrates high practical and clinical relevance.

The Systemic Venous Congestion may act several consequences: Increased RAP, caused by heart failure or pulmonary conditions, may initially act as a compensatory mechanism, but if excessive, it can cause organ damage by increasing interstitial pressure in organs like the kidneys, which may halt glomerular filtration [117]. Similarly, congestion can affect any organ system, leading to adverse consequences, including, but not limited to, congestive hepatopathy, congestive encephalopathy, and cardio-intestinal syndrome with translocation of lipopolysaccharide [117].

The VExUS grading system represents a significant methodological advancement in the bedside assessment of systemic venous congestion, moving beyond traditional, and often flawed, pressure-based metrics like central venous pressure (CVP) [67].

The system’s physiological validity is rooted in the distinct anatomical and haemodynamic perspectives offered by each vessel. The hepatic vein provides a direct reflection of right atrial pressure, the portal vein, being buffered by hepatic sinusoids, offers an integrated measure, and the intrarenal veins are a sensitive marker of parenchymal congestion and the resultant renal capsule tamponade that impairs perfusion. It is this organ-level assessment that constitutes the primary strength of VExUS. While it correlates with right atrial pressure, its paramount clinical utility lies in its superior ability to predict end-organ dysfunction and its dynamic nature. The waveforms are not static indicators but responsive biomarkers that improve with effective decongestive therapy, providing real-time feedback on therapeutic efficacy, a finding substantiated by associations with improved clinical outcomes such as renal recovery in cardiorenal syndrome.

This multiparametric sonographic protocol classifies congestion into a four-tiered ordinal scale (Grade 0–3) based on an initial evaluation of inferior vena cava (IVC) diameter followed by a comprehensive Doppler Interrogation of the hepatic, portal, and intrarenal veins. A plethoric IVC serves as the gatekeeper for further examination; if present, the severity of congestion is determined by the number of venous systems exhibiting severely abnormal Doppler waveforms, including S-wave reversal in the hepatic vein, >50% pulsatility in the portal vein, or a monophasic pattern in the intrarenal vein. A practical guide “how to perform” is recently described by Turk et al. [118].

Venous congestion assessment is performed in two steps: screening and grading:

1. Screening:

The inferior vena cava (IVC) diameter, measured via ultrasound, can indicate increased RAP, with a diameter greater than 20 mm suggesting congestion. This can be assessed from both long- and short-axis views of the IVC. Alternatively, the internal jugular vein can also be used to estimate RAP [119].

2. Grading

**Figure 13:**
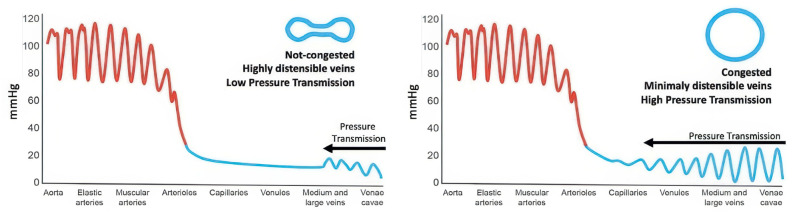
Backwards transmission of pressure from right atrium to peripheral venules and capillaries. Note venous congestion greatly enhances pressure transmission. The backward transmission of pressure from the right atrium to the peripheral venules and capillaries is a critical hemodynamic consequence of right-sided heart dysfunction or failure. When the right atrium becomes overloaded or pressurized, this elevated pressure is transmitted retrograde through the compliant venous system. It is crucial to underline that pre-existing venous congestion greatly enhances this pressure transmission; a distended, engorged venous system loses its compensatory capacitance, acting as a rigid conduit that directly transmits the elevated central pressure to the capillary beds. This can precipitate capillary leakage, tissue edema, and end-organ dysfunction.

Once congestion is detected, its severity is assessed through venous Doppler to evaluate the return flow pattern. Normal veins and venules allow non-pulsatile blood flow, while congestion leads to increased pulsatility [120] (Figure 13).

Doppler assessments in the hepatic, portal, and intra-renal veins help gauge congestion severity and pressure transmission to peripheral organs.

**Figure 14:**
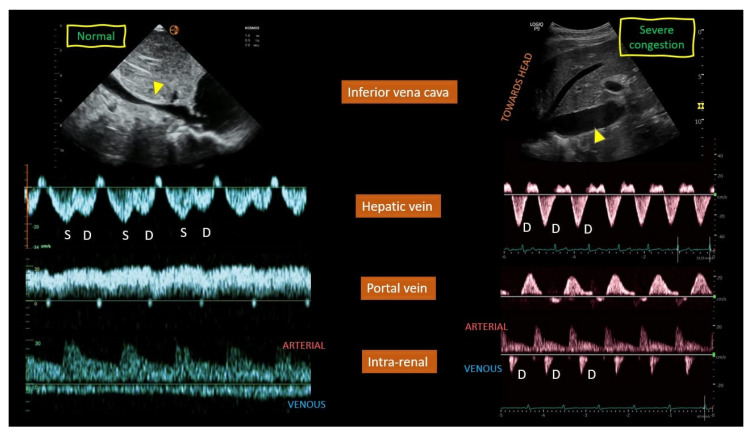
VExUS staging system: Examples of normal and abnormal venous waveforms. S = systole, D = diastole. The Venous Excess Ultrasound (VExUS) staging system classifies venous congestion by analyzing Doppler waveforms from intra-renal and hepatic veins. A normal pattern features dominant antegrade flow during systole (S) and diastole (D), with minimal reversal. In contrast, abnormal patterns signify worsening congestion. Grade 1 shows reduced S-wave dominance. Grade 2 is characterized by a blunted S-wave, making systolic and diastolic flow nearly equal (S ≤ D). The most severe, Grade 3, displays significant systolic reversal, indicating that retrograde flow has become the predominant waveform, a clear marker of severely elevated central venous pressure and systemic congestion.

**Figure 15:**
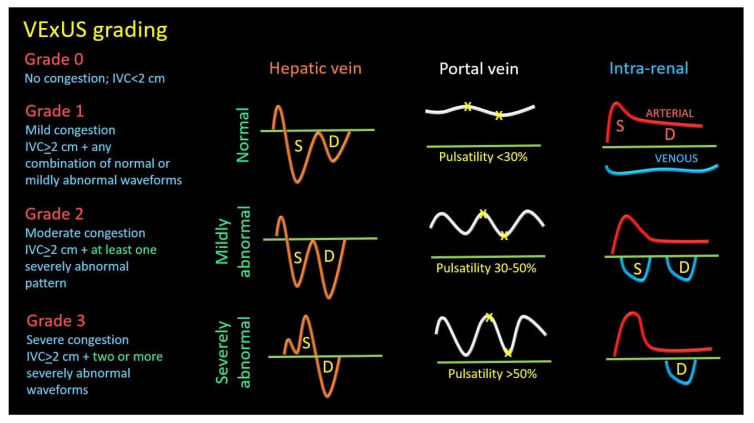
VExUS grading system. S = systole, D = diastole, IVC = inferior vena cava. The Venous Excess Ultrasound (VExUS) staging system is a novel, bedside sonographic technique for grading systemic venous congestion. It moves beyond simple IVC assessment by incorporating pulsed-wave Doppler analysis of hepatic, portal, and intra-renal veins. The system classifies congestion into four stages: no congestion (normal venous flow patterns), and mild, moderate, or severe congestion (Grades 1–3), based on the progressive blunting and eventual reversal of antegrade flow in these vessels. This semi-quantitative approach provides a more nuanced, organ-specific evaluation of volume status, proving crucial for guiding safe fluid management in critically ill patients.

- Hepatic Vein Doppler: In normal conditions, hepatic vein (HV) flow is pulsatile, corresponding to the RAP waveform. Pathologies like right ventricular dysfunction or tricuspid regurgitation can alter HV waveforms, and increasing RAP can reduce venous return during systole, leading to distinct changes in the waveform.
- Portal Vein Doppler: Normal portal vein flow is continuous, but severe venous congestion can cause pulsatility in the portal circulation. The pulsatility fraction (PVPF: [(Vmax − Vmin)/Vmax] × 100) quantifies this, with values above 30% indicating mild abnormalities and above 50% suggesting severe congestion [57]. Elevated PVPF is a strong predictor of acute kidney injury in post-cardiac surgery patients.
- Intra-Renal Vein Doppler: Similar to the portal vein, intra-renal veins show continuous flow under normal conditions, but congestion leads to a pulsatile pattern. This can manifest as a biphasic pattern in moderate congestion and a monophasic pattern in severe cases [114]. Altered intra-renal flow is associated with poor outcomes in heart failure and pulmonary hypertension patients [56,114].
- Grading Venous Congestion: Venous congestion is categorized into grades 0–3 based on waveform alterations, a system known as VExUS (Venous Excess Ultrasound Score) (Figure 14) [63]. This grading system provides a practical method for assessing the severity of venous congestion in clinical settings (Figure 15).

The interpretation of VExUS requires nuanced clinical integration. The IVC diameter cutoff of 2 cm, while useful in the initial validation cohort, must be adjusted for factors such as, athletic history [121], body habitus and elevated intra-abdominal pressure to avoid misclassification [122,123,124]. Furthermore, the system maintains its utility in complex scenarios such as tricuspid regurgitation or pulmonary hypertension. This is because VExUS assesses the final common pathway of congestion—elevated organ afterload—from the downstream, organ-centric perspective, making the upstream aetiology of the elevated pressure less relevant to the interpretation of the organ’s congested state. Consequently, VExUS has catalysed a paradigm shift in critical care and heart failure management, refocusing clinical evaluation from a singular emphasis on fluid responsiveness towards a more holistic consideration of fluid tolerance.

In such cases, it is reasonable to expand the evaluation to include additional veins that may offer insights into systemic venous congestion.

##### Extended Venous Ultrasound (eVExUS) as a Complementary Haemodynamic Paradigm

When the standard Venous Excess Ultrasound (VExUS) examination is precluded by technical limitations or patient-specific factors, the evaluation can be usefully expanded to include other venous systems. This extended approach, often termed eVExUS, encompasses several sonographic parameters that provide corroborative insights into systemic venous congestion, offering a pragmatic and comprehensive bedside assessment when traditional views are inaccessible.

**Figure 16:**
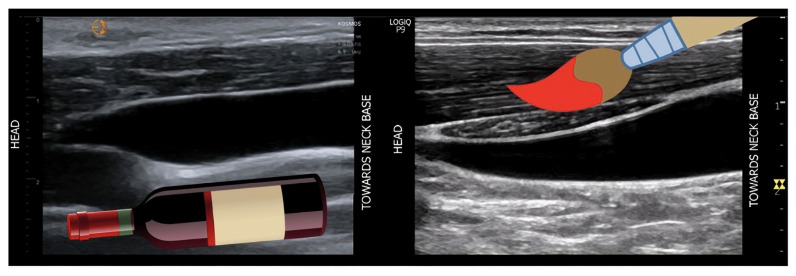
Internal jugular vein collapse point compared to a wine bottle and paint brush. In heart failure assessment, the internal jugular vein’s (IJV) behavior is a key hemodynamic indicator. A useful analogy contrasts its collapse with a wine bottle and a paintbrush. A healthy, non-congested IJV collapses from the top down, much like emptying a wine bottle—the column of blood diminishes from the highest point first. In significant venous congestion, however, the engorged, non-compressible vein collapses along its entire length simultaneously, akin to squeezing the bristles of a paintbrush. This “paintbrush” collapse pattern is a classic visual sign of elevated right atrial pressure and systemic fluid overload.

The ***internal jugular vein (IJV)*** presents a superficially accessible and clinically intuitive alternative, particularly when the subcostal window is compromised [125]. Ultrasound interrogation of the IJV moves beyond the limitations of visual physical examination, employing techniques ranging from static measurement of the venous column height (enhanced by echocardiographic measurement of right atrial depth for improved accuracy) to dynamic assessment of collapsibility [126] (Figure 16). Notably, this method can yield a specific numerical estimate of right atrial pressure, that demonstrates good concordance with invasive measurements and may outperform IVC-based assessment in patients with cirrhosis [127] and elevated intra-abdominal pressure [128]. Furthermore, IJV Doppler waveforms, which closely mirror central venous patterns, offer a functional assessment of right heart haemodynamics. While technically susceptible to transducer pressure and patient positioning, the IJV’s drainage of the cerebral circulation also posits an intriguing, though yet unvalidated, role in investigating venous congestion-related cognitive dysfunction [129].

**Figure 17:**
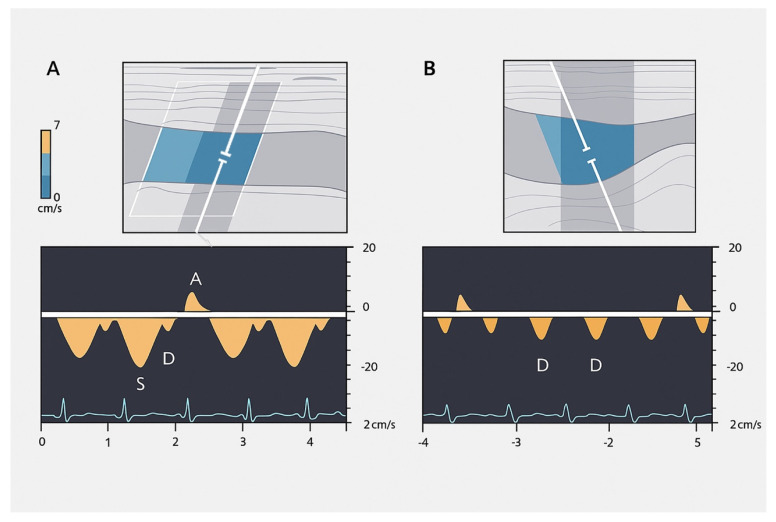
Internal Jugular Vein: In the normal venous waveform (**A**), the expected haemodynamic events of the cardiac cycle are clearly delineated. The S-wave, which represents the systolic phase where atrial relaxation draws blood toward the heart, demonstrates a greater amplitude than the D-wave, which corresponds to the diastolic phase of ventricular relaxation. This relationship (S > D) is indicative of a compliant right atrium and unimpeded venous return to the central circulation. In stark contrast, the abnormal pattern (**B**) signifies a significant deviation from normal physiology, characterized by the complete effacement of the S-wave. This absence suggests a profound dysfunction in the normal systolic suction effect. The waveform is instead dominated by a solitary, prominent D-wave, noted to be below the baseline. This specific positioning confirms that the diastolic flow is still directed antegrade, toward the heart. The transformation into a monophasic pattern consisting solely of this diastolic component is a classic sonographic indicator of severely elevated right atrial pressure. This occurs when the atrium is already overloaded and non-compliant, unable to accommodate the systolic inflow, thereby abolishing the S-wave and leaving only the passive diastolic filling phase to be detected. This pattern is a critical diagnostic finding, often associated with conditions such as right heart failure, cardiac tamponade, or massive pulmonary embolism.

Due to its exceptional technical simplicity, ***femoral vein*** Doppler has emerged as a highly practical component of the extended exam. As venous congestion increases, the waveform becomes increasingly pulsatile, often exhibiting flow interruptions The waveform transitions from a continuous, phasic pattern to a highly pulsatile one with increasing congestion, a phenomenon quantifiable by the Femoral Vein Stasis Index (FVSI) (defined as the percentage of the cardiac cycle during which there is no antegrade flow toward the heart [130]) (Figure 17). Although its distance from the heart confers a lower sensitivity for detecting elevated central pressures, an abnormal pulsatile pattern carries high specificity and is strongly associated with adverse outcomes, including acute kidney injury [131]. This prospective study assessed the diagnostic and prognostic value of the femoral venous stasis index (FVSI) as a non-invasive surrogate for right atrial pressure (RAP) in patients with pulmonary hypertension. Among 101 participants undergoing right heart catheterisation, FVSI demonstrated a strong correlation with invasively measured RAP, offering both high sensitivity for ruling out elevated pressures and high specificity for confirming severe congestion. Importantly, FVSI also predicted adverse outcomes—including hospitalization, treatment escalation, and mortality—over a two-year follow-up, with excellent reproducibility between operators. These findings suggest that FVSI is a simple, reliable bedside tool with both diagnostic and prognostic relevance in pulmonary hypertension management. Its utility is particularly pronounced in scenarios where hepatic waveforms are confounded, such as in severe tricuspid regurgitation or cirrhosis, though it too may be less reliable in the context of intra-abdominal hypertension [132].

Anatomically, the ***superior vena cava (SVC)*** provides the most direct sonographic access to right atrial haemodynamics. Its Doppler waveform, best obtained via a subcostal “bicaval” or suprasternal approach, resembles that of the hepatic vein. Evidence suggests that a systolic-to-diastolic wave ratio derived from the subcostal view correlates robustly with elevated right atrial pressure, and its integration with IVC parameters may enhance diagnostic accuracy. Thus, the SVC serves as a valuable adjunctive data point, especially when other central veins cannot be adequately visualized [133,134].

Finally, the ***splenic vein*** offers a haemodynamic profile analogous to the portal vein, serving as a viable substitute when the standard right lateral intercostal window is obstructed. Early evidence from paediatric [135] and adult cardiac surgery cohorts [136] indicates that its pulsatility index responds to decongestion, mirroring changes in the portal vein. However, its utility is inherently limited in the context of advanced liver disease, where portal haemodynamics become decoupled from central venous pressure.

The evolution of Venous Excess Ultrasound (VExUS) from a research concept to a clinical tool necessitates practical pathways for its application, especially when the standard hepatic vein is inaccessible. The selection of an alternative venous site is not merely a matter of convenience but a deliberate choice balancing physiological fidelity, technical feasibility, and clinical context.

For a rapid, qualitative assessment, the Internal Jugular Vein (IJV) serves as an excellent screening tool. Its superficial location and direct drainage into the superior vena cava make it an immediate barometer of right atrial pressure. The key finding—a transition from a normal, pulsatile, biphasic waveform to a monophasic, continuous flow pattern—is a highly sensitive, albeit non-specific, marker of significant systemic congestion. This transformation occurs because elevated central venous pressure overwhelms the subtle pressure gradients that normally create phasicity, effectively turning the IJ vein into a continuous, high-flow conduit. While its susceptibility to transducer pressure is a notable limitation and it lacks formal validation for the quantitative VExUS grading scheme, its utility lies in speed. In a fluid-challenged patient, a monophasic IJV can instantly confirm the presence of congestion, prompting a more comprehensive assessment or a revision of fluid strategy.

When quantitative, graded assessment is required, the Femoral Vein (FV) emerges as the most practical and physiologically robust surrogate. Its waveform is a direct transmission of the intra-abdominal pressure dynamics and right atrial events, closely mirroring the morphology of the hepatic vein. This allows clinicians to directly apply the established VExUS algorithm, observing the progressive blunting of the systolic wave (S), its dominance by the diastolic wave (D), and the ominous finding of systolic flow reversal. The FV’s primary confounder is intra-abdominal pressure; elevated IAP can artificially blunt the waveform, potentially leading to overestimation of venous congestion. However, its consistency, ease of acquisition, and direct integration into the existing diagnostic framework make it the alternative site of choice for serial monitoring and formal grading when the liver is an acoustic obstacle.

The interrogation of the Superior Vena Cava (SVC), while offering the most direct pre-cardiac data, remains a specialized technique. Its acquisition is technically demanding, often requiring specific patient positioning and sonographer expertise. Its role is thus reserved for complex scenarios where abdominal and peripheral views are non-diagnostic, yet confirmation of profoundly elevated central pressure is critical for management. It is not a routine site but a confirmatory tool in the arsenal of the expert sonographer.

A critical scientific caveat underpins all these assessments: the profound impact of severe tricuspid regurgitation (TR). This pathology fundamentally decouples the waveform from pure “congestion” (volume-driven pressure), as the regurgitant jet during systole creates a dominant systolic reversal wave in all systemic veins. Recognizing this pattern is crucial, as it indicates a primary valvular aetiology for the abnormal Doppler findings, distinguishing it from the volume-overloaded state where systolic forward flow is merely blunted. Thus, a comprehensive venous Doppler examination always involves interpreting the waveform morphology within the full clinical and echocardiographic context.

Table 4 compares the primary alternative sites for Doppler interrogation when standard portal vein assessment is challenging or to provide complementary data. The VExUS grading system (Grade 0–3) is applied to the Doppler pattern observed in the hepatic vein, but the same principles of venous congestion (blunted systolic flow, increased diastolic flow, and systolic-diastolic reversal) can be assessed in these systemic veins.

In conclusion, the eVExUS concept acknowledges the practical challenges of standardized VExUS acquisition and capitalizes on the interconnected nature of the venous system. By leveraging alternative vessels like the IJV, femoral vein, SVC, and splenic vein (Table 4), clinicians can construct a flexible, patient-tailored haemodynamic assessment, ensuring the evaluation of venous congestion remains feasible and informative across a wide spectrum of clinical scenarios (Table 4).

#### 3.1.4. Arterial Hypovascularization

Hepatic Artery: In a healthy physiological state, arteries can adjust their resistance to redirect blood flow to the organs that need it most. When an organ requires more blood, its arterioles relax, resulting in low resistance and adequate perfusion. Conversely, when an organ enters “power save” mode, its arterioles constrict, causing high resistance and redirecting blood flow to other organs. The hepatic artery is a low-resistance vessel and normally has an RI of 0.55–0.7 [59]. Liver disease can cause abnormal changes in the resistance of the hepatic artery, with resistance index (RI) values either elevated (RI > 0.7) or decreased (RI < 0.55). High resistance is a nonspecific finding that may occur in conditions such as the postprandial state, advanced age, peripheral microvascular compression, and chronic hepatocellular diseases like cirrhosis, hepatic venous congestion, cold ischemia (post-transplantation), and transplant rejection at any stage [137]. Low hepatic arterial resistance is more indicative of disease and has a narrower range of potential causes. These include conditions linked to proximal arterial narrowing, such as transplant hepatic artery stenosis (at the anastomosis), atherosclerotic disease (in the celiac or hepatic arteries), and arcuate ligament syndrome. It can also be associated with distal vascular shunts, such as post-traumatic or iatrogenic arteriovenous fistulas, cirrhosis with portal hypertension and related arteriovenous or arterioportal shunts, and Osler–Weber–Rendu syndrome with arteriovenous fistulas [137].

The hepatic artery normally shows an anterograde flow with low-resistance spectral waves (resistance index [RI] 0.55–0.7) and systolic speeds up to 30–60 cm/s. However, hepatic venous congestion can increase arterial resistance (RI > 0.7), due to vasoconstriction in acute settings and fibrosis in chronic cases [112]. Significant correlations have been found between hepatic venous pressure gradient (HVPG) and the resistance indices of the splenic artery, superior mesenteric artery, and renal artery [85,138].

Additionally, hepatic arterial indices are used to calculate early systolic acceleration, which is expressed in meters per second squared (m/s^2^). The slope of the initial systolic upsweep is measured to calculate the acceleration time, which reflects early systolic acceleration [139].

Although changes in arterial and portal flow are not specific to chronic hepatic diseases (CH), they are commonly seen in hepatic cirrhosis [112,140].

In cirrhosis, increased resistance to blood flow is also seen in the hepatic arteries. Hepatic arterial indices, such as the pulsatility index and resistive index, are calculated to assess this resistance. The pulsatility index [141], which uses mean velocity, is more effective than the resistive index (which uses peak velocity) in estimating PHT. These indices can also help detect hepatic arterial stenosis or thrombosis after liver transplantation. Similar indices can be used for the superior mesenteric artery, splenic artery, and renal vessels.

The initial non-invasive assessment of splanchnic hypertension involved the use of the splenic resistance index (RI) and pulsatility index (PI), first studied by Bolognesi et al. [142] in 1996. Their research on 207 patients with portal hypertension showed a strong correlation between these splenic indices and portal vein resistance, measured by hepatic vein catheterization (RI: r = 0.80, *p* < 0.001; PI: r = 0.87, *p* < 0.001). These indices were elevated in cirrhosis patients, indicating portal vein blood flow resistance. In a 2012 follow-up study by the same group [143], splenic PI was measured in 48 heart failure patients and 39 healthy controls. Heart failure patients showed a significantly higher PI (1.19 ± 0.41) compared to controls (0.73 ± 0.11, *p* < 0.0001). The splenic PI correlated with the diameter of the hepatic vein (*p* < 0.02) but not with systemic arterial pressure, cardiac output, or splenic arterial resistance. However, it was significantly associated with right atrial mean pressure (*p* < 0.0003) and right ventricular end-diastolic pressure (*p* < 0.011). Additionally, Yoshihisa et al. [66] investigated liver hypoperfusion and found that a lower peak systolic velocity (PSV) of the celiac artery was correlated with the cardiac index and tricuspid annular plane systolic excursion.

##### B-Mode Renal Ultrasound and Renal Artery

The management of heart failure (HF) perpetually confronts the intricate dance of cardiac dysfunction and renal impairment, a pathophysiological tango formalized as cardiorenal syndrome [144].

While serum creatinine and estimated glomerular filtration rate (eGFR) remain foundational, they are, in essence, lagging indicators—functional readouts that often fail to capture the real-time haemodynamic storm within the renal microvasculature during HF. There has long been a need for a diagnostic tool that could move beyond static anatomy and crude function, offering instead a dynamic window into the pressures, flows, and resistances that define the cardiorenal axis. Renal Doppler ultrasound, and specifically the derivation of the Renal Resistive Index (RRI), has emerged as precisely such a tool, providing a non-invasive, real-time narrative of intrarenal haemodynamics that is deeply enmeshed with the patient’s systemic cardiovascular status [145].

B-mode: In acute heart failure (Type 1 Cardiorenal Syndrome), renal B-mode ultrasound shows normal or unchanged kidney size and appearance, as the issue is acute hypoperfusion, not chronic structural damage and no urinary tract obstruction. The findings are normal because the acute kidney dysfunction is due to poor blood flow (hypoperfusion), not a structural problem within the kidney itself [146]. However, in chronic heart failure (Type 2 Cardiorenal Syndrome), B-mode ultrasound can reveal signs of chronic kidney disease, such as smaller kidneys with reduced cortical thickness and volume, as well as increased parenchymal echogenicity due to fibrosis. This ultrasonographic data (Reduced Cortex-Medulla Ratio) is the result of: The ratio of the kidney’s outer cortex to its inner medulla is decreased. The Increased Echogenicity of the renal parenchyma (tissue) indicates changes like fibrosis [146]. These findings suggest chronic damage and structural changes, often due to long-standing hypoperfusion and congestion.

Having prior kidney ultrasound images for comparison is very helpful in assessing changes over time.

##### Color-Doppler: Renal Resistive Index (RRI)

Conventional B-mode ultrasound offers a static, albeit valuable, morphological snapshot: kidney size, cortical thinning, and increased echogenicity—all markers of chronic, often irreversible, damage. The true revolution lies in the spectral Doppler analysis of intrarenal (interlobar or segmental) arteries. From the characteristic low-resistance waveform, we calculate the RRI [147]:

$$RRI=\frac{Peak\;Systolic-End\;Diastolic\;Velocity}{Peak\;Systolic\;Velocity}$$

Initially, this index was enthusiastically, if somewhat naively, embraced as a direct proxy for intrarenal vascular resistance [148]. However, as our understanding has matured, it has become clear that the RRI is not a simple pressure gauge but rather a complex integrator of a multitude of forces. As Boddi et al. (2015) [145] eloquently synthesize, RRI is the “result of many intra and extrarenal determinants and that renal vascular resistance is only one of these, and not the most important.” This very complexity is what renders it so uniquely informative in a multifactorial disease like HF.

To accurately interpret the RRI in an HF patient, one must learn to read the different voices in this haemodynamic chorus, that are “the Systemic Conductor (Arterial Stiffness and Pulse Pressure) and “The renal percussion” (Venous congestion).

Arterial Stiffness and Pulse Pressure influence RRI. In HF, particularly with preserved ejection fraction (HFpEF), arterial stiffness is a central pathological feature [149]. A non-compliant aorta fails to cushion the cardiac ejection, resulting in a high-pressure systolic surge into the renal microvasculature (elevating peak systolic velocity) and a precipitous pressure drop during diastole (lowering end-diastolic velocity) [150]. Consequently, a high RRI in this context often speaks more to a diseased central vasculature than to intrinsic renal pathology [151,152]. This relationship was starkly demonstrated in renal transplant patients, where RRI correlated with the recipient’s age and pulse pressure (systemic factors) rather than donor age or allograft function (renal factors) [153].

Renal venous congestion in acute heart failure is the element best described by RRI. Elevated central venous pressure is transmitted directly to the renal veins, creating a “back-pressure” that severely impedes diastolic inflow [154]. Conditions which acutely increase renal interstitial or venous pressure (hydronephrosis, acute kidney injury, abdominal hypertension, renal vein thrombosis…) do affect RRI [145]. In decompensated HF, congestion acts as a functional analogue to these conditions, directly elevating RRI [155]. This makes it a powerful, non-invasive barometer of renal congestion, often preceding a rise in serum creatinine.

This integrated haemodynamic burden, reflected by an elevated RRI, carries significant prognostic weight. Ciccone et al. (2014) [74] demonstrated that RRI was an independent predictor of HF progression. Furthermore, in critically ill patients, a high RRI at admission was a powerful independent predictor of mortality and persistent acute kidney injury [156]—a common scenario in decompensated HF. Similarly, RRI ≥ 0.70 is associated with higher mortality in hypertensive chronic kidney disease patients without clinically significant renal artery stenosis after accounting for other significant risk factors [157]. This suggests that the RRI captures an element of pathological vascular dysfunction that is not detected by traditional markers.

The value of RRI extends beyond passive prognostication. Its dynamic nature suggests a role in guiding therapy. One can envisage its use in titrating decongestive therapy; a successful reduction in filling pressures with diuretics or vasodilators should be accompanied by a measurable fall in RRI. Conversely, a persistently elevated RRI might indicate inadequate decongestion or the presence of irreversible structural changes [158]. This aligns with studies showing that interventions like renal sympathetic denervation can reduce RRI, suggesting a direct modulation of intraparenchymal resistance [159].

The RRI is not a magic number. Its interpretation is highly context-dependent. Tachycardia, common in HF, shortens diastole and can artifactually lower RRI, potentially masking the effects of congestion [158]. Furthermore, an elevated RRI in an elderly, hypertensive, diabetic HF patient reflects a lifetime of cumulative cardiovascular damage [160]; it is a holistic indicator, not a specific diagnostic marker for a single disease. Conditions like significant renal artery stenosis must also be considered, as they alter RRI in specific ways (typically causing a tardus-parvus waveform and a lower RRI in the affected kidney) [161].

In conclusion, renal Doppler ultrasound has evolved from a morphological tool into a dynamic haemodynamic stethoscope. The Renal Resistive Index provides a unique, integrative narrative of the cardiorenal axis in heart failure, reflecting the detrimental interplay of arterial stiffness, neurohormonal activation, and venous congestion. It tells a story not just about the kidney, but about the heart that supplies it and the vascular system that connects them. While its interpretation requires experience and clinical correlation, its power as a prognostic biomarker and its potential for guiding therapy position RRI not as a replacement for traditional markers, but as an indispensable complementary tool. It allows the clinician to see the intricate haemodynamic forest, rather than just the functional trees.

##### Clinical Use of Conventional US in Splanchnic Evaluation in Heart Failure

**Figure 18:**
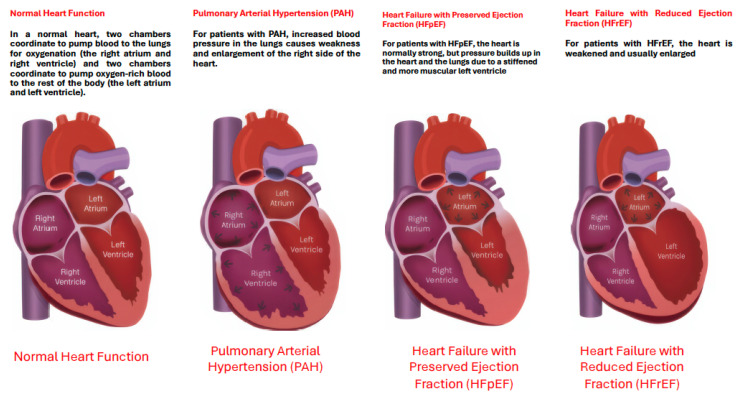
Types of Heart Failure.

Integrated Ultrasound Algorithm for Evaluating Splanchnic Congestion in Heart Failure is proposed in Figure 18. The proposed flowchart synthesizes current evidence into a clinically actionable algorithm for the sonographic assessment of systemic venous congestion, a critical pathophysiological driver of end-organ dysfunction and adverse outcomes in heart failure (HF). This protocol moves beyond traditional, often flawed, pressure-based metrics like central venous pressure (CVP) by providing a direct, organ-level assessment of congestion’s downstream effects. The initial Screening is performed wih the “Gatekeeper Role of the Inferior Vena Cava (IVC)”. The algorithm logically commences with the evaluation of the IVC, establishing it as the essential screening tool. As detailed in the source text, a dilated IVC (>2.0 cm) with reduced collapsibility (<50%) is a well-validated, non-invasive surrogate for elevated right atrial pressure (RAP) [81,82,83]. This plethoric IVC signifies the presence of systemic venous hypertension, serving as the “gatekeeper” that determines the necessity for a more comprehensive investigation. A non-dilated, collapsible IVC effectively rules out significant central venous congestion, obviating the need for further complex Doppler assessment at that time. The second step is constituted by a comprehensive Doppler Interrogation: The Pathophysiological Triad: Upon identifying a congested IVC, the protocol advances to a multi-vessel Doppler interrogation, which forms the core of the Venous Excess Ultrasound (VExUS) score. This step assesses the transmission of elevated RAP backward through the venous system to specific organ beds, each offering a distinct haemodynamic perspective:

Hepatic Vein Doppler: The hepatic veins provide the most direct reflection of right atrial haemodynamics. A normal triphasic waveform (S > D > a) indicates compliant cardiac chambers and unimpeded flow. In HF, progressive congestion manifests as blunting of the S-wave, leading to a biphasic pattern, and in severe cases, S-wave reversal. This reversal indicates a profound transmission of retrograde pressure from the right atrium, severely impeding antegrade systolic flow [59,110,112]. This is a hallmark of severe right ventricular dysfunction or significant tricuspid regurgitation.

Portal Vein Doppler: Under normal conditions, portal venous flow is continuous and hepatopetal (toward the liver), with gentle, low-velocity undulations. The portal circulation, buffered by the hepatic sinusoids, is particularly sensitive to elevated RAP. Significant congestion disrupts this stability, leading to increased pulsatility. A pulsatility fraction (PVPF: [(Vmax − Vmin)/Vmax] × 100) exceeding 50% is a key indicator of severe venous congestion [57]. This finding is not merely diagnostic; it is highly prognostic, being a strong predictor of acute kidney injury in post-cardiac surgery patients and linked to worse outcomes in HF exacerbations [67,69].

Intra-Renal Vein Doppler: The renal venous waveform is typically continuous. The development of pulsatility signifies a pathological elevation in renal interstitial pressure, which can impede glomerular filtration and contribute to cardiorenal syndrome. The progression from a biphasic to a monophasic pattern indicates worsening congestion and is independently associated with increased morbidity and mortality in chronic HF and pulmonary hypertension [114,120]. This finding directly assesses the renal parenchymal consequences of venous overload.

##### The Importance of VExUS Grading and Clinical Interpretation Is Founded from Diagnosis to Management

The algorithm’s power lies in integrating these three Doppler assessments into an ordinal VExUS grade (0–3). This grading system quantitatively reflects the severity of systemic venous congestion:

Grade 0–1 (Mild-Moderate): Indicates minimal or isolated congestion. Management focuses on continuation and optimization of guideline-directed medical therapy (GDMT) with close monitoring.

Grade 2–3 (Severe): Signifies multi-organ venous engagement. This grade is a red flag, strongly associated with a high risk of end-organ injury, particularly acute kidney injury (AKI), and worse clinical outcomes [101,116]. This finding should consider intensification guided by clinical context and serial ultrasound reassessment; randomized evidence for specific VExUS-triggered algorithms is limited, primarily through diuretic therapy, and may necessitate consideration of advanced therapeutic options.

This approach catalyzes a paradigm shift in fluid management, refocusing the clinical question from mere fluid responsiveness (“Will the patient’s blood pressure improve with a fluid bolus?”) to the more holistic concept of fluid tolerance (“Can the patient’s organs withstand their current fluid volume?”).

When standard views are precluded by technical factors (e.g., body habitus, dressings) or patient-specific conditions (e.g., cirrhosis distorting hepatic waveforms), alternative vessels can be interrogated [125,133]. The algorithm incorporates an “escape valve” through the Extended VExUS (eVExUS) concept.

The Femoral Vein offers exceptional technical simplicity. The development of pulsatility or an increased Femoral Vein Stasis Index (FVSI), while less sensitive, is highly specific for severe systemic congestion and is strongly linked to adverse outcomes [130,131].

The Internal Jugular Vein (IJV) provides a clinically intuitive alternative. Its Doppler waveform mirrors central venous patterns, and ultrasound-based measurement of its venous column height offers a more accurate estimation of RAP than physical exam, especially in patients with cirrhosis or obesity [126,127].

The Superior Vena Cava (SVC), though technically challenging, provides the most direct sonographic window to right atrial haemodynamics and can be a valuable adjunct [133,134].

##### Clinical Scenarios

The clinical evaluation of a patient with heart failure often hinges on a critical question: is the primary problem a lack of forward flow (low cardiac output) or a backup of pressure (venous congestion)? Traditionally, answering this relied on imprecise signs and invasive monitors. Today, bedside ultrasound offers a window into this dilemma, beginning with a quick and simple glance at the body’s central venous reservoir: the Inferior Vena Cava (IVC).

Using a basic ultrasound probe placed on the abdomen, the clinician first measures the IVC’s diameter and observes how it breathes. A plump, non-collapsing IVC is like a taut, overfilled hose; it suggests high pressure is backing up from the right heart into the venous system. This initial finding acts as a crucial “gatekeeper.” If the IVC appears normal, significant systemic congestion is less likely, and the clinical thinking might steer toward other causes of the patient’s symptoms. However, a dilated and stagnant IVC raises a red flag, prompting a much deeper investigation. It tells us there is congestion in the central “pipe,” but it does not reveal the pressure’s impact on the vital organs downstream.

This is where the examination escalates into a comprehensive survey known as the Venous Excess Ultrasound (VExUS) exam. Moving beyond simple measurements, the clinician now uses Doppler ultrasound to listen to the flow of blood itself in three key vascular beds: the liver, the gut, and the kidneys. They trace the backward transmission of pressure from the right heart into the hepatic veins, observing how the normal, multi-phased pulse of the heart becomes blunted or even reversed. They then check the portal vein, which normally carries a steady, non-pulsatile stream of blood into the liver; the emergence of a strong pulse in this vessel is a telling sign of severe congestion. Finally, they look at the tiny veins within the kidney, where a healthy, continuous flow is replaced by interrupted, pulsatile patterns, indicating that the congestion is now directly impairing the organ’s filtration system.

Each of these findings is assigned a severity score, and together, they form a composite grade from 0 (no congestion) to 3 (severe multi-organ congestion). This grade is not just an academic exercise; it directly and powerfully informs clinical management. A low grade suggests the patient’s issues may stem more from poor pump function and might cautiously tolerate fluids or other support. A high grade, in contrast, paints a clear picture of a body overwhelmed by venous pressure, mandating an aggressive strategy of decongestion with diuretics and vasodilators to unload the stressed system.

Of course, medicine is never perfectly straightforward. In patients where body habitus, bandages, or bowel gas make it impossible to see the liver or kidneys, the protocol doesn’t simply fail. Instead, it adapts through an “extended VExUS” concept. The clinician can turn to other accessible veins that tell the same story. They can measure the height and waveform of the internal jugular vein in the neck, assess the pulsatility in the femoral vein in the groin, or examine the splenic vein. While each has its nuances, finding abnormal, congested patterns in these alternative sites provides corroborating evidence that the high pressure detected in the IVC is indeed widespread, allowing for confident clinical decision-making even when the standard views are obscured.

In essence, this protocol transforms ultrasound from a simple imaging tool into a dynamic haemodynamic advisor. It provides a logical, step-by-step pathway to move from a simple screening observation to a sophisticated understanding of the patient’s circulatory status, ultimately guiding life-saving therapeutic choices.

The flowchart of Figure 18 synthesizes the key protocols and findings from the document into a clinical decision-making tool: Key Elements Based on the Text: (1) Initial IVC Screening: The IVC is the gatekeeper. A dilated (>2.0 cm) and non-collapsible (<50%) IVC is the primary sign to proceed with a full VExUS exam. (2) Three Key Systems Interrogated: The severity of congestion is determined by Doppler patterns in the: Hepatic Vein: Loss of triphasic pattern, S-wave reversal. Portal Vein: Pulsatility Fraction > 50% (normal is continuous flow).Intra-Renal Vein: Monophasic pattern (normal is continuous flow) (VExUS protocol). VExUS Grading (Grade 0–3): The final grade is based on the number of these three systems showing severe abnormalities. This grade correlates with the severity of systemic venous congestion and prognosis. Clinical Integration: The VExUS grade directly informs management, from optimizing standard therapy to intensifying diuresis and considering advanced strategies due to the high associated risk of organ injury (e.g., AKI). Extended Protocol (eVExUS): If the standard views are impossible, other veins (Femoral, IJV, SVC, Splenic).

### 3.2. Cardiac Evaluation: Echocardiography

In the diagnostic word, echocardiography is paramount for the accurate classification of heart failure into its distinct phenotypes. The assessment begins with the determination of LVEF, which categorizes patients into heart failure with reduced ejection fraction (HFrEF), heart failure with preserved ejection fraction (HFpEF), and the intermediary category of HFmrEF [43] (Figure 18).

In heart failure with preserved ejection fraction (HFpEF), the main pathological process is not an impaired ability of the left ventricle to eject blood but rather a compromised capacity to fill during diastole. The ventricle often exhibits concentric hypertrophy, increased wall stiffness, and impaired relaxation, leading to elevated filling pressures despite a normal or near-normal ejection fraction [162]. Haemodynamically, this results in abnormal left atrial pressure-volume relationships, pulmonary venous congestion, and a marked sensitivity to changes in volume status and afterload; these features are exacerbated by microvascular dysfunction and comorbid systemic inflammation [163]. The myocardial substrate is frequently characterized by increased interstitial fibrosis and microvascular rarefaction, which together impair lusitropic function and reserve capacity under stress [162,164].

By contrast, in heart failure with reduced ejection fraction (HFrEF), the hallmark is systolic dysfunction, defined by a significantly decreased capacity of the left ventricle to generate forward stroke volume; ejection fraction here is typically ≤40% [165]. Structural remodeling often manifests as chamber dilation (eccentric hypertrophy), with thinning of walls, reduced contractile performance, and activation of maladaptive neurohormonal pathways [165]. This maladaptive cycle involves increased wall stress, elevated circulating catecholamines, and activation of the renin–angiotensin–aldosterone system, all of which perpetuate further remodelling and deterioration of pump function [3]. The clinical course is marked by reduced cardiac output at rest and during exertion, diminished contractile reserve, and frequent development of secondary mitral regurgitation due to annular dilatation and papillary muscle displacement [165].

Pulmonary hypertension introduces a distinct but interrelated haemodynamic burden, in which increased pulmonary vascular resistance and right ventricular afterload become central. Recent redefinitions (6th World Symposium) lowered the mean pulmonary arterial pressure (mPAP) threshold to >20 mmHg at rest, with emphasis also on pulmonary vascular resistance (PVR) to distinguish pre-capillary from post-capillary PH [166]. The right ventricle, normally adapted to a low-pressure circuit, responds initially with hypertrophy to preserve stroke volume, but chronic elevation of pulmonary arterial pressure eventually leads to dilation, impaired contractility, and right-sided failure; vascular remodelling including smooth muscle proliferation, endothelial dysfunction, and sometimes in situ thrombosis further worsen the load [150]. As the disease advances, ventricular–ventricular interactions become evident, with right ventricular dilatation shifting the interventricular septum and thereby impairing left ventricular filling, linking pulmonary hypertension pathophysiology tightly to both HFpEF and HFrEF through shared mechanisms of pressure overload, neurohormonal activation, and maladaptive remodelling.

In HFpEF, recent evidence emphasizes the role of metabolic and microvascular abnormalities rather than isolated diastolic dysfunction. HFpEF myocardium demonstrates impaired substrate flexibility, with a shift toward fatty acid utilization and impaired ketone oxidation, changes that can be partially modulated by SGLT2 inhibition but not always with functional benefit [151,152]. In patients with type 2 diabetes and HFpEF without coronary disease, cardiovascular magnetic resonance imaging has revealed impaired myocardial perfusion reserve, abnormal left atrial reservoir function, and compensatory booster-pump activity, all correlating with diastolic strain [167]. In parallel, circulating mediator studies suggest that inflammatory and fibrotic pathways, particularly IL-1 receptor signalling, mediate the link between cardiometabolic disease and HFpEF progression [168]. These findings highlight HFpEF as a systemic, comorbidity-driven phenotype with microvascular, metabolic, and inflammatory underpinnings in addition to traditional stiffness and concentric remodelling (Table 5).

In heart failure with reduced ejection fraction (HFrEF), the defining characteristic remains systolic dysfunction, but novel data underscore how pharmacological interventions modify cardiac structure and function. A recent meta-analysis confirmed that SGLT2 inhibitors consistently induce left ventricular reverse remodelling, with reductions in end-diastolic and end-systolic volumes across trials [169]. Combination therapy with angiotensin receptor–neprilysin inhibitors (ARNIs) and SGLT2 inhibitors in patients with HFrEF or mildly reduced EF has been associated with significant improvements not only in left ventricular ejection fraction (LVEF), volumes, and mass, but also in global longitudinal strain and right ventricular function within three months of therapy [170]. Furthermore, the Heart Failure Optimization Study demonstrated that continued titration of guideline-directed medical therapy beyond 90 days in de novo HFrEF yields progressive LVEF recovery, underscoring the importance of ongoing therapy adjustment [171]. These findings refine the concept of HFrEF remodelling, moving beyond static chamber dilatation toward a dynamic, partially reversible phenotype responsive to modern therapies (Table 5).

Pulmonary hypertension (PH) introduces a distinct haemodynamic burden, with recent studies clarifying both vascular and ventricular remodelling. Three-dimensional imaging of the right ventricle in human and experimental PH has demonstrated that microvascular networks become shorter, more tortuous, and more branched under pressure overload, though capillary density is preserved; importantly, some changes are reversible after unloading [172]. Advanced analyses of right ventricular myoarchitecture in rodent PH models show that the loss of helical fibre range impairs right ventricular–pulmonary arterial coupling and stroke volume generation, distinguishing adaptive from maladaptive remodelling [173]. In clinical settings, multiparametric imaging that integrates CT-based fibrosis scoring with MRI-derived right ventricular strain and volumes has improved diagnostic accuracy for PH in interstitial lung disease [174]. Moreover, right ventricular remodelling assessed by speckle-tracking echocardiography has been associated with prognosis in chronic thromboembolic PH after balloon pulmonary angioplasty [175]. These findings extend the concept of PH from a purely vascular disease to one in which right ventricular remodelling, microvascular adaptation, and ventricular–ventricular interaction determine outcomes *(*Table 5).

The table integrates recent findings on metabolic remodelling, microvascular and perfusion changes, inflammatory pathways, and therapeutic responsiveness (2022–2025). HFpEF is increasingly recognized as a systemic, comorbidity-driven syndrome characterized by metabolic inflexibility, microvascular dysfunction, and limited reversibility. HFrEF remains defined by systolic dysfunction and maladaptive dilatation, but recent evidence highlights the potential for partial reverse remodelling under guideline-directed therapies, particularly with ARNI and SGLT2 inhibitors. PH pathophysiology extends beyond pulmonary vascular resistance to include right ventricular remodelling, microvascular adaptations, and ventricular–ventricular interactions, with certain structural and vascular changes shown to be reversible after unloading or intervention.

However, a contemporary echocardiographic examination delves much deeper [176]. In HFrEF, it characterizes the extent of adverse ventricular remodelling through linear and volumetric dimensions and evaluates concomitant functional mitral regurgitation. Crucially, as demonstrated by Yoshimura et al. [177], the diastolic function parameter E/e′ ratio, a surrogate for left ventricular filling pressure, acquires significant prognostic value even in systolic heart failure (Figure 18); a lower baseline E/e′ emerges as an independent predictor of subsequent systolic recovery and transition to heart failure with improved ejection fraction (HFimpEF), suggesting a myocardium with less fibrotic burden and greater potential for reverse remodelling.

The echocardiographic diagnosis of HFpEF is more nuanced [178], as no single parameter is pathognomonic. Instead, it relies on a constellation of findings that provide evidence of a elevated filling pressures and cardiac remodelling. This includes left atrial enlargement, which serves as a morphological biomarker of chronically elevated left atrial pressure, left ventricular hypertrophy or concentric remodelling, and objective evidence of diastolic dysfunction. Given that many patients with HFpEF manifest normal haemodynamics at rest, diastolic stress echocardiography is often required to unmask the pathological elevation in left ventricular filling pressures, evidenced by an abnormal rise in the E/e′ ratio and pulmonary arterial systolic pressure with exercise. Furthermore, advanced techniques such as speckle-tracking echocardiography are increasingly critical, revealing subclinical systolic dysfunction through impaired global longitudinal strain (GLS) and quantifying left atrial reservoir strain, which is a sensitive marker of atrial myopathy and increased chamber stiffness [178].

Echocardiography is equally vital in the differential diagnosis to exclude specific phenocopies of HFpEF or distinct cardiomyopathies that mandate tailored therapies [179]. It identifies the asymmetric septal hypertrophy and dynamic left ventricular outflow tract obstruction of hypertrophic cardiomyopathy, the concentric hypertrophy with granular sparkling and apical-sparing strain pattern of cardiac amyloidosis, the basal septal thinning and regional wall motion abnormalities in a non-coronary distribution suggestive of cardiac sarcoidosis, and the deep trabeculations of left ventricular non-compaction.

In terms of management and follow-up, echocardiography evolves into a guide for treatment strategy and a monitor of therapeutic response. Serial examinations are used to track the efficacy of guideline-directed medical therapy by documenting reverse remodelling—a reduction in left ventricular volumes, an improvement in LVEF, and a lowering of estimated filling pressures. The confirmation of HFimpEF on follow-up echo carries significant prognostic implications. In HFpEF, echocardiography helps identify dominant phenotypes, such as those with significant pulmonary hypertension or right ventricular dysfunction, which may influence management choices. The integration of echocardiography with cardiopulmonary exercise testing (CPX-ESE) [178] offers a powerful, non-invasive means to dissect the multifactorial contributors to exercise intolerance, linking central cardiac limitations (e.g., failure to augment stroke volume, elevated filling pressures on exertion) to peripheral factors like impaired oxygen extraction. Echocardiography provides a comprehensive haemodynamic and morphologic assessment that is central to the modern management of heart failure. It is the primary tool for establishing aetiology and phenotype, a crucial guide for tailoring management, and an essential instrument for monitoring disease progression, therapeutic response, and long-term prognosis. Its power resides in the synergistic interpretation of a multitude of parameters, from fundamental 2D and Doppler indices to advanced deformation imaging, all contextualized within the patient’s clinical presentation.

#### 3.2.1. The Evolving Role of Echocardiography of Left Heart in Heart Failure

Recent international guidelines and scientific statements have begun to incorporate hepatic and portal vein evaluation. The 2021 ESC Guidelines for the diagnosis and treatment of acute and chronic HF acknowledge the role of intra-abdominal Doppler and organ congestion assessment in the evaluation of patients (McDonagh et al., 2021) [180]. Furthermore, comprehensive review highlighted the assessment of inferior vena cava as a reliable surrogate for elevated right atrial pressure [58] and a predictor of diuretic response and rehospitalization risk [181]. A landmark study demonstrated that a multiparametric echocardiographic protocol including splanchnic vein assessment provided superior prognostic stratification compared to standard echocardiography alone [182].

Echocardiography is a key investigation recommended for evaluating cardiac function. In addition to measuring left ventricular ejection fraction (LVEF), it yields valuable information on chamber dimensions, the presence of concentric or eccentric left ventricular hypertrophy, regional wall motion abnormalities (which may be caused by coronary artery disease or myocarditis), right ventricular function, pulmonary hypertension, valvular abnormalities, and diastolic function. Indeed, the assessment of LVEF, which categorizes patients into heart failure with reduced ejection fraction (HFrEF), heart failure with preserved ejection fraction (HFpEF), and the intermediary category of HFmrEF [43] is only one element in the evaluation of cardiac function. A contemporary echocardiographic examination delves much deeper [183].

**Figure 19:**
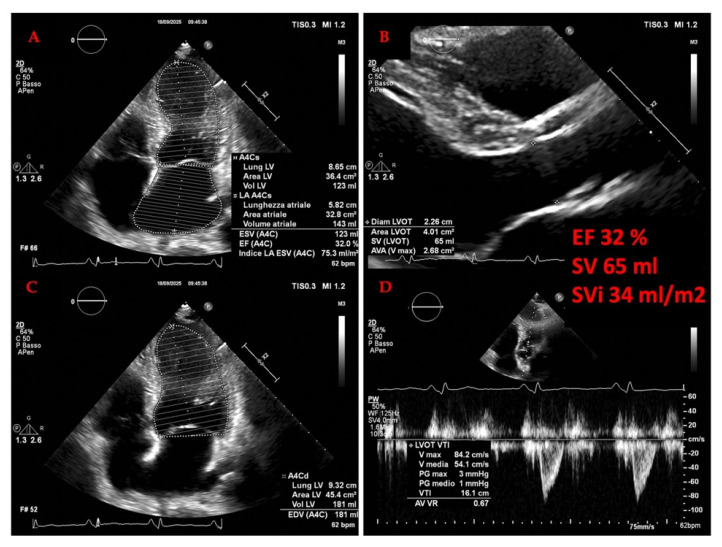
HFrEF secondary to acute myocardial infarction with apical akinesia, characterized by low EF and low SV measured using the VTI method. This case illustrates HFrEF secondary to an acute myocardial infarction with apical akinesia. (**A**) quantifies the severely reduced global systolic function, showing a calculated Ejection Fraction (EF) of 25%. The resulting low forward flow is demonstrated by a low Stroke Volume (SV). (**B**) confirms the Left Ventricular Outflow Tract (LVOT) diameter for cross-sectional area calculation, while (**D**) displays the pulsed-wave Doppler trace at the LVOT, showing a diminished Velocity Time Integral (VTI) of 15 cm. The product of these values (**B**,**D**) yields the low SV. (**C**), showing the dilated left ventricular end-diastolic volume, provides the volumetric basis for the low EF calculated in (**A**). Abbreviations: EF = Ejection Fraction; SV = Stroke Volume; SVi = Stroke Volume index.

**Figure 20:**
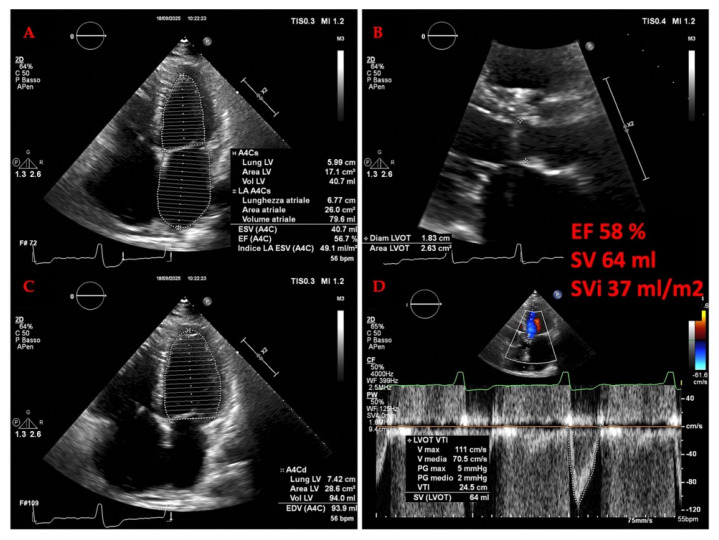
A HFpEF secondary to hypertension, characterized by low EF and low SV measured using the VTI method. Note the right ventricle dilation (**A**,**C**). This case describes Heart Failure with preserved Ejection Fraction (HFpEF) secondary to chronic hypertension. While (**A**) shows a normal Ejection Fraction (EF >50%), confirming “preserved” systolic function, the measured Stroke Volume (SV) is low. This paradox of a normal EF but low SV is elucidated by (**B**,**D**); the SV, derived from the LVOT diameter and a diminished Doppler VTI, is reduced, indicating inadequate forward flow. The hypertensive etiology is evidenced by significant left ventricular hypertrophy (not pictured). Critically, (**A**,**C**) reveal consequential right ventricular dilation and dysfunction, suggesting elevated pulmonary pressures and advanced disease progression. Abbreviations: EF = Ejection Fraction; SV = Stroke Volume; SVi = Stroke Volume index.

**Figure 21:**
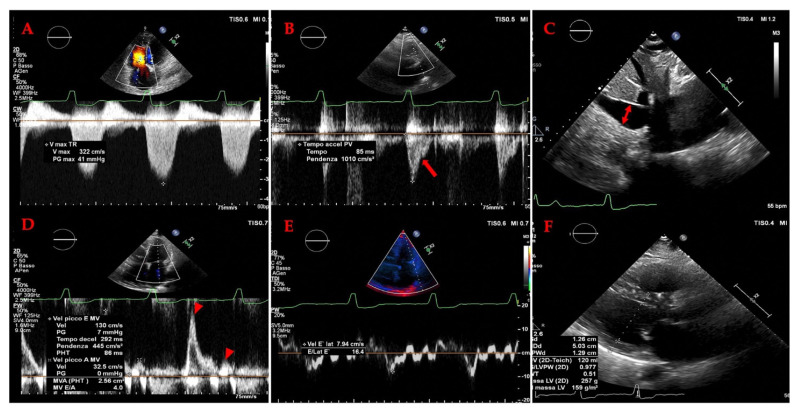
Images from the same case described in Figure 21. The pulmonary hypertension is quantified and confirmed in the next figures. (**A**) (color Doppler of the tricuspid valve) allows for the measurement of high regurgitant jet velocity using continuous-wave Doppler. Using the simplified Bernoulli equation, this velocity is converted into an estimate of pulmonary artery systolic pressure. (**B**): a shortened pulmonary artery acceleration time (AccT < 105 ms). The elevated right-sided pressures are directly visualized in (**C**), which shows a dilated inferior vena cava with poor respiratory collapse, a key sign of elevated central venous pressure and systemic venous congestion. the RVOT profile in (**D**) provide further evidence of the distinctive mid-systolic “notch” (arrow), classic signs of elevated pulmonary vascular resistance and mean pulmonary arterial pressure. (**E**) shows a high E/e’ ratio. This is a cornerstone Doppler measurement, providing a reliable estimate of elevated left ventricular filling pressure (LVFP). It indicates diastolic dysfunction—the impaired ability of the stiff, often hypertrophied LV to relax and fill at low pressures Finally, (**F**) this right ventricular pressure overload leads to right heart dysfunction and systemic congestion. In summary, these figures trace the hemodynamic pathway of HFpEF: from diastolic dysfunction and high LVFP (**E**,**D**), to pulmonary hypertension (**A**,**B**,**D**-notch), and ultimately to right heart strain and systemic venous congestion (**C**).

Although the measurement of EF is extremely useful in clinical practice, it remains rather simplistic, as there are situations in which LVEF does not truly reflect cardiac function. As illustrated in the two cases described in Figure 19, LVEF differs markedly (32% in the case of HFrEF and 56% in HFpEF), yet cardiac performance expressed as stroke volume (SV) and stroke volume indexed to body surface area (SVi) is very similar (around 35 mL/m^2^). These cases highlight that cardiac function cannot be assessed solely based on LVEF but requires a multiparametric evaluation to better characterize ventricular dysfunction and understand the mechanisms underlying venous congestion. As shown in Figure 19 and Figure 20 and emphasized in the recent American Society of Echocardiography (ASE) guidelines for the evaluation of left ventricular diastolic function [184], HFpEF is characterized by impaired relaxation (defined by a lateral e′ ≤ 7 cm/s) which compromises effective systole and the generation of restoring forces that normally act during diastole (like the release of an elastic spring) [185]. This in turn leads to diastolic dysfunction (defined by E/e′ > 14, E/A > 2, and left atrial enlargement > 34 mL/m^2^), which is therefore tightly coupled to systolic dysfunction. The rise in filling pressures, occurring for different reasons in both HFrEF and HFpEF, results in pulmonary hypertension (Figure 21A,B) and venous congestion (Figure 21C).

Crucially, as demonstrated by Yoshimura et al. [177], the diastolic function parameter E/e′ ratio acquires significant prognostic value even in HFrEF (Table 5); a lower baseline E/e′ emerges as an independent predictor of subsequent systolic recovery and transition to heart failure with improved ejection fraction (HFimpEF), suggesting a myocardium with less fibrotic burden and greater potential for reverse remodeling.

The echocardiographic diagnosis of HFpEF is more nuanced [36], as no single parameter is pathognomonic. Instead, it relies on a constellation of findings, included in the HFA-PEFF score [186], that provide evidence of diastolic dysfunction, elevated filling pressures and cardiac remodelling. Furthermore, advanced techniques such as speckle-tracking echocardiography are increasingly critical, revealing subclinical systolic dysfunction through impaired global longitudinal strain (GLS) and quantifying left atrial reservoir strain, which is a sensitive marker of atrial myopathy, increased chamber stiffness and filling pressure [36]. Echocardiography is equally vital in the differential diagnosis to exclude specific phenotype of HFrEF and HFpEF or distinct cardiomyopathies that mandate tailored therapies [187].

#### 3.2.2. The Evolving Role of Echocardiography in Heart Failure: A Focus on the Right Heart and Pulmonary Hypertension (PH)

The recent ASE guidelines [187] underscore a paradigm shift, emphasizing the necessity of a systematic and quantitative evaluation of the right heart, particularly in the context of pulmonary hypertension (PH), which is a critical determinant of outcomes.

Haemodynamically defined as a mean pulmonary arterial pressure (mPAP) > 20 mm Hg, is often first suspected echocardiographically [188]. The estimation of right ventricular systolic pressure (RVSP) from the tricuspid regurgitant jet velocity, combined with an assessment of inferior vena cava dynamics to estimate right atrial pressure (RAP), provides a crucial non-invasive screening tool [189]. However, diagnosis is refined by identifying the phenotypic adaptations of the right heart. Echocardiographic signs such as right ventricular hypertrophy (RVWT > 0.5 cm), dilation (basal RV dimension > 4.1 cm), impaired systolic function (TAPSE < 1.7 cm, FAC < 35%), and abnormal septal geometry (LVEI > 1) help distinguish pre-capillary from post-capillary aetiologies [190]. Furthermore, the identification of a dilated main pulmonary artery (>2.5 cm) or a shortened RV outflow tract acceleration time (AccT < 105 ms) adds weight to the probability of significant pulmonary vascular disease [191].

The transition from diagnosis to management is where echocardiography proves its immense practical value (Figure 20). In patients with heart failure, especially those with preserved EF (HFpEF), distinguishing isolated post-capillary PH from combined pre- and post-capillary PH is paramount, as it directly influences therapeutic strategy [192]. Echocardiographic assessment of left atrial pressure, primarily through the mitral E/e′ ratio, provides an estimate of pulmonary capillary wedge pressure (PCWP), helping to identify a significant post-capillary component [193]. The response to therapy can be monitored serially. For instance, the efficacy of pulmonary vasodilators in pulmonary arterial hypertension (PAH) is assessed not only by a reduction in estimated RVSP but, more importantly, by improvements in right ventricular function and reverse remodelling [194]. Parameters like TAPSE, RV free wall strain, and RV fractional area change serve as markers of therapeutic response. The concept of right ventricular-pulmonary arterial (RV-PA) coupling, elegantly captured by ratios such as TAPSE/RVSP, has emerged as a powerful integrative measure. A low ratio indicates uncoupling, signifying that the right ventricle is failing to adapt to its increased afterload, which is a strong indicator for treatment intensification and a predictor of poor outcomes [194].

**Long-term follow-up** and risk stratification are perhaps the most critical applications of echocardiography in heart failure. Serial studies provide a dynamic window into the disease trajectory. Progressive right atrial enlargement, increasing RV dilation, worsening systolic function, and the development of a pericardial effusion are all echocardiographic harbingers of clinical deterioration and increased mortality [36]. The right atrium, in particular, is gaining recognition as a barometer of chronicity; its size and function, including novel measures like reservoir strain, are strongly prognostic. Advanced techniques such as three-dimensional echocardiography and speckle-tracking strain offer more reproducible and sensitive measures of RV volumes and myocardial deformation, respectively, allowing for earlier detection of subtle changes that may precede overt clinical worsening [186]. In the follow-up of patients with significant valvular heart disease contributing to heart failure, such as functional tricuspid regurgitation (FTR), echocardiography is indispensable for timing intervention and assessing its results, evaluating annular dilation, leaflet tethering, and the haemodynamic impact of the regurgitant lesion [195].

#### 3.2.3. Echocardiography in Advanced Heart Failure

Echocardiography serves as an indispensable, multifaceted tool throughout the continuum of advanced heart failure (AdHF), informing prediction, diagnosis, and clinical management in ways that transcend simple imaging [196]. A standard transthoracic echocardiogram involves a series of images obtained from four windows: parasternal, apical, subcostal, and suprasternal. The parasternal window includes the long-axis (PLAX) and short-axis (PSAX) views. PLAX provides a longitudinal view of the heart, allowing assessment of the left ventricular outflow tract, aortic valve, aorta, left atrium, and left ventricle. PSAX offers a cross-sectional view, enabling evaluation of the left and right ventricles, mitral valve, and tricuspid valve [197].

AdHF prediction: Its role begins even before AdHF is established [198,199]. While patients with non-advanced heart failure carry a significant risk of progression to AdHF (exceeding 9% within two years), echocardiography provides critical predictive insights. Notably, this predictive power lies not in conventional markers like left ventricular ejection fraction (LVEF) or mitral regurgitation—which are often ubiquitous in heart failure populations—but rather in parameters reflecting more advanced myocardial and haemodynamic stress. Severe left atrial dilation (conferring a 35% higher adjusted risk), moderate or severe right ventricular dysfunction (a substantial 66% increased risk), pulmonary hypertension (16% increased risk per 10 mmHg), and elevated left ventricular filling pressures (12% increased risk per 5 E/E‚ Ä ≤ units) emerge as the most potent echocardiographic harbingers of progression to AdHF. Identifying these high-risk patients allows for intensified monitoring and potentially earlier intervention [196].

AdHF diagnosis: The diagnosis of AdHF itself is fundamentally anchored in echocardiographic criteria, reflecting its essential role. Current guidelines [200,201] mandate that at least one specific echocardiographic abnormality must be present: severely reduced LVEF (e.g., 30%), right ventricular failure, severe diastolic dysfunction (alongside elevated natriuretic peptides), non-operable severe valvular disease, or a non-operable congenital defect. The clinical necessity of these criteria is underscored by real-world data revealing that up to 20% of patients discharged after a heart failure hospitalization were misclassified as advanced due to the absence of these echocardiographic findings. A severely reduced LVEF, particularly, 20%, is increasingly recognized as a powerful indicator that may prompt consideration for advanced therapies even without fulfilling other criteria. Beyond LVEF, echocardiography consistently reveals a high prevalence of other abnormalities in AdHF cohorts, including right ventricular dysfunction [187], significantly elevated filling pressures, and significant valvulopathies [202]. However, a definitive AdHF diagnosis also requires the presence of severe symptoms, recurrent hospitalizations, and severe functional impairment.

AdHF Clinical management: Once AdHF is established, echocardiography transitions into a vital guide for clinical management [196], particularly concerning the pervasive challenge of chronic congestion—a major determinant of adverse outcomes. Optimizing diuretic therapy to manage congestion effectively, while avoiding hypovolemia that hinders neurohormonal therapy, requires precise assessment. Echocardiography demonstrably surpasses clinical evaluation alone in accurately detecting and quantifying congestion. Key parameters include the diameter and collapsibility of the inferior vena cava (IVC), which reliably estimate RAP, though interpretation must consider confounding factors like concomitant right ventricular failure or tricuspid valve disease. Promisingly, the Valsalva manoeuvre applied during jugular vein ultrasound (a ratio > 1.6) shows high accuracy in discriminating elevated RAP (>7 mmHg). For left-sided congestion, the ratio of systolic to diastolic pulmonary vein flow (S/D ratio < 1) proves particularly accurate in AdHF, often outperforming the E/E‚ Ä ≤ ratio, which can be unreliable in this advanced population compared to invasive measurements. While extra-cardiac ultrasound (e.g., lung B-lines) has limitations in AdHF, techniques like liver and renal venous Doppler show promise in assessing congestion, warranting further study specifically in this group. Critically, serial echocardiographic assessment of congestion trends should directly inform daily adjustments to diuretic dosing. The potential of echocardiography to actively guide therapy and improve outcomes—by reducing subclinical congestion, lowering natriuretic peptides, and decreasing readmissions—is currently being evaluated in dedicated clinical trials.

In conclusion, Echocardiography remains the principal non-invasive modality for the comprehensive assessment of cardiac structure and function in heart failure (HF). While the determination of left ventricular ejection fraction (LVEF) is indispensable for phenotyping into HF with reduced, mildly reduced, and preserved ejection fraction (HFrEF, HFmrEF, HFpEF), a myopic focus on the left ventricle provides an incomplete haemodynamic picture. A paradigm shift towards a holistic evaluation is critical, one that explicitly recognizes the right heart and systemic venous circulation as the central determinants of congestive pathophysiology. The elevation of right-sided filling pressures, culminating in systemic venous hypertension, constitutes the fundamental haemodynamic link between cardiac dysfunction and the end-organ congestion observed in the splanchnic bed [186]. Consequently, the echocardiographic interrogation must be systematically expanded to quantify the burden on the right heart. The initial evaluation of the inferior vena cava (IVC) provides a direct window into right atrial pressure (RAP). A dilated IVC (>2.1 cm) with diminished respiratory collapsibility (<50%) is a well-validated surrogate for elevated RAP and serves as the foundational marker that should prompt a more detailed assessment of venous congestion [203]. Proximal to the IVC, the right atrium (RA) functions as a compliance chamber and a morphological barometer of chronic pressure overload. RA enlargement (area > 18 cm≤) is not merely a passive consequence but an independent prognosticator, reflecting the duration and severity of elevated RAP. The primary driver of this elevated pressure is often right ventricular (RV) dysfunction. The RV, a thin-walled chamber adapted for a high-compliance, low-pressure circuit, is exquisitely sensitive to increases in afterload, as seen in pulmonary hypertension (PH). Echocardiographic evaluation of the RV, therefore, requires a multi-parametric approach. Linear measures such as TAPSE (Tricuspid Annular Plane Systolic Excursion < 1.7 cm) and two-dimensional measures like fractional area change (FAC < 35%) provide robust, albeit load-dependent, assessments of global systolic function. More recently, speckle-tracking echocardiography has enabled the quantification of RV free wall longitudinal strain, a sensitive marker of subclinical myocardial deformation that is often impaired before overt dysfunction is apparent. The afterload on the RV is predominantly defined by the pulmonary vascular system. The peak velocity of the tricuspid regurgitant jet allows for the estimation of RV systolic pressure, a key surrogate for pulmonary arterial systolic pressure. Supporting signs of PH include a shortened pulmonary artery acceleration time (<105 ms) and RV hypertrophy. Significant tricuspid regurgitation (TR), whether primary or more commonly functional secondary to annular dilation and RV remodelling, creates a volume-overloaded state that further exacerbates RA pressure elevation and systemic venous congestion

### 3.3. Lung Ultrasound and Splanchnic Circulation in Heart Failure

Pulmonary congestion, resulting from the accumulation of extravascular lung water (EVLW), is a fundamental pathophysiological process in heart failure (HF) and the most common cause of symptoms leading to hospitalization [180,204]. The pathophysiological interplay between lung ultrasound findings and splanchnic circulation is fundamentally rooted in their common role as interdependent indicators of systemic volume congestion [47]. The sonographic presence of B-lines within the pulmonary parenchyma is not an isolated phenomenon but frequently represents the thoracic manifestation of a generalized fluid excess, a significant proportion of which is sequestered within the capacious splanchnic vascular bed. Consequently, the initiation of decongestive therapy necessitates the mobilization of this splanchnic reservoir as a critical first step towards achieving meaningful reduction in total body water [205]. Therefore, the serial diminution and eventual resolution of B-lines observed on lung ultrasound provides a dual insight: it signifies the clearance of pulmonary interstitial edema while simultaneously serving as an indirect, yet real-time, biomarker for successful efflux of fluid from the splanchnic compartment. This paradigm elevates lung ultrasound from a purely pulmonary diagnostic instrument to a comprehensive haemodynamic monitoring tool. It offers dynamic feedback on the efficacy of therapeutic interventions designed to offload the entire circulatory system, both central and splanchnic (Table 5). Ultimately, this integrative physiological perspective highlights the potential of lung ultrasound to inform and guide personalized decongestive strategies, targeting the dysfunctional splanchnic haemodynamics that are central to the perpetuation of the congestive state in heart failure.

Traditional methods for its detection, namely physical examination and chest radiography, are hampered by low sensitivity, often reported around 50–60% [206]. This diagnostic gap has been bridged by the emergence of lung ultrasound (LUS), a rapid, non-invasive, and reproducible bedside tool. LUS allows for the semi-quantitative assessment of pulmonary congestion through the detection of sonographic artifacts known as B-lines (formerly termed “comet-tail artifacts” or “lung comets”) [207,208]. These artifacts are reverberations originating from water-thickened pulmonary interlobular septa [209,210,211,212,213,214,215,216,217,218]. Initially validated in critical care to differentiate causes of acute respiratory failure [219], LUS has evolved into an indispensable tool across the entire spectrum of HF care, providing critical insights for diagnosis, treatment guidance, risk stratification, and prognostication [217,220] (Table 5).

#### 3.3.1. Methodological Principles and Protocols

The clinical application of LUS is built upon a clear understanding of its sonographic fundamentals. A normal lung pattern, termed the “A-profile,” is characterized by a hyperechoic, horizontally sliding pleural line and repetitive horizontal artifacts (A-lines) beneath it, indicating normal aerated lung [221,222]. The pathological hallmark of interstitial syndrome is the “B-profile,” defined as three or more B-lines in a single intercostal space. These are laser-like, vertical hyperechoic artifacts that arise from the pleural line, extend to the bottom of the screen without fading, and move synchronously with lung sliding [215,221].

LUS can be performed with any standard echocardiographic machine. While low-frequency transducers (phased-array or curvilinear, 1–5 MHz) are most commonly used for their penetration depth, B-lines can be identified with any probe type with minimal impact on the overall clinical interpretation [221,223]. To standardize the examination, several scanning protocols have been developed, varying in the number of chest zones assessed. The comprehensive 28-zone protocol provides a highly detailed assessment and has been extensively used in clinical research, particularly in chronic and discharge settings [71,224]. However, its time-consuming nature limits its practicality in acute scenarios. Comparative studies have demonstrated that the 8-zone protocol, introduced by Volpicelli et al., offers the optimal balance between efficiency and diagnostic accuracy, with no substantial loss of clinically relevant information [225,226]. This protocol examines two anterior and two lateral zones on each hemithorax. For ultra-rapid assessment in the emergency department (ED), focused 4- or 6-zone protocols, such as those derived from the BLUE (Bedside Lung Ultrasound in Emergency) algorithm, are also effectively employed [201,227]. Expert consensus now recommends examining at least 6 zones in HF patients [228].

#### 3.3.2. LUS in the Acute Heart Failure Setting

##### Diagnostic Application

In the acute setting, particularly the ED, LUS has proven superior to standard diagnostic workups. A large multicentre study by Pivetta et al. (*n* = 1005) demonstrated that a 6-zone LUS approach had a sensitivity of 97.0% and specificity of 97.4% for diagnosing AHF, significantly outperforming clinical workup alone, chest X-ray, or NT-proBNP [60]. A subsequent randomized controlled trial by the same group confirmed that an 8-zone LUS strategy had higher accuracy (AUC 0.95) than a strategy based on chest X-ray and NT-proBNP (AUC 0.87) [229]. A comparative study by Buessler et al. concluded that among various protocols, the 8-zone method (using ≥1 bilateral positive zone) had the highest diagnostic accuracy (C-index 74.0%) and additive value on top of a clinical score for diagnosing AHF in the ED [226]. This high negative predictive value is particularly valuable for rapidly ruling out a cardiogenic cause of dyspnoea.

##### Monitoring Therapeutic Efficacy

The dynamic nature of B-lines makes LUS an excellent tool for monitoring response to decongestive therapy. Multiple studies have documented a rapid decrease in B-line counts that correlates with clinical improvement in dyspnoea and other signs of congestion [230,231,232,233]. This real-time feedback allows for objective titration of diuretic therapy. The kinetics of B-line clearance appear to be faster than the decline in natriuretic peptide levels, especially in patients with renal impairment, highlighting its utility in guiding acute management where biomarker lag can be a limitation [232,233,234].

##### Prognostic Stratification

The prognostic value of LUS is significant at both admission and discharge. A high B-line count upon admission (e.g., ≥45 using the 28-zone protocol) is an independent predictor of short-term adverse outcomes, including death and HF rehospitalization [235,236]. However, the assessment of residual pulmonary congestion at the time of discharge is even more powerfully prognostic. Seminal studies by Gargani et al. and Coiro et al. demonstrated that patients discharged with a B-line count >15 (28-zone) or ≥30 had a dramatically higher risk of readmission or death at 3–6 months [71,237]. This risk persists even when congestion is subclinical (i.e., “dry lung” on auscultation but B-lines ≥ 5 on LUS) and provides incremental prognostic value on top of traditional risk markers like natriuretic peptides and NYHA class [237,238]. Meta-analyses have consolidated this evidence, confirming that elevated B-line counts at discharge are associated with a ~2.5-fold increased risk of adverse events [239].

#### 3.3.3. LUS in the Chronic Ambulatory Heart Failure Setting

In the outpatient clinic, LUS transitions from a tool for diagnosing overt congestion to one for identifying subclinical pulmonary congestion, a precursor to clinical decompensation. Studies have shown a strong correlation between B-line counts and other established markers of haemodynamic stress, including elevated NT-proBNP levels and high left ventricular filling pressures (E/e′ ratio) [240,241].

The prognostic value in chronic HF is well-established for both HFrEF and HFpEF. Platz et al. [62] first demonstrated that in ambulatory patients, a B-line count ≥ 3 (8-zone protocol) was associated with a four-fold higher risk of death or HF hospitalization at 6 months, providing incremental prognostic value over clinical assessment. This finding has been validated across numerous cohorts [242,243]. In HFpEF specifically, LUS has shown similar accuracy to NT-proBNP in predicting adverse outcomes, with a B-line count > 15 signifying a significantly increased risk [244].

This robust prognostic data logically gave rise to interventional trials exploring **LUS-guided therapy**. The LUS-HF and CLUSTER-HF trials randomized HF patients post-discharge to either standard care or a strategy where diuretic therapy was actively titrated based on serial LUS examinations (a B-line count ≥ 3 signifying congestion) [221,245]. Both trials demonstrated that the LUS-guided strategy significantly reduced the combined risk of urgent HF visits and hospitalizations at 6 months (HR ~0.55), with a Number Needed to Treat (NNT) of 5–6. This approach facilitates pre-emptive decongestion, preventing the cycle of clinical deterioration that leads to emergency care.

#### 3.3.4. LUS During Stress Echocardiography

The application of LUS during stress echocardiography provides a unique window into dynamic changes in pulmonary pressures and congestion during exertion, which is particularly relevant in HFpEF where resting haemodynamics may be normal. The development or significant increase in B-lines during exercise is a marker of exertional pulmonary congestion [246]. This phenomenon is strongly correlated with invasively measured increases in pulmonary capillary wedge pressure and impairments in right ventricular-pulmonary arterial coupling [247,248]. Furthermore, stress-induced B-lines carry profound prognostic implications. In HFrEF, a peak stress B-line count ≥ 30 (28-zone) is a powerful predictor of mortality [249]. In HFpEF, both peak B-line count and the change from rest (ΔB-lines) are independent predictors of cardiovascular death and HF hospitalization. A ΔB-lines > 10 during exercise has been identified as a high-risk marker, adding significant prognostic value to clinical and other echocardiographic parameters [250,251]. This identifies a phenotype of patients with limited haemodynamic reserve who are at greatest risk (Table 6).

### 3.4. Guiding Decongestive Therapy: The Role of Serial Ultrasound Assessment

The true clinical power of venous congestion ultrasound lies not only in its diagnostic and prognostic capabilities but also in its potential to dynamically guide patient therapy. Moving from a single snapshot to serial assessments transforms these ultrasound metrics into a practical tool for titrating decongestive strategies in real-time, personalizing management for complex patients.

#### 3.4.1. Establishing a Baseline and Informing the Decongestive Prescription

The initial comprehensive ultrasound exam provides the critical baseline from which therapy is planned. A patient presenting with a significantly dilated and collapsible IVC, extensive B-lines on lung ultrasound, and a VExUS grade of 2 or 3 (indicating severe venous flow abnormality) presents a clear picture of total-body fluid overload. This profile not only confirms the need for aggressive decongestion but also helps risk-stratify the patient. For instance, a high VExUS grade is associated with a higher risk of worsening renal function during diuresis, prompting a strategy that may combine stepped diuretic dosing with close monitoring, rather than a single high-dose bolus.

#### 3.4.2. Monitoring Efficacy and Guiding Titration

Serial ultrasound assessments, which can be performed daily or even multiple times per day in unstable patients, provide objective data on the efficacy of decongestive therapy. The trajectory of change in these parameters is more informative than any single value.

Lung Ultrasound (B-lines): A reduction in the number of B-lines, particularly in a previously affected zone, is one of the most rapid and gratifying signs of successful decongestion. This can be used to titrate diuretic doses; a persistent high B-line profile suggests the need for further or augmented therapy (e.g., adding a thiazide diuretic for “braking”), while clearance of B-lines may signal the opportunity to reduce the diuretic dose or switch to maintenance therapy.

Portal Vein Pulsatility and VExUS: The return of hepatopetal flow pulsatility in the portal vein and an improvement in the venous Doppler pattern (e.g., a shift from VExUS grade 3 to grade 1) are direct indicators of reduced venous congestion and improved right heart dynamics. This is particularly valuable in guiding therapy for patients with right heart failure or cardiorenal syndrome, where clinical signs of congestion can be subtle. The failure of these parameters to improve despite adequate diuretic doses should prompt a search for mitigating factors (e.g., high intra-abdominal pressure, progressive valve dysfunction) or consideration of advanced decongestive strategies, such as vasodilators or ultrafiltration.

IVC Size and Collapsibility: While influenced by many factors, a trend toward a reduction in IVC diameter and an increase in its collapsibility with respiration can support the evidence of successful volume removal gathered from other sites.

#### 3.4.3. The Pragmatic Cycle: A Bedside Workflow for Ultrasound-Guided Decongestion

Translating the static diagnosis of congestion into a dynamic, responsive treatment protocol requires a shift in clinical mindset. The proposed bedside workflow is not a rigid algorithm but a conceptual cycle—a feedback loop that uses serial ultrasound data to personalize therapy in real-time. This approach moves beyond the traditional paradigm of “treat and wait” for lagging biochemical or clinical signs, instead creating a responsive dialogue between the clinician’s interventions and the patient’s immediate physiological state. The cycle begins with a comprehensive baseline assessment, a multisystem sonographic portrait of the patient’s volume status. This is more than a checklist; it is an integrative synthesis. The clinician simultaneously evaluates the IVC for its role as a capacitance vessel, the lung fields for interstitial edema, and the venous Doppler signals for the downstream consequences of elevated central venous pressure. This initial snapshot does more than confirm congestion; it establishes a phenotypic profile. Is this primarily left-sided failure manifesting as pulmonary edema? Or is it a biventricular failure with dominant systemic congestion, evidenced by a grossly abnormal VExUS grade? This phenotypic stratification immediately informs the initial therapeutic prescription, suggesting both the intensity and potential agent of choice—from loop diuretics for acute pulmonary edema to combined diuretic regimens or even vasodilators for severe systemic congestion. Following the initial intervention, the crucial second act is the timely re-assessment. The timing of this follow-up scan is itself a clinical judgment, balancing the urgency of the situation with the pharmacokinetics of the therapy. For an aggressively diuresed patient in the intensive care unit, this may be within 6–12 h. The purpose is not to see complete resolution but to detect a trajectory of change. Are the B-lines beginning to recede from the lung bases? Is the monophasic femoral vein waveform hinting at a return of systolic flow? This is where ultrasound proves its superior sensitivity over traditional metrics like daily weight or urine output, which integrate over longer periods and can be confounded by numerous factors. The ultrasound provides a direct, physiological readout of the therapy’s effect on the target organ—the interstitium and the venous system. Finally, the cycle culminates in the informed titration of therapy. The re-assessment data feed directly back into clinical decision-making, creating a closed-loop system. A significant improvement in all parameters suggests the current strategy is effective and may soon need de-escalation to avoid over-diuresis and pre-renal injury. Conversely, a static or worsening sonographic picture, despite adequate diuretic dosing, is a powerful signal of diuretic resistance or an incorrect initial phenotype. This objective data point mandates an escalation of strategy—perhaps the addition of an adjuvant thiazide diuretic, a transition to continuous infusion, or the consideration of ultrafiltration. This pragmatic workflow transforms the clinician from a passive observer into an active physiological manager. By repeatedly asking the body “how are you responding?” through the lens of ultrasound, we can steer a safer and more effective course through the complex challenge of decongestion, tailoring the therapy to the individual’s real-time needs rather than a pre-ordained protocol.

In conclusion, integrating serial ultrasound assessments into the management of congestive states creates a feedback loop that closes the gap between diagnosis and treatment. It moves fluid management from a reactive to a proactive and guided process, allowing clinicians to objectively tailor decongestive therapy to the individual patient’s physiological response, ultimately aiming to improve both efficacy and safety.

## 4. Discussion

The 2024 ESC Heart Failure guidelines [33] mark a significant shift by formally recognizing splanchnic Doppler ultrasound and lung ultrasound as an adjunct tool for assessing systemic congestion. Traditionally, the evaluation of congestion has relied heavily on clinical examination, weight monitoring, natriuretic peptides, and thoracic imaging. While these parameters remain essential, they are often insufficient to fully capture the complex and compartmentalized nature of venous hypertension in HF. In accordance with PRISMA 2020 standards, the present review systematically synthesizes current evidence on the ultrasonographic assessment of splanchnic, pulmonary and cardiac venous congestion in heart failure, highlighting consistent associations across a diverse body of literature. Conventional and Doppler-based ultrasound techniques emerge as valuable adjunctive tools, complementing standard echocardiography and biomarker evaluation, in line with the 2023 ESC Heart Failure Guidelines [48], which recognize multiparametric ultrasound as an evolving component of congestion assessment.

Our paper represents the first comprehensive review to consolidate the current literature on the use of conventional multiparametric ultrasound for evaluating splanchnic, cardiac, pulmonary vascular alterations in patients with heart failure. The principal finding of this review is that ultrasonographic assessment of the splanchnic circulation provides a valuable, non-invasive window into the degree of systemic venous congestion, offering critical insights into haemodynamic status, prognosis, and potential response to therapy that extend beyond traditional cardiac evaluation. The splanchnic circulation constitutes the body’s largest reservoir for unstressed blood volume (UBV), and its dynamic changes are fundamental to the pathophysiology of heart failure [23,252]. Venous tone regulates the distribution of blood volume between stressed and unstressed compartments, directly influencing pressures in the pulmonary and systemic circulations and contributing to the haemodynamic abnormalities observed in HF [253]. Ultrasound can effectively interrogate this vast reservoir. Techniques such as measuring bowel-wall thickness, particularly in the ascending colon, serve as a direct sonographic correlate of intestinal edema and congestion, which is associated with worse outcomes and may reflect gut barrier dysfunction and systemic inflammation [61,68,79,82]. The grayscale and Doppler evaluation of abdominal veins provide a more direct assessment of elevated right atrial pressure (RAP) and venous congestion. Key findings include hepatic vein monophasicity, portal vein pulsatility (with a pulsatility index < 0.5 indicating significant congestion), reduced portal flow velocity, and in severe cases, hepatofugal flow [93,96,120]. The evidence strongly suggests a linear relationship between RAP and portal vein pulsatility, making it a quantifiable marker for estimating central pressures [75,120]. Importantly, these Doppler abnormalities are not merely observational; they carry significant prognostic weight. The presence of a moderately or severely pulsatile portal vein pattern at discharge is independently associated with increased all-cause mortality, highlighting the role of persistent splanchnic congestion as a marker of high risk [116,117]. The results of this review identified increased portal vein diameter and pulsatility as consistent findings in patients with decompensated heart failure, with the degree of pulsatility showing a linear relationship to RAP. These Doppler features, particularly a pulsatility fraction exceeding 50%, reflect retrograde pressure transmission from the right atrium through the hepatic sinusoids and are predictive of renal dysfunction and mortality [115]. Hepatofugal flow, although less frequent, represents an extreme manifestation of congestion and was observed in patients with severe right heart failure or tricuspid regurgitation. The integration of these portal and hepatic venous indices into echocardiographic assessment not only refines diagnostic precision but also provides prognostic stratification, capturing subclinical haemodynamic burden before overt clinical congestion becomes apparent. The review also reinforces the significance of renal venous Doppler imaging as part of a comprehensive echocardiographic evaluation. Continuous intrarenal venous flow characterizes normal physiology, while biphasic or monophasic patterns indicate elevated renal venous and interstitial pressures. Studies by Iida et al. [56] and corroborated data within the present synthesis demonstrate that monophasic renal venous flow is an independent predictor of mortality, with hazard ratios approaching 17 in advanced heart failure. By linking right-sided haemodynamics with renal perfusion, echocardiography thus provides mechanistic insight into the development of cardiorenal syndrome—a major contributor to morbidity and mortality in this population.

Importantly, splanchnic Doppler ultrasound is associated with haemodynamic congestion and adverse outcomes, though causality cannot be inferred from the available data. Technical limitations remain significant, including restricted acoustic windows, operator dependence, and the influence of patient body habitus on image quality and Doppler accuracy. Future research should focus on prospective validation of these non-invasive indices against invasive right atrial pressure (RAP) measurements and clinically relevant endpoints, such as renal dysfunction or decongestion response, to establish standardized thresholds and strengthen their translational application in heart failure management. By explicitly acknowledging the potential role of splanchnic Doppler ultrasound, the guideline introduces a new dimension: the systematic evaluation of abdominal venous congestion as part of a multiparametric strategy. This acknowledges that venous hypertension in HF often involves a “hidden” splanchnic compartment, which can be evaluated non-invasively through Doppler patterns in abdominal veins. The integration of these Doppler findings into the Venous Excess Ultrasound (VExUS) score represents a major methodological advancement in the bedside assessment of systemic venous congestion [116]. This multiparametric protocol moves beyond flawed, pressure-based metrics like central venous pressure by evaluating the downstream organ-level consequences of elevated RAP. By systematically grading congestion based on IVC diameter and Doppler waveforms in the hepatic, portal, and intrarenal veins, VExUS provides a more holistic and physiologically valid assessment of a patient’s volume status [57,106]. A Comprehensive Review of Current Evidence and Nomenclature is performed by Deschamps et al. [120]. Its clinical utility lies in its superior ability to predict end-organ dysfunction, such as acute kidney injury, and its dynamic nature, as waveforms improve with effective decongestive therapy, offering real-time feedback on therapeutic efficacy [254,255]. The concept of an extended VExUS (eVExUS), incorporating vessels like the internal jugular, femoral, and superior vena cava, further enhances its applicability in challenging clinical scenarios [127,132,135]. It encourages a more holistic, multi-organ view of congestion, paving the way for its potential use in guiding decongestive therapy and improving risk stratification beyond conventional markers. Furthermore, our review underscores that congestion is a multi-organ phenomenon. The strong interplay between lung ultrasound (LUS) and splanchnic circulation findings exemplifies this. B-lines on LUS are not an isolated pulmonary phenomenon but frequently represent the thoracic manifestation of a generalized fluid excess, a significant portion of which is sequestered in the splanchnic bed [209]. Consequently, the successful resolution of B-lines with decongestive therapy signifies not only the clearance of pulmonary edema but also the successful mobilization of fluid from the splanchnic compartment, positioning LUS as a comprehensive haemodynamic monitoring tool [210,237]. The arterial compartment within the splanchnic system also undergoes significant changes. Elevated resistance indices in the hepatic and splenic arteries (e.g., splenic PI > 1.19 in HF patients) reflect increased vascular resistance secondary to congestion and are correlated with right heart filling pressures [114,256]. Similarly, reduced peak systolic velocity in the celiac artery indicates liver hypoperfusion and correlates with a reduced cardiac index, linking splanchnic arterial hypoperfusion to overall cardiac dysfunction [58]. Finally, this review reaffirms that echocardiography remains the cornerstone for the comprehensive evaluation of HF, providing indispensable data on aetiology, phenotype, and haemodynamics [37,257]. However, the assessment must extend beyond the left ventricle. A dedicated evaluation of the right heart and estimation of pulmonary hypertension are critical, as right ventricular dysfunction is a primary driver of splanchnic congestion [191,192].The recent incorporation of intra-abdominal Doppler assessment into international guidelines signifies the growing recognition of its importance in the holistic management of HF patients [178]. From a pathophysiological standpoint, echocardiography uniquely enables simultaneous visualization of the central venous system and its cardiac determinants. The assessment of IVC diameter and collapsibility remains the standard surrogate for RAP, with a dilated, non-collapsible IVC indicating elevated right-sided filling pressures. However, as confirmed by the current synthesis, this measure alone is insufficient to capture the complexity of systemic venous hypertension, which may vary across vascular territories. The inclusion of hepatic and portal vein Doppler analysis markedly enhances the sensitivity of echocardiographic evaluation by extending observation to the downstream consequences of cardiac congestion. The review’s results demonstrate that the transition from a triphasic to a biphasic or monophasic hepatic waveform parallels progressive right atrial pressure elevation and correlates with worsening renal function and mortality, findings echoed in prior investigations by Bouabdallaoui et al. (2020) [67] and Denault et al. (2020) [76]. Overall, the evidence consolidated in this review supports echocardiography as the principal non-invasive modality for evaluating venous congestion in heart failure. Its capacity to integrate central and peripheral haemodynamic information, quantify the systemic consequences of right heart dysfunction, and monitor the response to decongestive therapy makes it indispensable in contemporary management. Beyond its traditional role in assessing ejection fraction and ventricular morphology, echocardiography now serves as a comprehensive haemodynamic monitor linking cardiac dysfunction to organ-level consequences. When interpreted through frameworks such as VExUS, it enables a holistic, multi-organ perspective that aligns with current European and American guideline recommendations advocating multiparametric ultrasound for congestion evaluation [180]. Future work should focus on prospective validation of echocardiographic congestion scores, standardization of Doppler thresholds, and integration with emerging biomarkers and machine learning algorithms to optimize precision in heart failure care.

Finally, several limitations must be outlined. The current evidence base sup-porting venous Doppler ultrasound and the Venous Excess Ultrasound Score (VExUS) in heart failure (HF) remains constrained by methodological and technical challenges that should temper the interpretation of its clinical implications. A major limitation is the heterogeneity among available studies. Early validation of the VExUS framework was performed primarily in postoperative cardiac or critically ill cohorts, whereas more recent studies have expanded to ambulatory and acute decompensated HF populations [63,258]. These cohorts differ substantially in disease severity, haemodynamic profiles, and ultrasound acquisition techniques, including the use of ECG gating, Doppler sample volume placement, and threshold definitions for pulsatility and collapsibility indices. Such methodological variability reduces comparability and complicates meta-analytic synthesis, underscoring the need for standardized imaging protocols and consistent reporting. Another limitation is the absence of randomized controlled trials assessing whether ultrasound-guided management strategies improve outcomes beyond conventional clinical assessment. Although several observational studies demonstrate associations between higher VExUS grades and adverse events—including acute kidney injury, prolonged hospitalization, and mortality—causal evidence remains lacking. The prospective mul-ticentre study by Torres-Arrese et al. (2023) [258] confirmed the prognostic value of VExUS in HF, but randomized data demonstrating that VExUS-guided interventions alter patient trajectories are not yet available. Consequently, current international guidelines advocate the use of ultrasound as a complementary tool for congestion assessment rather than as an independent determinant of therapy [180]. Operator dependency represents another important limitation. The multiparametric nature of VExUS requires expertise in aligning Doppler angles and interpreting wave-form morphology across several venous districts. Although reproducibility is generally good among trained users, inter-observer variability increases among novices [259] highlighting the need for structured education and credentialing programs. Finally, technical constraints—particularly in obese or mechanically ventilated patients can impede image acquisition and distort venous waveforms [117]. Alternative acoustic windows, ECG gating, and cautious interpretation may mitigate but not eliminate these challenges. Addressing these limitations through methodological standardization, operator training, and interventional research will be essential for in-tegrating VExUS reliably into heart failure management.

Our findings and the resulting integrated ultrasound protocol must be contextualized within the evolving landscape of heart failure management, particularly in relation to the 2023 ESC Heart Failure guidelines [33,48]. While these guidelines represent a significant advancement by formally recognizing the role of lung ultrasound for pulmonary congestion and acknowledging the emerging utility of intra-abdominal Doppler, their recommendations largely present these modalities as complementary yet distinct tools. In contrast, our systematic review synthesizes evidence to propose a paradigm of multiparametric, multi-organ sonographic assessment. We demonstrate that venous congestion is not a compartmentalized phenomenon but a systemic state, where alterations in splanchnic, renal, pulmonary, and cardiac haemodynamics are intrinsically linked. Our proposed protocol, which logically progresses from IVC screening to comprehensive VExUS grading and incorporates an “escape valve” through extended venous assessment (eVExUS), provides a clinically actionable algorithm that the current guidelines lack. This approach moves beyond the ESC’s recognition of individual techniques by offering a unified framework that captures the full haemodynamic burden of congestion. By integrating these disparate sonographic windows into a single, interpretable score, our protocol expands upon the guideline recommendations to offer a more holistic and dynamic tool for phenotyping patients, which may ultimately enable more precise and personalized decongestive therapy tailored to the patient’s systemic congestion profile rather than isolated clinical or echocardiographic signs.

The main limitation now is that the integration of comprehensive ultrasound assessment, particularly venous Doppler protocols like VExUS, into the haemodynamic toolkit marks a significant advance in our ability to phenotype congestion at the bedside. However, this evolving paradigm presents a new frontier of scientific and clinical questions that must be addressed to solidify its role in precision medicine. The promising correlations and prognostic associations established in initial studies now demand validation and refinement through targeted research programs. A primary and immediate challenge lies in the standardization and phenotypic stratification of diagnostic cut-offs. The current application of venous Doppler often utilizes universal thresholds, yet it is physiologically plausible that the clinical implications of a given VExUS grade or portal vein pulsatility may differ substantially across distinct heart failure phenotypes. The pathophysiological milieu of Heart Failure with preserved Ejection Fraction (HFpEF), characterized by left atrial hypertension and ventricular stiffness, likely imposes a different dynamic on the venous system compared to the low-output state of advanced HF with reduced EF (HFrEF). Future large-scale, prospective cohort studies must, therefore, move beyond validation and strive to establish phenotype-specific, and perhaps even etiology-specific, normative values and prognostic thresholds. This stratification is essential to transform a general marker of congestion into a precise tool for individual risk assessment. intrFurthermore, the compelling observational data linking venous congestion to adverse outcomes sets the stage for the most critical next step: definitive interventional trials. The fundamental question remains whether knowledge of the venous Doppler profile can actively guide therapy to improve hard endpoints. There is an urgent need for multicentre, randomized controlled trials comparing a protocolized, VExUS-guided decongestive strategy against standard, care-as-usual management. Such trials should investigate whether steering diuretic and vasodilator therapy based on serial venous Doppler assessment can effectively mitigate the cardiorenal syndrome, reduce rates of rehospitalization, and ultimately improve patient survival. Demonstrating a causal link between ultrasound-guided management and tangible clinical benefit is the necessary evidence to catalyse a paradigm shift from reactive to physiologically informed, pre-emptive decongestion.

## 5. Conclusions

This comprehensive review synthesizes current evidence demonstrating that conventional ultrasound techniques provide a valuable, non-invasive window into systemic venous congestion in heart failure patients. The analysis reveals that alterations in the splanchnic circulation—including portal vein dilation and pulsatility, hepatic vein waveform changes, and bowel wall thickening—are consistently associated with the severity of heart failure, elevated right atrial pressure, and adverse clinical outcomes. The integration of these parameters into multiparametric frameworks, such as the Venous Excess Ultrasound (VExUS) score, significantly enhances the detection and grading of congestion beyond traditional cardiac assessment. By offering real-time, organ-level insights, splanchnic ultrasound complements standard echocardiography and biomarker evaluation, facilitating improved risk stratification and guiding decongestive therapy. The findings underscore a paradigm shift towards a holistic, multi-organ assessment of congestion, positioning ultrasound as an indispensable tool for optimizing personalized management in both acute and chronic heart failure. Further prospective validation is warranted to standardize measurements and solidify its role in routine clinical practice.

## Figures and Tables

**Table 1:** Characteristics of Included Studies.

Study Feature	Description (Number of Studies or Range)	Representative Examples (Citations)
**Total Studies/Patients**	148 studies/~7000 patients	
**Publication Years**	1986–2025	Moriyasu et al., 1986 [54]; Saadi et al., 2025 [46]
**Study Designs**	• Prospective Cohort: **e.g., [55]** studies • Retrospective Cohort: **e.g., [44]** studies • Cross-Sectional: **e.g., [30]** studies • Case-Control: **e.g., [5]** studies • Systematic Reviews: **e.g., [3]** studies	• Prospective: Iida et al., 2016 [56], Beaubien-Souligny et al., 2018 [57] • Retrospective: Kuwahara et al., 2023 [58]
**Patient Populations**	• Acute Decompensated HF: **e.g., [59]** studies • Chronic Ambulatory HF: **e.g., [38]** studies • Post-Cardiac Surgery/Critically Ill: **e.g., [25]** studies • Mixed/Unspecified HF: **e.g., [13]** studies	• Acute: Pivetta et al., 2015 [60], Ikeda et al., 2017 [61] • Chronic: Platz et al., 2016 [62] • Post-Surgical: Beaubien-Souligny et al., 2020 [63]
**HF Phenotypes**	• HFrEF: **e.g., [64]** studies • HFpEF: **e.g., [25]** studies • Mixed/All Phenotypes: **e.g., [65]** studies	• HFpEF: Yoshihisa et al., 2020 [66] • Mixed: Bouabdallaoui et al., 2020 [67]
**Ultrasdomatic Modalities Used**	• Splanchnic B-mode/Doppler (isolated): **e.g., [68]** studies • VExUS Protocol: **e.g., [15]** studies • Echocardiography (for congestion): **e.g., [49]** studies • Lung Ultrasound (LUS): **e.g., [38] **studies • Multi-organ POCUS Protocol: **e.g., [20]** studies	• Splanchnic: Goncalvesova et al., 2010 [69] • VExUS: Bhardwaj et al., 2020 [70] • LUS: Gargani et al., 2015 [71]
**Primary Outcomes Measured**	• Correlation with Invasive Haemodynamics: **e.g., [30]** studies • Prognosis (Mortality/Hospitalization): **e.g., [72]** studies • Acute Kidney Injury (AKI): **e.g., [20]** studies • Diuretic Response/Therapy Guidance: **e.g., [15]** studies • Technical Feasibility/Diagnostic Accuracy: **e.g., [38]** studies	• Haemodynamics: Rengo et al., 1998 [73] • Prognosis: Ciccone et al., 2014 [74] • AKI: Beaubien-Souligny et al., 2018 [57]
**Sample Size (Range)**	Small (<100 patients): **e.g., [75]** studies Medium (100–500 patients): **e.g., [49]** studies Large (>500 patients): **e.g., [8]** studies	• Small: Denault et al., 2020 [76] case series • Large: Pivetta et al., 2015 [60] (*n* = 1005)

**Table 2:** Main grayscale US findings of splanchnic veins congestion in heart failure and hepatopathies.

	Hepatic Veins	Portal Venous System	
Normal	IVC collapsibility >50% IVC diameter ≤ 21 mm IV diameter ≤ 10 mm	PV diameter < 13 mm Preserved collapsibility	
			Other
Congestive hepatopathy	Increased diameter Reduced collapsibility <50%	Increased vessels diameter (PV > 13 mm) Reduced collapsibility	Hepatomegaly Ascites Splenomegaly Porto-systemic collaterals
Liver cirrhosis	Reduced diameter Serpiginous aspect	**Signs of Portale Hypertension**	

**Table 3:** Spectral variations in hepatic vessels.

	Intrahepatic Veins	Portal Vein	Hepatic Artery
**Normal**	Triphasic pattern: “a” wave: positive, “a” > “v” “S” wave: negative, “S” > “D” “v” wave: positive; “v” < “a” “D” wave: negative, “D” < “S”	Anterograde flow Smoothly undulating venous waveforms Systolic speed 16–40 cm/s Pulsatility index > 0.5	Maximum systolic speed: 30–60 cm/s Resistive index 0.55–0.7
**Heart failure**	Increased anterograde and retrograde speeds	***All cases of hepatic congestion***	
	Increased pulsatility		
**Right heart**	Higher “a” and “v” waves		
**failure**	Adequate ratio maintained between S and D waves	Pulsatility index < 0.5	Resistive index > 0.7
	High and both positive “a” and “v” waves		Reduced systolic speed
**Tricuspid regurgitation**	Reduced “S” wave “S” wave < “D” wave		*(more common in LC)*
	Severe TR: “S” wave retrograde (“a-S-v complex”)		
**Liver cirrhosis**	Loss of triphasic pattern	Systolic speed < 12.8 cm/s till reversal flow and thrombosis	

**Table 4:** Comparison of Alternative Venous Sites for the Assessment of Systemic Congestion (eVExUS). This summary table provides a concise, clinically focused comparison of the primary alternative venous sites used for Doppler assessment in the Venous Excess Ultrasound (VExUS) protocol. When acquisition of the standard hepatic vein is challenging, these sites offer viable pathways to diagnose systemic venous congestion. The table synthesizes key practical parameters—including ease of acquisition, specific advantages, and important limitations—to guide clinicians in selecting the most appropriate site based on patient factors and clinical context. It is designed to serve as a rapid reference to enhance the utility and application of VExUS at the point of care. Clinical Utility and Selection Guide: For a rapid, qualitative assessment: The Internal Jugular Vein is a useful screening tool. The presence of a monophasic waveform is a strong, quick indicator of systemic congestion. For a quantitative, IVC-surrogate assessment: The Femoral Vein is the most practical and validated alternative for formal VExUS grading when the hepatic vein is unavailable. Its waveform is familiar and can be directly integrated into the existing VExUS algorithm. For specific, complex cases: The SVC may be interrogated by experienced sonographers when abdominal views are impossible and central pressure confirmation is critical, but it is not recommended for routine use.

Venous Site	Ease of Acquisition	Key Advantages	Key Limitations & Pitfalls	Key Doppler Findings in Congestion
**Internal Jugular (IJ) Vein**	**Moderate to High** • Superficial, easily visualized with linear probe. • Patient positioning (Trendelenburg) can facilitate.	• **Minimal respiratory variation.** Less influenced by intra-abdominal pressure (IAP) than portal/hepatic veins. • **Rapidly responsive** to haemodynamic changes. • Useful for **trending** over time.	• **Highly sensitive to transducer pressure.** Can easily occlude the vein. • Can be difficult in patients with short necks, obesity, or cervical collars. • **Not validated for formal VExUS grading.** Best used as a qualitative “congestion marker.”	• **Loss of phasicity:** The normal, pulsatile waveform becomes monophasic or severely blunted. • **Continuous, high-velocity flow** indicates significantly elevated right atrial pressure.
**Femoral Vein**	**High** • Large, superficial, and easy to locate with a linear probe. • Consistent anatomical location.	• **Excellent surrogate for IVC/right atrial pressure.** • Waveform closely mirrors the hepatic vein pattern. • **Well-suited for formal VExUS grading** (can be classified as Grade 0–3). • Less affected by ascites or liver morphology.	• **Highly influenced by IAP.** A tense abdomen or ascites can artificially alter the waveform. • Can be affected by **iliac vein thrombosis** or compression. • **Influenced by cirrhosis:** Portal hypertension can cause pre-sinusoidal resistance, potentially altering waveforms.	• **Progressive blunting of systolic wave (S).** • **S < D (Diastolic) flow.** • **Systolic Flow Reversal** (severe congestion, VExUS Grade 3).
**Superior Vena Cava (SVC)/Brachiocephalic Vein**	**Low** • Requires a low-frequency phased array or curvilinear probe with a suprasternal or supraclavicular view. • Technically challenging and operator-dependent.	• **Directly pre-cardiac.** Provides the most direct assessment of central venous pressure short of a catheter. • **Unaffected by IAP or liver disease.**	• **Acquisition is difficult and often not feasible** in unselected patients (e.g., COPD, obesity, mechanical ventilation). • **Not a practical routine site.** • Limited data on its use in VExUS protocols.	• **Severe blunting of the systolic wave.** • **Prominent diastolic wave.** • **Systolic Flow Reversal** indicates profound congestion.

Note: The presence of conditions like severe tricuspid regurgitation will profoundly affect all systemic venous Doppler waveforms, typically causing prominent systolic reversal.

**Table 5:** Comparison of main cardiac characteristics in heart failure with preserved ejection fraction (HFpEF), heart failure with reduced ejection fraction (HFrEF), and pulmonary hypertension (PH).

Feature	HFpEF	HFrEF	Pulmonary Hypertension
**Primary dysfunction**	Diastolic impairment (impaired relaxation, stiff ventricle)	Systolic impairment (reduced contractility, LVEF ≤ 40%)	Increased RV afterload due to elevated pulmonary vascular resistance
**Structural remodeling**	Concentric LV hypertrophy, LA enlargement, interstitial fibrosis	Eccentric LV hypertrophy, dilatation, secondary MR	RV hypertrophy (early), RV dilatation (late), septal shift
**Metabolic features**	Impaired substrate flexibility, reduced ketone oxidation [1,2]	Reverse remodelling under SGLT2i/ARNI therapy [5,6]	RV microvascular remodelling (shorter, tortuous vessels, preserved density) [8]
**Microvascular/Perfusion**	Reduced myocardial perfusion reserve, LA functional changes [3]	Partially reversible LV microvascular dysfunction	RV perfusion and microarchitecture linked to coupling [9]
**Neurohormonal/Inflammatory role**	Inflammatory mediators (IL-1R1, fibrosis pathways) [4]	Strong neurohormonal activation (RAAS, sympathetic system)	Endothelial dysfunction, smooth muscle proliferation, in situ thrombosis
**Imaging markers**	Strain analysis shows impaired LA reservoir and LV diastolic strain [3]	Global longitudinal strain improves with therapy [6]	RV strain by MRI/echo, CT-MRI integration improves PH detection [10,11]
**Reversibility**	Limited, comorbidity-driven phenotype	Partially reversible with optimal therapy [7]	Some vascular and RV changes reversible after unloading [8]

**Table 6:** Advantages and limitations of POCUS methods for clinical congestion assessment.

POCUS Application	Clinical Relevance	Advantages	Limitations
IVC ultrasound	Provides an estimation of	Relatively easy to perform; able to use	Unreliable in many clinical scenarios
	RAP	handheld ultrasound devices	(e.g., mechanical ventilation,
			pulmonary embolism, PH, cardiac
			tamponade, intra-abdominal
			hypertension, chronic TR, athletes);
			unable to distinguish between
			hypovolemia, euvolemia, and high-
			output cardiac states; collapsibility
			influenced by strength of breath
Internal jugular vein	Provides an estimation of	Relatively easy to perform; able to use	Operator variability (bed angle,
ultrasound	RAP	handheld ultrasound devices; useful	transducer pressure, off-axis views);
		when IVC is inaccessible or unreliable	protocol variability (e.g., column
		(e.g., cirrhosis, obesity)	height, change with Valsalva,
			respiratory variation); incorrect
			assumptions (e.g., RA depth is 5.0 cm)
Hepatic vein Doppler	Aids in the assessment of	Same window used for assessing the	Need ECG tracing; unreliable in atrial
	systemic venous	IVC; supplemental information (e.g.,	fibrillation, right ventricular systolic
	congestion	right ventricular systolic function,	dysfunction, chronic PH, TR, cirrhosis
		constriction and tamponade); exhibits	
		dynamic change in response to	
		decongestive	
		treatment	
Portal vein Doppler	Aids in the assessment of	Do not need ECG; exhibits dynamic	Operator variability (Doppler sampling
	systemic venous	change in response to decongestive	location); unreliable in athletes (e.g.,
	congestion	treatment (pulsatility may	pulsatility without high RAP) and
		improve even in chronic TR)	cirrhosis (e.g., no pulsatility with high
			RAP or pulsatility due to arterioportal
			shunts)
Intrarenal venous	Aids in the assessment of	Simultaneous arterial Doppler allows	Technically challenging (especially when
Doppler	systemic venous	identification of cardiac cycle; exhibits	patients unable to hold breath);
	congestion	dynamic change in response to	operator variability (e.g., misinterpret
		decongestive treatment	pulsatility of main renal vessel as renal
			parenchymal vessel); change in
			response to decongestive treatment
			may be delayed in the presence of
			interstitial edema; no available data for
			patients with advanced chronic kidney
			disease
Femoral Vein Doppler (FVD)	Aids in the assessment of	Relatively easy to perform; feasible in	Operator variability (misaligned Doppler
	systemic venous	patients unable to hold their breath	tracings, overreliance on absolute
	congestion		velocities or percent pulsatility); unable
			to rule out venous congestion;
			individual variability (cyclical variation
			limits use of the stasis index)
Superior vena cava	Aids in the assessment of	Useful when hepatic or renal vessels are	Need ECG tracing; technically
Doppler	systemic venous	inaccessible or unreliable (e.g.,	challenging transthoracic windows
	congestion	cirrhosis, advanced kidney disease)	(especially in obese individuals)
Lung ultrasound	Provides an assessment of	Relatively easy to perform; able to use	Operator variability (transducer angle);
	extravascular lung water	handheld ultrasound devices; may	technically challenging in obese
	(e.g., pulmonary edema,	reduce need for serial chest x-ray to	individuals; protocol variability; B lines
	pleural effusions)	monitor response to decongestive	lack specificity for pulmonary edema;
		treatment	unreliable in preexisting lung disease
Mitral E/A ratio and E/e′	Provides an estimation of	Reproducible; prognostic; useful to	Unreliable in many clinical scenarios
ratio	left atrial pressure	distinguish cardiogenic versus	(e.g., atrial fibrillation; mitral annular
		noncardiogenic pulmonary edema	calcification; mitral valve and
			pericardial disease); operator
			variability
(Doppler cursor angle; sample volume placement); indeterminate E/e′ values are common			

## Data Availability

No new data were created or analysed in this study. Data sharing is not applicable to this article as it is a systematic review of existing published literature.

## Supplementary Materials

The following supporting information can be downloaded at: https://www.mdpi.com/article/10.3390/jcm14228147/s1, File S1: PRISMA 2020 checklist, File S2: PRISMA 2020 abstract checklist.

## References

[1] Chen-Izu Y., Banyasz T., Shaw J.A., Izu L.T. (2024). The Heart is a Smart Pump: Mechanotransduction Mechanisms of the Frank-Starling Law and the Anrep Effect. Annu. Rev. Physiol..

[2] Swenne C.A., Shusterman V. (2024). Neurocardiology: Major mechanisms and effects. J. Electrocardiol..

[3] Manolis A.A., Manolis T.A., Manolis A.S. (2023). Neurohumoral Activation in Heart Failure. Int. J. Mol. Sci..

[4] Patterson S.W., Piper H., Starling E.H. (1914). The regulation of the heart beat. J. Physiol..

[5] Nichols C.G., Hanck D.A., Jewell B.R. (1988). The Anrep effect: An intrinsic myocardial mechanism. Can. J. Physiol. Pharmacol..

[6] Hornby-Foster I., Richards C.T., Drane A.L., Lodge F.M., Stembridge M., Lord R.N., Davey H., Yousef Z., Pugh C.J.A. (2024). Resistance- and endurance-trained young men display comparable carotid artery strain parameters that are superior to untrained men. Eur. J. Appl. Physiol..

[7] Herring N., Paterson D., Herring N., Paterson D. (2018). Haemodynamics: Flow, pressure and resistance. Levick’s Introduction to Cardiovascular Physiology.

[8] O’Rourke M.F., Hashimoto J. (2007). Mechanical factors in arterial aging: A clinical perspective. J. Am. Coll. Cardiol..

[9] Laurent S., Cockcroft J., Van Bortel L., Boutouyrie P., Giannattasio C., Hayoz D., Pannier B., Vlachopoulos C., Wilkinson I., Struijker-Boudier H. (2006). Expert consensus document on arterial stiffness: Methodological issues and clinical applications. Eur. Heart J..

[10] Vanhoutte P.M., Shimokawa H., Tang E.H., Feletou M. (2009). Endothelial dysfunction and vascular disease. Acta Physiol..

[11] Schiffrin E.L. (2012). Vascular remodeling in hypertension: Mechanisms and treatment. Hypertension.

[12] Shirwany N.A., Zou M.H. (2010). Arterial stiffness: A brief review. Acta Pharmacol. Sin..

[13] Mitchell G.F. (2014). Arterial stiffness and hypertension. Hypertension.

[14] Guyton A.C., Polizo D., Armstrong G.G. (1954). Mean circulatory filling pressure measured immediately after cessation of heart pumping. Am. J. Physiol..

[15] Versprille A., Jansen J.R. (1985). Mean systemic filling pressure as a characteristic pressure for venous return. Pflugers Arch..

[16] Guyton A.C., Lindsey A.W., Abernathy B., Richardson T. (1957). Venous return at various right atrial pressures and the normal venous return curve. Am. J. Physiol..

[17] Aya H.D., Cecconi M. (2016). Mean Systemic Filling Pressure Is an Old Concept but a New Tool for Fluid Management. Perioperative Fluid Management.

[18] Spiegel R. (2016). Stressed vs. unstressed volume and its relevance to critical care practitioners. Clin. Exp. Emerg. Med..

[19] Rosenblum J.D., Boyle C.M., Schwartz L.B. (1997). The mesenteric circulation. Anatomy and physiology. Surg. Clin. N. Am..

[20] Netter F.H. (2025). Overview of lower digestive tract. Netter Collection of Medical Illustrations: Digestive System, Part II—Lower Digestive Tract.

[21] Yaku H., Fudim M., Shah S.J. (2024). Role of splanchnic circulation in the pathogenesis of heart failure: State-of-the-art review. J. Cardiol..

[22] Rutlen D.L., Supple E.W., Powell W.J. (1979). The role of the liver in the adrenergic regulation of blood flow from the splanchnic to the central circulation. Yale J. Biol. Med..

[23] Parks D.A., Jacobson E.D. (1985). Physiology of the splanchnic circulation. Arch. Intern. Med..

[24] Mocan D., Lala R.I., Puschita M., Pilat L., Darabantiu D.A., Pop Moldovan A. (2024). The congestion “pandemic” in acute heart failure patients. Biomedicines.

[25] Miller W.L. (2024). Congestion/decongestion in heart failure: What does it mean, how do we assess it, and what are we missing?-is there utility in measuring volume?. Heart Fail. Rev..

[26] Rubio-Gracia J., Demissei B.G., Ter Maaten J.M., Cleland J.G., O’Connor C.M., Metra M., Ponikowski P., Teerlink J.R., Cotter G., Davison B.A. (2018). Prevalence, predictors and clinical outcome of residual congestion in acute decompensated heart failure. Int. J. Cardiol..

[27] Lala A., McNulty S.E., Mentz R.J., Dunlay S.M., Vader J.M., AbouEzzeddine O.F., DeVore A.D., Khazanie P., Redfield M.M., Goldsmith S.R. (2015). Relief and Recurrence of Congestion During and After Hospitalization for Acute Heart Failure: Insights From Diuretic Optimization Strategy Evaluation in Acute Decompensated Heart Failure (DOSE-AHF) and Cardiorenal Rescue Study in Acute Decompensated Heart Failure (CARESS-HF). Circ. Heart Fail..

[28] Drazner M.H., Hellkamp A.S., Leier C.V., Shah M.R., Miller L.W., Russell S.D., Young J.B., Califf R.M., Nohria A. (2008). Value of clinician assessment of hemodynamics in advanced heart failure: The ESCAPE trial. Circ. Heart Fail..

[29] Narang N., Chung B., Nguyen A., Kalathiya R.J., Laffin L.J., Holzhauser L., Ebong I.A., Besser S.A., Imamura T., Smith B.A. (2020). Discordance Between Clinical Assessment and Invasive Hemodynamics in Patients With Advanced Heart Failure. J. Card. Fail..

[30] Benjamin E.J., Blaha M.J., Chiuve S.E., Cushman M., Das S.R., Deo R., de Ferranti S.D., Floyd J., Fornage M., Gillespie C. (2017). Heart Disease and Stroke Statistics-2017 Update: A Report From the American Heart Association. Circulation.

[31] Ostrominski J.W., DeFilippis E.M., Bansal K., Riello R.J., Bozkurt B., Heidenreich P.A., Vaduganathan M. (2024). Contemporary American and European Guidelines for Heart Failure Management: JACC: Heart Failure Guideline Comparison. JACC Heart Fail..

[32] Bozkurt B., Coats A.J.S., Tsutsui H., Abdelhamid C.M., Adamopoulos S., Albert N., Anker S.D., Atherton J., Bohm M., Butler J. (2021). Universal definition and classification of heart failure: A report of the Heart Failure Society of America, Heart Failure Association of the European Society of Cardiology, Japanese Heart Failure Society and Writing Committee of the Universal Definition of Heart Failure: Endorsed by the Canadian Heart Failure Society, Heart Failure Association of India, Cardiac Society of Australia and New Zealand, and Chinese Heart Failure Association. Eur. J. Heart Fail..

[33] McDonagh T.A., Metra M., Adamo M., Gardner R.S., Baumbach A., Bohm M., Burri H., Butler J., Celutkiene J., Chioncel O. (2024). 2023 Focused Update of the 2021 ESC Guidelines for the diagnosis and treatment of acute and chronic heart failure: Developed by the task force for the diagnosis and treatment of acute and chronic heart failure of the European Society of Cardiology (ESC) With the special contribution of the Heart Failure Association (HFA) of the ESC. Eur. J. Heart Fail..

[34] Zhang J., Xu M., Chen T., Zhou Y. (2021). Correlation Between Liver Stiffness and Diastolic Function, Left Ventricular Hypertrophy, and Right Cardiac Function in Patients With Ejection Fraction Preserved Heart Failure. Front. Cardiovasc. Med..

[35] Samsky M.D., Patel C.B., DeWald T.A., Smith A.D., Felker G.M., Rogers J.G., Hernandez A.F. (2013). Cardiohepatic interactions in heart failure: An overview and clinical implications. J. Am. Coll. Cardiol..

[36] Pieske B., Tschope C., de Boer R.A., Fraser A.G., Anker S.D., Donal E., Edelmann F., Fu M., Guazzi M., Lam C.S.P. (2020). How to diagnose heart failure with preserved ejection fraction: The HFA-PEFF diagnostic algorithm: A consensus recommendation from the Heart Failure Association (HFA) of the European Society of Cardiology (ESC). Eur. J. Heart Fail..

[37] Heidenreich P.A., Bozkurt B., Aguilar D., Allen L.A., Byun J.J., Colvin M.M., Deswal A., Drazner M.H., Dunlay S.M., Evers L.R. (2022). 2022 AHA/ACC/HFSA Guideline for the Management of Heart Failure: A Report of the American College of Cardiology/American Heart Association Joint Committee on Clinical Practice Guidelines. J. Am. Coll. Cardiol..

[38] Anker S.D., Butler J., Filippatos G., Ferreira J.P., Bocchi E., Bohm M., Brunner-La Rocca H.P., Choi D.J., Chopra V., Chuquiure-Valenzuela E. (2021). Empagliflozin in Heart Failure with a Preserved Ejection Fraction. N. Engl. J. Med..

[39] Solomon S.D., McMurray J.J.V., Claggett B., de Boer R.A., DeMets D., Hernandez A.F., Inzucchi S.E., Kosiborod M.N., Lam C.S.P., Martinez F. (2022). Dapagliflozin in Heart Failure with Mildly Reduced or Preserved Ejection Fraction. N. Engl. J. Med..

[40] Schwinger R.H.G. (2021). Pathophysiology of heart failure. Cardiovasc. Diagn. Ther..

[41] Tanai E., Frantz S. (2015). Pathophysiology of Heart Failure. Compr. Physiol..

[42] Fudim M., Neuzil P., Malek F., Engelman Z.J., Reddy V.Y. (2021). Greater Splanchnic Nerve Stimulation in Heart Failure With Preserved Ejection Fraction. J. Am. Coll. Cardiol..

[43] Yancy C.W., Jessup M., Bozkurt B., Butler J., Casey D.E., Drazner M.H., Fonarow G.C., Geraci S.A., Horwich T., Januzzi J.L. (2013). 2013 ACCF/AHA guideline for the management of heart failure: Executive summary: A report of the American College of Cardiology Foundation/American Heart Association Task Force on practice guidelines. Circulation.

[44] Greene S.J., Bauersachs J., Brugts J.J., Ezekowitz J.A., Lam C.S.P., Lund L.H., Ponikowski P., Voors A.A., Zannad F., Zieroth S. (2023). Worsening Heart Failure: Nomenclature, Epidemiology, and Future Directions: JACC Review Topic of the Week. J. Am. Coll. Cardiol..

[45] Lozano-Jimenez S., Garcia Sebastian C., Vela Martin P., Garcia Magallon B., Martin Centellas A., de Castro D., Mitroi C., Del Prado Diaz S., Hernandez-Perez F.J., Jimenez-Blanco Bravo M. (2025). Prevalence and prognostic impact of subclinical venous congestion in patients hospitalized for acute heart failure. Eur. Heart J. Acute Cardiovasc. Care.

[46] Saadi M.P., Silvano G.P., Machado G.P., Almeida R.F., Scolari F.L., Biolo A., Aboumarie H.S., Telo G.H., Donelli da Silveira A. (2025). Modified VExUS: A Dynamic Tool to Predict Mortality in Acute Decompensated Heart Failure. J. Am. Soc. Echocardiogr..

[47] Gamarra A., Salamanca J., Diez-Villanueva P., Cuenca S., Vazquez J., Aguilar R.J., Diego G., Rodriguez A.P., Alfonso F. (2025). Ultrasound imaging of congestion in heart failure: A narrative review. Cardiovasc. Diagn. Ther..

[48] McDonagh T.A., Metra M., Adamo M., Gardner R.S., Baumbach A., Bohm M., Burri H., Butler J., Celutkiene J., Chioncel O. (2023). 2023 Focused Update of the 2021 ESC Guidelines for the diagnosis and treatment of acute and chronic heart failure. Eur. Heart J..

[49] Page M.J., McKenzie J.E., Bossuyt P.M., Boutron I., Hoffmann T.C., Mulrow C.D., Shamseer L., Tetzlaff J.M., Akl E.A., Brennan S.E. (2021). The PRISMA 2020 statement: An updated guideline for reporting systematic reviews. BMJ.

[50] Wells A.U., Shea B., O’Connell D., Peterson J., Welch V.A., Losos M., Tugwell P. The Newcastle-Ottawa Scale (NOS) for assessing the quality of nonrandomised studies in meta-analyses. https://www.ohri.ca/programs/clinical_epidemiology/oxford.asp.

[51] Sterne J.A.C., Savovic J., Page M.J., Elbers R.G., Blencowe N.S., Boutron I., Cates C.J., Cheng H.Y., Corbett M.S., Eldridge S.M. (2019). RoB 2: A revised tool for assessing risk of bias in randomised trials. BMJ.

[52] Fede G., Privitera G., Tomaselli T., Spadaro L., Purrello F. (2015). Cardiovascular dysfunction in patients with liver cirrhosis. Ann. Gastroenterol..

[53] Mukhtar A., Dabbous H. (2016). Modulation of splanchnic circulation: Role in perioperative management of liver transplant patients. World J. Gastroenterol..

[54] Moriyasu F., Nishida O., Ban N., Nakamura T., Sakai M., Miyake T., Uchino H. (1986). “Congestion index” of the portal vein. AJR Am. J. Roentgenol..

[55] Wells M.L., Venkatesh S.K. (2018). Congestive hepatopathy. Abdom. Radiol..

[56] Iida N., Seo Y., Sai S., Machino-Ohtsuka T., Yamamoto M., Ishizu T., Kawakami Y., Aonuma K. (2016). Clinical Implications of Intrarenal Hemodynamic Evaluation by Doppler Ultrasonography in Heart Failure. JACC Heart Fail..

[57] Beaubien-Souligny W., Benkreira A., Robillard P., Bouabdallaoui N., Chasse M., Desjardins G., Lamarche Y., White M., Bouchard J., Denault A. (2018). Alterations in Portal Vein Flow and Intrarenal Venous Flow Are Associated With Acute Kidney Injury After Cardiac Surgery: A Prospective Observational Cohort Study. J. Am. Heart Assoc..

[58] Kuwahara N., Honjo T., Sone N., Imanishi J., Nakayama K., Kamemura K., Iwahashi M., Ohta S., Kaihotsu K. (2023). Clinical impact of portal vein pulsatility on the prognosis of hospitalized patients with acute heart failure. World J. Cardiol..

[59] McNaughton D.A., Abu-Yousef M.M. (2011). Doppler US of the liver made simple. Radiographics.

[60] Pivetta E., Goffi A., Lupia E., Tizzani M., Porrino G., Ferreri E., Volpicelli G., Balzaretti P., Banderali A., Iacobucci A. (2015). Lung Ultrasound-Implemented Diagnosis of Acute Decompensated Heart Failure in the ED: A SIMEU Multicenter Study. Chest.

[61] Ikeda Y., Ishii S., Fujita T., Iida Y., Kaida T., Nabeta T., Maekawa E., Yanagisawa T., Koitabashi T., Takeuchi I. (2017). Prognostic impact of intestinal wall thickening in hospitalized patients with heart failure. Int. J. Cardiol..

[62] Platz E., Lewis E.F., Uno H., Peck J., Pivetta E., Merz A.A., Hempel D., Wilson C., Frasure S.E., Jhund P.S. (2016). Detection and prognostic value of pulmonary congestion by lung ultrasound in ambulatory heart failure patients. Eur. Heart J..

[63] Beaubien-Souligny W., Rola P., Haycock K., Bouchard J., Lamarche Y., Spiegel R., Denault A.Y. (2020). Quantifying systemic congestion with Point-Of-Care ultrasound: Development of the venous excess ultrasound grading system. Ultrasound J..

[64] Galie N., Humbert M., Vachiery J.L., Gibbs S., Lang I., Torbicki A., Simonneau G., Peacock A., Vonk Noordegraaf A., Beghetti M. (2016). 2015 ESC/ERS Guidelines for the diagnosis and treatment of pulmonary hypertension: The Joint Task Force for the Diagnosis and Treatment of Pulmonary Hypertension of the European Society of Cardiology (ESC) and the European Respiratory Society (ERS): Endorsed by: Association for European Paediatric and Congenital Cardiology (AEPC), International Society for Heart and Lung Transplantation (ISHLT). Eur. Heart J..

[65] Prowle J.R., Bellomo R. (2013). Fluid administration and the kidney. Curr. Opin. Crit. Care.

[66] Yoshihisa A., Ishibashi S., Matsuda M., Yamadera Y., Ichijo Y., Sato Y., Yokokawa T., Misaka T., Oikawa M., Kobayashi A. (2020). Clinical Implications of Hepatic Hemodynamic Evaluation by Abdominal Ultrasonographic Imaging in Patients With Heart Failure. J. Am. Heart Assoc..

[67] Bouabdallaoui N., Beaubien-Souligny W., Oussaid E., Henri C., Racine N., Denault A.Y., Rouleau J.L. (2020). Assessing Splanchnic Compartment Using Portal Venous Doppler and Impact of Adding It to the EVEREST Score for Risk Assessment in Heart Failure. CJC Open.

[68] Hao R., Zheng Y., Zhao Q., Chen J., Fan R., Chen P., Yin N., Qin H. (2024). Evaluation value of ultrasound on gastrointestinal function in patients with acute heart failure. Front. Cardiovasc. Med..

[69] Goncalvesova E., Lesny P., Luknar M., Solik P., Varga I. (2010). Changes of portal flow in heart failure patients with liver congestion. Bratisl. Lek. Listy.

[70] Bhardwaj V., Vikneswaran G., Rola P., Raju S., Bhat R.S., Jayakumar A., Alva A. (2020). Combination of Inferior Vena Cava Diameter, Hepatic Venous Flow, and Portal Vein Pulsatility Index: Venous Excess Ultrasound Score (VEXUS Score) in Predicting Acute Kidney Injury in Patients with Cardiorenal Syndrome: A Prospective Cohort Study. Indian J. Crit. Care Med..

[71] Gargani L., Pang P.S., Frassi F., Miglioranza M.H., Dini F.L., Landi P., Picano E. (2015). Persistent pulmonary congestion before discharge predicts rehospitalization in heart failure: A lung ultrasound study. Cardiovasc. Ultrasound.

[72] Singal A.K., Ahmad M., Soloway R.D. (2010). Duplex Doppler ultrasound examination of the portal venous system: An emerging novel technique for the estimation of portal vein pressure. Dig. Dis. Sci..

[73] Rengo C., Brevetti G., Sorrentino G., D’Amato T., Imparato M., Vitale D.F., Acanfora D., Rengo F. (1998). Portal vein pulsatility ratio provides a measure of right heart function in chronic heart failure. Ultrasound Med. Biol..

[74] Ciccone M.M., Iacoviello M., Gesualdo L., Puzzovivo A., Antoncecchi V., Doronzo A., Monitillo F., Citarelli G., Paradies V., Favale S. (2014). The renal arterial resistance index: A marker of renal function with an independent and incremental role in predicting heart failure progression. Eur. J. Heart Fail..

[75] Argaiz E.R. (2021). VExUS Nexus: Bedside Assessment of Venous Congestion. Adv. Chronic Kidney Dis..

[76] Denault A., Canty D., Azzam M., Amir A., Gebhard C.E. (2019). Whole body ultrasound in the operating room and intensive care unit. Korean J. Anesthesiol..

[77] Valentova M., von Haehling S., Bauditz J., Doehner W., Ebner N., Bekfani T., Elsner S., Sliziuk V., Scherbakov N., Murin J. (2016). Intestinal congestion and right ventricular dysfunction: A link with appetite loss, inflammation, and cachexia in chronic heart failure. Eur. Heart J..

[78] Sandek A., Swidsinski A., Schroedl W., Watson A., Valentova M., Herrmann R., Scherbakov N., Cramer L., Rauchhaus M., Grosse-Herrenthey A. (2014). Intestinal blood flow in patients with chronic heart failure: A link with bacterial growth, gastrointestinal symptoms, and cachexia. J. Am. Coll. Cardiol..

[79] Pellicori P., Zhang J., Cuthbert J., Urbinati A., Shah P., Kazmi S., Clark A.L., Cleland J.G.F. (2020). High-sensitivity C-reactive protein in chronic heart failure: Patient characteristics, phenotypes, and mode of death. Cardiovasc. Res..

[80] Ikeda Y., Ishii S., Maemura K., Oki T., Yazaki M., Fujita T., Nabeta T., Maekawa E., Koitabashi T., Ako J. (2021). Association between intestinal oedema and oral loop diuretic resistance in hospitalized patients with acute heart failure. ESC Heart Fail..

[81] Ciozda W., Kedan I., Kehl D.W., Zimmer R., Khandwalla R., Kimchi A. (2016). The efficacy of sonographic measurement of inferior vena cava diameter as an estimate of central venous pressure. Cardiovasc. Ultrasound.

[82] Zhao J., Wang G. (2016). Inferior Vena Cava Collapsibility Index is a Valuable and Non-Invasive Index for Elevated General Heart End-Diastolic Volume Index Estimation in Septic Shock Patients. Med. Sci. Monit..

[83] Aspromonte N., Fumarulo I., Petrucci L., Biferali B., Liguori A., Gasbarrini A., Massetti M., Miele L. (2023). The Liver in Heart Failure: From Biomarkers to Clinical Risk. Int. J. Mol. Sci..

[84] Wells M.L., Fenstad E.R., Poterucha J.T., Hough D.M., Young P.M., Araoz P.A., Ehman R.L., Venkatesh S.K. (2016). Imaging Findings of Congestive Hepatopathy. Radiographics.

[85] Maruyama H., Yokosuka O. (2017). Ultrasonography for Noninvasive Assessment of Portal Hypertension. Gut Liver.

[86] Gerstenmaier J.F., Gibson R.N. (2014). Ultrasound in chronic liver disease. Insights Imaging.

[87] Weinreb J., Kumari S., Phillips G., Pochaczevsky R. (1982). Portal vein measurements by real-time sonography. AJR Am. J. Roentgenol..

[88] Procopet B., Berzigotti A. (2017). Diagnosis of cirrhosis and portal hypertension: Imaging, non-invasive markers of fibrosis and liver biopsy. Gastroenterol. Rep..

[89] Allen L.A., Felker G.M., Pocock S., McMurray J.J., Pfeffer M.A., Swedberg K., Wang D., Yusuf S., Michelson E.L., Granger C.B. (2009). Liver function abnormalities and outcome in patients with chronic heart failure: Data from the Candesartan in Heart Failure: Assessment of Reduction in Mortality and Morbidity (CHARM) program. Eur. J. Heart Fail..

[90] Brankovic M., Lee P., Pyrsopoulos N., Klapholz M. (2023). Cardiac Syndromes in Liver Disease: A Clinical Conundrum. J. Clin. Transl. Hepatol..

[91] Harjola V.P., Mullens W., Banaszewski M., Bauersachs J., Brunner-La Rocca H.P., Chioncel O., Collins S.P., Doehner W., Filippatos G.S., Flammer A.J. (2017). Organ dysfunction, injury and failure in acute heart failure: From pathophysiology to diagnosis and management. A review on behalf of the Acute Heart Failure Committee of the Heart Failure Association (HFA) of the European Society of Cardiology (ESC). Eur. J. Heart Fail..

[92] Wang J., Wang K., Feng G., Tian X. (2024). Association Between the Albumin-Bilirubin (ALBI) Score and All-cause Mortality Risk in Intensive Care Unit Patients with Heart Failure. Glob. Heart.

[93] Tessler F.N., Gehring B.J., Gomes A.S., Perrella R.R., Ragavendra N., Busuttil R.W., Grant E.G. (1991). Diagnosis of portal vein thrombosis: Value of color Doppler imaging. AJR Am. J. Roentgenol..

[94] Hosoki T., Arisawa J., Marukawa T., Tokunaga K., Kuroda C., Kozuka T., Nakano S. (1990). Portal blood flow in congestive heart failure: Pulsed duplex sonographic findings. Radiology.

[95] Nelson R.C., Lovett K.E., Chezmar J.L., Moyers J.H., Torres W.E., Murphy F.B., Bernardino M.E. (1987). Comparison of pulsed Doppler sonography and angiography in patients with portal hypertension. AJR Am. J. Roentgenol..

[96] Gaiani S., Bolondi L., Li Bassi S., Santi V., Zironi G., Barbara L. (1989). Effect of meal on portal hemodynamics in healthy humans and in patients with chronic liver disease. Hepatology.

[97] Subramanyam B.R., Balthazar E.J., Madamba M.R., Raghavendra B.N., Horii S.C., Lefleur R.S. (1983). Sonography of portosystemic venous collaterals in portal hypertension. Radiology.

[98] Gibson R.N., Gibson P.R., Donlan J.D., Clunie D.A. (1989). Identification of a patent paraumbilical vein by using Doppler sonography: Importance in the diagnosis of portal hypertension. AJR Am. J. Roentgenol..

[99] Iranpour P., Lall C., Houshyar R., Helmy M., Yang A., Choi J.I., Ward G., Goodwin S.C. (2016). Altered Doppler flow patterns in cirrhosis patients: An overview. Ultrasonography.

[100] Afif A.M., Chang J.P., Wang Y.Y., Lau S.D., Deng F., Goh S.Y., Pwint M.K., Ooi C.C., Venkatanarasimha N., Lo R.H. (2017). A sonographic Doppler study of the hepatic vein, portal vein and hepatic artery in liver cirrhosis: Correlation of hepatic hemodynamics with clinical Child Pugh score in Singapore. Ultrasound.

[101] Baikpour M., Ozturk A., Dhyani M., Mercaldo N.D., Pierce T.T., Grajo J.R., Samir A.E. (2020). Portal Venous Pulsatility Index: A Novel Biomarker for Diagnosis of High-Risk Nonalcoholic Fatty Liver Disease. AJR Am. J. Roentgenol..

[102] Zhang L., Yin J., Duan Y., Yang Y., Yuan L., Cao T. (2011). Assessment of intrahepatic blood flow by Doppler ultrasonography: Relationship between the hepatic vein, portal vein, hepatic artery and portal pressure measured intraoperatively in patients with portal hypertension. BMC Gastroenterol..

[103] Abu-Yousef M.M., Milam S.G., Farner R.M. (1990). Pulsatile portal vein flow: A sign of tricuspid regurgitation on duplex Doppler sonography. AJR Am. J. Roentgenol..

[104] Gallix B.P., Taourel P., Dauzat M., Bruel J.M., Lafortune M. (1997). Flow pulsatility in the portal venous system: A study of Doppler sonography in healthy adults. AJR Am. J. Roentgenol..

[105] Caselitz M., Bahr M.J., Bleck J.S., Chavan A., Manns M.P., Wagner S., Gebel M. (2003). Sonographic criteria for the diagnosis of hepatic involvement in hereditary hemorrhagic telangiectasia (HHT). Hepatology.

[106] Abou-Arab O., Beyls C., Moussa M.D., Huette P., Beaudelot E., Guilbart M., De Broca B., Yzet T., Dupont H., Bouzerar R. (2022). Portal Vein Pulsatility Index as a Potential Risk of Venous Congestion Assessed by Magnetic Resonance Imaging: A Prospective Study on Healthy Volunteers. Front. Physiol..

[107] Gorg C., Seifart U., Zugmaier G. (2004). Color Doppler sonographic signs of respiration-dependent hepatofugal portal flow. J. Clin. Ultrasound.

[108] Hidajat N., Stobbe H., Griesshaber V., Felix R., Schroder R.J. (2005). Imaging and radiological interventions of portal vein thrombosis. Acta Radiol..

[109] Rossi S., Ghittoni G., Ravetta V., Torello Viera F., Rosa L., Serassi M., Scabini M., Vercelli A., Tinelli C., Dal Bello B. (2008). Contrast-enhanced ultrasonography and spiral computed tomography in the detection and characterization of portal vein thrombosis complicating hepatocellular carcinoma. Eur. Radiol..

[110] Altinkaya N., Koc Z., Ulusan S., Demir S., Gurel K. (2011). Effects of respiratory manoeuvres on hepatic vein Doppler waveform and flow velocities in a healthy population. Eur. J. Radiol..

[111] Piscaglia F., Donati G., Serra C., Muratori R., Solmi L., Gaiani S., Gramantieri L., Bolondi L. (2001). Value of splanchnic Doppler ultrasound in the diagnosis of portal hypertension. Ultrasound Med. Biol..

[112] Morales A., Hirsch M., Schneider D., Gonzalez D. (2020). Congestive hepatopathy: The role of the radiologist in the diagnosis. Diagn. Interv. Radiol..

[113] Catalano D., Caruso G., DiFazzio S., Carpinteri G., Scalisi N., Trovato G.M. (1998). Portal vein pulsatility ratio and heart failure. J. Clin. Ultrasound.

[114] Husain-Syed F., Birk H.W., Ronco C., Schormann T., Tello K., Richter M.J., Wilhelm J., Sommer N., Steyerberg E., Bauer P. (2019). Doppler-Derived Renal Venous Stasis Index in the Prognosis of Right Heart Failure. J. Am. Heart Assoc..

[115] Ikeda Y., Ishii S., Yazaki M., Fujita T., Iida Y., Kaida T., Nabeta T., Nakatani E., Maekawa E., Yanagisawa T. (2018). Portal congestion and intestinal edema in hospitalized patients with heart failure. Heart Vessel..

[116] Galindo P., Gasca C., Argaiz E.R., Koratala A. (2021). Point of care venous Doppler ultrasound: Exploring the missing piece of bedside hemodynamic assessment. World J. Crit. Care Med..

[117] Rola P., Miralles-Aguiar F., Argaiz E., Beaubien-Souligny W., Haycock K., Karimov T., Dinh V.A., Spiegel R. (2021). Clinical applications of the venous excess ultrasound (VExUS) score: Conceptual review and case series. Ultrasound J..

[118] Turk M., Koratala A., Robertson T., Kalagara H.K.P., Bronshteyn Y.S. (2025). Demystifying Venous Excess Ultrasound (VExUS): Image Acquisition and Interpretation. J. Vis. Exp. JoVE.

[119] Istrail L., Kiernan J., Stepanova M. (2023). A Novel Method for Estimating Right Atrial Pressure With Point-of-Care Ultrasound. J. Am. Soc. Echocardiogr..

[120] Deschamps J., Denault A., Galarza L., Rola P., Ledoux-Hutchinson L., Huard K., Gebhard C.E., Calderone A., Canty D., Beaubien-Souligny W. (2023). Venous Doppler to Assess Congestion: A Comprehensive Review of Current Evidence and Nomenclature. Ultrasound Med. Biol..

[121] Goldhammer E., Mesnick N., Abinader E.G., Sagiv M. (1999). Dilated inferior vena cava: A common echocardiographic finding in highly trained elite athletes. J. Am. Soc. Echocardiogr..

[122] Vivier E., Metton O., Piriou V., Lhuillier F., Cottet-Emard J.M., Branche P., Duperret S., Viale J.P. (2006). Effects of increased intra-abdominal pressure on central circulation. Br. J. Anaesth..

[123] Crespo-Aznarez S., Campos-Saenz de Santamaria A., Sanchez-Marteles M., Garces-Horna V., Josa-Laorden C., Gimenez-Lopez I., Perez-Calvo J.I., Rubio-Gracia J. (2023). The Association Between Intra-abdominal Pressure and Diuretic Response in Heart Failure. Curr. Heart Fail. Rep..

[124] Rora Bertovic M., Trkulja V., Curcic Karabaic E., Sundalic S., Bielen L., Ivicic T., Radonic R. (2025). Influence of Increased Intra-Abdominal Pressure on the Validity of Ultrasound-Derived Inferior Vena Cava Measurements for Estimating Central Venous Pressure. J. Clin. Med..

[125] Davison R., Cannon R. (1974). Estimation of central venous pressure by examination of jugular veins. Am. Heart J..

[126] Chayapinun V., Koratala A., Assavapokee T. (2024). Seeing beneath the surface: Harnessing point-of-care ultrasound for internal jugular vein evaluation. World J. Cardiol..

[127] Leal-Villarreal M.A.J., Aguirre-Villarreal D., Vidal-Mayo J.J., Argaiz E.R., Garcia-Juarez I. (2023). Correlation of Internal Jugular Vein Collapsibility With Central Venous Pressure in Patients With Liver Cirrhosis. Am. J. Gastroenterol..

[128] Bauman Z., Coba V., Gassner M., Amponsah D., Gallien J., Blyden D., Killu K. (2015). Inferior vena cava collapsibility loses correlation with internal jugular vein collapsibility during increased thoracic or intra-abdominal pressure. J. Ultrasound.

[129] Benkreira A., Beaubien-Souligny W., Mailhot T., Bouabdallaoui N., Robillard P., Desjardins G., Lamarche Y., Cossette S., Denault A. (2019). Portal Hypertension Is Associated With Congestive Encephalopathy and Delirium After Cardiac Surgery. Can. J. Cardiol..

[130] Croquette M., Puyade M., Montani D., Jutant E.M., De Gea M., Laneelle D., Thollot C., Trihan J.E. (2023). Diagnostic Performance of Pulsed Doppler Ultrasound of the Common Femoral Vein to Detect Elevated Right Atrial Pressure in Pulmonary Hypertension. J. Cardiovasc. Transl. Res..

[131] Croquette M., Larrieu Ardilouze E., Beaufort C., Jutant E.M., Puyade M., Montani D., Thollot C., Laneelle D., De Gea M., Trihan J.E. (2025). Femoral venous stasis index predicts elevated right atrial pressure and mortality in pulmonary hypertension. ERJ Open Res..

[132] Bhardwaj V., Rola P., Denault A., Vikneswaran G., Spiegel R. (2023). Femoral vein pulsatility: A simple tool for venous congestion assessment. Ultrasound J..

[133] Murayama M., Kaga S., Okada K., Iwano H., Nakabachi M., Yokoyama S., Nishino H., Tsujinaga S., Chiba Y., Ishizaka S. (2022). Clinical Utility of Superior Vena Cava Flow Velocity Waveform Measured from the Subcostal Window for Estimating Right Atrial Pressure. J. Am. Soc. Echocardiogr..

[134] Murayama M., Kaga S., Onoda A., Nishino H., Yokoyama S., Goto M., Suzuki Y., Yanagi Y., Shimono Y., Nakamura K. (2024). Head-to-Head Comparison of Hepatic Vein and Superior Vena Cava Flow Velocity Waveform Analyses for Predicting Elevated Right Atrial Pressure. Ultrasound Med. Biol..

[135] Lee J.H., Denault A.Y., Beaubien-Souligny W., Cho S.A., Ji S.H., Jang Y.E., Kim E.H., Kim H.S., Kim J.T. (2023). Evaluation of Portal, Splenic, and Hepatic Vein Flows in Children Undergoing Congenital Heart Surgery. J. Cardiothorac. Vasc. Anesth..

[136] Gonzalez C., Chamberland M.E., Aldred M.P., Couture E., Beaubien-Souligny W., Calderone A., Lamarche Y., Denault A. (2022). Constrictive pericarditis: Portal, splenic, and femoral venous Doppler pulsatility: A case series. Can. J. Anaesth. J. Can. Anesth..

[137] Martinez-Noguera A., Montserrat E., Torrubia S., Villalba J. (2002). Doppler in hepatic cirrhosis and chronic hepatitis. Semin. Ultrasound CT MR.

[138] Kim M.Y., Baik S.K., Park D.H., Lim D.W., Kim J.W., Kim H.S., Kwon S.O., Kim Y.J., Chang S.J., Lee S.S. (2007). Damping index of Doppler hepatic vein waveform to assess the severity of portal hypertension and response to propranolol in liver cirrhosis: A prospective nonrandomized study. Liver Int..

[139] Dodd G.D., Memel D.S., Zajko A.B., Baron R.L., Santaguida L.A. (1994). Hepatic artery stenosis and thrombosis in transplant recipients: Doppler diagnosis with resistive index and systolic acceleration time. Radiology.

[140] Lemmer A., VanWagner L., Ganger D. (2017). Congestive hepatopathy: Differentiating congestion from fibrosis. Clin. Liver Dis..

[141] Schneider A.W., Kalk J.F., Klein C.P. (1999). Hepatic arterial pulsatility index in cirrhosis: Correlation with portal pressure. J. Hepatol..

[142] Bolognesi M., Sacerdoti D., Merkel C., Gerunda G., Maffei-Faccioli A., Angeli P., Jemmolo R.M., Bombonato G., Gatta A. (1996). Splenic Doppler impedance indices: Influence of different portal hemodynamic conditions. Hepatology.

[143] Bolognesi M., Quaglio C., Bombonato G., Gaiani S., Pesce P., Bizzotto P., Favaretto E., Gatta A., Sacerdoti D. (2012). Splenic Doppler impedance indices estimate splenic congestion in patients with right-sided or congestive heart failure. Ultrasound Med. Biol..

[144] Ronco C., Haapio M., House A.A., Anavekar N., Bellomo R. (2008). Cardiorenal syndrome. J. Am. Coll. Cardiol..

[145] Boddi M., Natucci F., Ciani E. (2015). The internist and the renal resistive index: Truths and doubts. Intern. Emerg. Med..

[146] George S.M., Kalantarinia K. (2011). The role of imaging in the management of cardiorenal syndrome. Int. J. Nephrol..

[147] Kim S.H., Kim W.H., Choi B.I., Kim C.W. (1992). Duplex Doppler US in patients with medical renal disease: Resistive index vs serum creatinine level. Clin. Radiol..

[148] Radermacher J., Chavan A., Bleck J., Vitzthum A., Stoess B., Gebel M.J., Galanski M., Koch K.M., Haller H. (2001). Use of Doppler ultrasonography to predict the outcome of therapy for renal-artery stenosis. N. Engl. J. Med..

[149] Kim H.L., Jo S.H. (2024). Arterial Stiffness and Heart Failure With Preserved Ejection Fraction. J. Korean Med. Sci..

[150] Doyle A., Mark P.B., Johnston N., Foster J., Connell J.M., Dargie H., Jardine A., Padmanabhan N. (2008). Aortic stiffness and diastolic flow abnormalities in end-stage renal disease assessed by magnetic resonance imaging. Nephron Clin. Pract..

[151] Chirinos J.A., Townsend R.R. (2014). Systemic arterial hemodynamics and the “renal resistive index”: What is in a name?. J. Clin. Hypertens..

[152] O’Neill W.C. (2014). Renal resistive index: A case of mistaken identity. Hypertension.

[153] Naesens M., Heylen L., Lerut E., Claes K., De Wever L., Claus F., Oyen R., Kuypers D., Evenepoel P., Bammens B. (2013). Intrarenal resistive index after renal transplantation. N. Engl. J. Med..

[154] Tamayo-Gutierrez A., Ibrahim H.N. (2022). The Kidney in Heart Failure: The Role of Venous Congestion. Methodist. Debakey Cardiovasc. J..

[155] Pena D.L., Iliesiu A.M., Aurelian J., Grigore M., Hodorogea A.S., Ciobanu A., Weiss E., Badila E., Balahura A.M. (2025). Assessment of Decongestion Status Before Discharge in Acute Decompensated Heart Failure: A Review of Clinical, Biochemical, and Imaging Tools and Their Impact on Management Decisions. Medicina.

[156] Boddi M., Bonizzoli M., Chiostri M., Begliomini D., Molinaro A., Tadini Buoninsegni L., Gensini G.F., Peris A. (2016). Renal Resistive Index and mortality in critical patients with acute kidney injury. Eur. J. Clin. Investig..

[157] Toledo C., Thomas G., Schold J.D., Arrigain S., Gornik H.L., Nally J.V., Navaneethan S.D. (2015). Renal resistive index and mortality in chronic kidney disease. Hypertension.

[158] Darabont R., Mihalcea D., Vinereanu D. (2023). Current Insights into the Significance of the Renal Resistive Index in Kidney and Cardiovascular Disease. Diagnostics.

[159] Mahfoud F., Cremers B., Janker J., Link B., Vonend O., Ukena C., Linz D., Schmieder R., Rump L.C., Kindermann I. (2012). Renal hemodynamics and renal function after catheter-based renal sympathetic denervation in patients with resistant hypertension. Hypertension.

[160] Tedesco M.A., Natale F., Mocerino R., Tassinario G., Calabro R. (2007). Renal resistive index and cardiovascular organ damage in a large population of hypertensive patients. J. Hum. Hypertens..

[161] Viazzi F., Leoncini G., Derchi L.E., Pontremoli R. (2014). Ultrasound Doppler renal resistive index: A useful tool for the management of the hypertensive patient. J. Hypertens..

[162] Paulus W.J., Tschope C. (2013). A novel paradigm for heart failure with preserved ejection fraction: Comorbidities drive myocardial dysfunction and remodeling through coronary microvascular endothelial inflammation. J. Am. Coll. Cardiol..

[163] Redfield M.M. (2016). Heart Failure with Preserved Ejection Fraction. N. Engl. J. Med..

[164] Sanders-van Wijk S., van Empel V., Davarzani N., Maeder M.T., Handschin R., Pfisterer M.E., Brunner-La Rocca H.P., TIME-CHF investigators (2015). Circulating biomarkers of distinct pathophysiological pathways in heart failure with preserved vs. reduced left ventricular ejection fraction. Eur. J. Heart Fail..

[165] Ponikowski P., Voors A.A., Anker S.D., Bueno H., Cleland J.G., Coats A.J., Falk V., Gonzalez-Juanatey J.R., Harjola V.P., Jankowska E.A. (2016). 2016 ESC Guidelines for the diagnosis and treatment of acute and chronic heart failure: The Task Force for the diagnosis and treatment of acute and chronic heart failure of the European Society of Cardiology (ESC). Developed with the special contribution of the Heart Failure Association (HFA) of the ESC. Eur. J. Heart Fail..

[166] Simonneau G., Montani D., Celermajer D.S., Denton C.P., Gatzoulis M.A., Krowka M., Williams P.G., Souza R. (2019). Haemodynamic definitions and updated clinical classification of pulmonary hypertension. Eur. Respir. J..

[167] Li X.N., Liu Y.T., Kang S., Qu Yang D.Z., Xiao H.Y., Ma W.K., Shen C.X., Pan J.W. (2024). Interdependence between myocardial deformation and perfusion in patients with T2DM and HFpEF: A feature-tracking and stress perfusion CMR study. Cardiovasc. Diabetol..

[168] Lin M., Guo J., Tao H., Gu Z., Tang W., Zhou F., Jiang Y., Zhang R., Jia D., Sun Y. (2025). Circulating mediators linking cardiometabolic diseases to HFpEF: A mediation Mendelian randomization analysis. Cardiovasc. Diabetol..

[169] Carluccio E., Biagioli P., Reboldi G., Mengoni A., Lauciello R., Zuchi C., D’Addario S., Bardelli G., Ambrosio G. (2023). Left ventricular remodeling response to SGLT2 inhibitors in heart failure: An updated meta-analysis of randomized controlled studies. Cardiovasc. Diabetol..

[170] Correale M., D’Alessandro D., Tricarico L., Ceci V., Mazzeo P., Capasso R., Ferrara S., Barile M., Di Nunno N., Rossi L. (2024). Left ventricular reverse remodeling after combined ARNI and SGLT2 therapy in heart failure patients with reduced or mildly reduced ejection fraction. Int. J. Cardiol. Heart Vasc..

[171] Veltmann C., Duncker D., Doering M., Gummadi S., Robertson M., Wittlinger T., Colley B.J., Perings C., Jonsson O., Bauersachs J. (2024). Therapy duration and improvement of ventricular function in de novo heart failure: The Heart Failure Optimization study. Eur. Heart J..

[172] Ichimura K., Boehm M., Andruska A.M., Zhang F., Schimmel K., Bonham S., Kabiri A., Kheyfets V.O., Ichimura S., Reddy S. (2024). 3D Imaging Reveals Complex Microvascular Remodeling in the Right Ventricle in Pulmonary Hypertension. Circ. Res..

[173] Mendiola E.A., da Silva Goncalves Bos D., Leichter D.M., Vang A., Zhang P., Leary O.P., Gilbert R.J., Avazmohammadi R., Choudhary G. (2023). Right Ventricular Architectural Remodeling and Functional Adaptation in Pulmonary Hypertension. Circ. Heart Fail..

[174] Ito K., Kato S., Yasuda N., Sawamura S., Fukui K., Iwasawa T., Ogura T., Utsunomiya D. (2025). Integrating CT-Based Lung Fibrosis and MRI-Derived Right Ventricular Function for the Detection of Pulmonary Hypertension in Interstitial Lung Disease. J. Clin. Med..

[175] Ma Y., Guo D., Wang J., Gong J., Hu H., Zhang X., Wang Y., Yang Y., Lv X., Li Y. (2024). Effects of right ventricular remodeling in chronic thromboembolic pulmonary hypertension on the outcomes of balloon pulmonary angioplasty: A 2D-speckle tracking echocardiography study. Respir. Res..

[176] Galderisi M., Cosyns B., Edvardsen T., Cardim N., Delgado V., Di Salvo G., Donal E., Sade L.E., Ernande L., Garbi M. (2017). Standardization of adult transthoracic echocardiography reporting in agreement with recent chamber quantification, diastolic function, and heart valve disease recommendations: An expert consensus document of the European Association of Cardiovascular Imaging. Eur. Heart J. Cardiovasc. Imaging.

[177] Yoshimura R., Hayashi O., Horio T., Fujiwara R., Matsuoka Y., Yokouchi G., Sakamoto Y., Matsumoto N., Fukuda K., Shimizu M. (2023). The E/e’ ratio on echocardiography as an independent predictor of the improvement of left ventricular contraction in patients with heart failure with reduced ejection fraction. J. Clin. Ultrasound.

[178] Upadhya B., Rose G.A., Stacey R.B., Palma R.A., Ryan T., Pendyal A., Kelsey A.M. (2025). The role of echocardiography in the diagnosis of heart failure with preserved ejection fraction. Heart Fail. Rev..

[179] Pender A., Lewis-Owona J., Ekiyoyo A., Stoddard M. (2025). Echocardiography and Heart Failure: An Echocardiographic Decision Aid for the Diagnosis and Management of Cardiomyopathies. Curr. Cardiol. Rep..

[180] McDonagh T.A., Metra M., Adamo M., Gardner R.S., Baumbach A., Bohm M., Burri H., Butler J., Celutkiene J., Chioncel O. (2021). 2021 ESC Guidelines for the diagnosis and treatment of acute and chronic heart failure. Eur. Heart J..

[181] Grigore A.M., Grigore M., Balahura A.M., Uscoiu G., Verde I., Nicolae C., Badila E., Iliesiu A.M. (2025). The Role of the Estimated Plasma Volume Variation in Assessing Decongestion in Patients with Acute Decompensated Heart Failure. Biomedicines.

[182] Henry J.A., Couch L.S., Rider O.J. (2024). Myocardial Metabolism in Heart Failure with Preserved Ejection Fraction. J. Clin. Med..

[183] Sun Q., Wagg C.S., Wong N., Wei K., Ketema E.B., Zhang L., Fang L., Seubert J.M., Lopaschuk G.D. (2025). Alterations of myocardial ketone metabolism in heart failure with preserved ejection fraction (HFpEF). ESC Heart Fail..

[184] Nagueh S.F., Sanborn D.Y., Oh J.K., Anderson B., Billick K., Derumeaux G., Klein A., Koulogiannis K., Mitchell C., Shah A. (2025). Recommendations for the Evaluation of Left Ventricular Diastolic Function by Echocardiography and for Heart Failure With Preserved Ejection Fraction Diagnosis: An Update From the American Society of Echocardiography. J. Am. Soc. Echocardiogr..

[185] Smiseth O.A., Morris D.A., Cardim N., Cikes M., Delgado V., Donal E., Flachskampf F.A., Galderisi M., Gerber B.L., Gimelli A. (2022). Multimodality imaging in patients with heart failure and preserved ejection fraction: An expert consensus document of the European Association of Cardiovascular Imaging. Eur. Heart J. Cardiovasc. Imaging.

[186] Harjola V.P., Mebazaa A., Celutkiene J., Bettex D., Bueno H., Chioncel O., Crespo-Leiro M.G., Falk V., Filippatos G., Gibbs S. (2016). Contemporary management of acute right ventricular failure: A statement from the Heart Failure Association and the Working Group on Pulmonary Circulation and Right Ventricular Function of the European Society of Cardiology. Eur. J. Heart Fail..

[187] Mukherjee M., Rudski L.G., Addetia K., Afilalo J., D’Alto M., Freed B.H., Friend L.B., Gargani L., Grapsa J., Hassoun P.M. (2025). Guidelines for the Echocardiographic Assessment of the Right Heart in Adults and Special Considerations in Pulmonary Hypertension: Recommendations from the American Society of Echocardiography. J. Am. Soc. Echocardiogr..

[188] Cordina R.L., Playford D., Lang I., Celermajer D.S. (2019). State-of-the-Art Review: Echocardiography in Pulmonary Hypertension. Heart Lung Circ..

[189] Labrada L., Vaidy A., Vaidya A. (2023). Right ventricular assessment in pulmonary hypertension. Curr. Opin. Pulm. Med..

[190] Tsipis A., Petropoulou E. (2022). Echocardiography in the Evaluation of the Right Heart. US Cardiol..

[191] D’Alto M., Di Maio M., Romeo E., Argiento P., Blasi E., Di Vilio A., Rea G., D’Andrea A., Golino P., Naeije R. (2022). Echocardiographic probability of pulmonary hypertension: A validation study. Eur. Respir. J..

[192] Borlaug B.A., Sharma K., Shah S.J., Ho J.E. (2023). Heart Failure With Preserved Ejection Fraction: JACC Scientific Statement. J. Am. Coll. Cardiol..

[193] Pastore M.C., Mandoli G.E., Aboumarie H.S., Santoro C., Bandera F., D’Andrea A., Benfari G., Esposito R., Evola V., Sorrentino R. (2020). Basic and advanced echocardiography in advanced heart failure: An overview. Heart Fail. Rev..

[194] Li J., Song Y., Chen F. (2024). Evaluating the impact of Sacubitril/valsartan on diastolic function in patients with heart failure: A systematic review and meta-analysis. Medicine.

[195] Galzerano D., Savo M.T., Castaldi B., Kholaif N., Khaliel F., Pozza A., Aljheish S., Cattapan I., Martini M., Lassandro E. (2024). Transforming Heart Failure Management: The Power of Strain Imaging, 3D Imaging, and Vortex Analysis in Echocardiography. J. Clin. Med..

[196] Nuzzi V., Manca P., Mule M., Leone S., Fazzini L., Cipriani M.G., Faletra F.F. (2024). Contemporary clinical role of echocardiography in patients with advanced heart failure. Heart Fail. Rev..

[197] Mitchell C., Rahko P.S., Blauwet L.A., Canaday B., Finstuen J.A., Foster M.C., Horton K., Ogunyankin K.O., Palma R.A., Velazquez E.J. (2019). Guidelines for Performing a Comprehensive Transthoracic Echocardiographic Examination in Adults: Recommendations from the American Society of Echocardiography. J. Am. Soc. Echocardiogr..

[198] Colonna P., Pinto F.J., Sorino M., Bovenzi F., D’Agostino C., de Luca I. (2005). The emerging role of echocardiography in the screening of patients at risk of heart failure. Am. J. Cardiol..

[199] Gong F.F., Campbell D.J., Prior D.L. (2017). Noninvasive Cardiac Imaging and the Prediction of Heart Failure Progression in Preclinical Stage A/B Subjects. JACC Cardiovasc. Imaging.

[200] Writing Group M., Doherty J.U., Kort S., Mehran R., Schoenhagen P., Soman P., Rating Panel M., Dehmer G.J., Doherty J.U., Schoenhagen P. (2019). ACC/AATS/AHA/ASE/ASNC/HRS/SCAI/SCCT/SCMR/STS 2019 Appropriate Use Criteria for Multimodality Imaging in the Assessment of Cardiac Structure and Function in Nonvalvular Heart Disease: A Report of the American College of Cardiology Appropriate Use Criteria Task Force, American Association for Thoracic Surgery, American Heart Association, American Society of Echocardiography, American Society of Nuclear Cardiology, Heart Rhythm Society, Society for Cardiovascular Angiography and Interventions, Society of Cardiovascular Computed Tomography, Society for Cardiovascular Magnetic Resonance, and the Society of Thoracic Surgeons. J. Am. Soc. Echocardiogr..

[201] Edvardsen T., Asch F.M., Davidson B., Delgado V., DeMaria A., Dilsizian V., Gaemperli O., Garcia M.J., Kamp O., Lee D.C. (2022). Non-Invasive Imaging in Coronary Syndromes: Recommendations of The European Association of Cardiovascular Imaging and the American Society of Echocardiography, in Collaboration with The American Society of Nuclear Cardiology, Society of Cardiovascular Computed Tomography, and Society for Cardiovascular Magnetic Resonance. J. Am. Soc. Echocardiogr..

[202] Zoghbi W.A., Jone P.N., Chamsi-Pasha M.A., Chen T., Collins K.A., Desai M.Y., Grayburn P., Groves D.W., Hahn R.T., Little S.H. (2024). Guidelines for the Evaluation of Prosthetic Valve Function With Cardiovascular Imaging: A Report From the American Society of Echocardiography Developed in Collaboration With the Society for Cardiovascular Magnetic Resonance and the Society of Cardiovascular Computed Tomography. J. Am. Soc. Echocardiogr..

[203] Lang R.M., Badano L.P., Mor-Avi V., Afilalo J., Armstrong A., Ernande L., Flachskampf F.A., Foster E., Goldstein S.A., Kuznetsova T. (2015). Recommendations for cardiac chamber quantification by echocardiography in adults: An update from the American Society of Echocardiography and the European Association of Cardiovascular Imaging. J. Am. Soc. Echocardiogr..

[204] Chioncel O., Mebazaa A., Harjola V.P., Coats A.J., Piepoli M.F., Crespo-Leiro M.G., Laroche C., Seferovic P.M., Anker S.D., Ferrari R. (2017). Clinical phenotypes and outcome of patients hospitalized for acute heart failure: The ESC Heart Failure Long-Term Registry. Eur. J. Heart Fail..

[205] Pugliese N.R., Mazzola M., Bandini G., Barbieri G., Spinelli S., De Biase N., Masi S., Moggi-Pignone A., Ghiadoni L., Taddei S. (2023). Prognostic Role of Sonographic Decongestion in Patients with Acute Heart Failure with Reduced and Preserved Ejection Fraction: A Multicentre Study. J. Clin. Med..

[206] Wang C.S., FitzGerald J.M., Schulzer M., Mak E., Ayas N.T. (2005). Does this dyspneic patient in the emergency department have congestive heart failure?. JAMA.

[207] Lichtenstein D., Meziere G., Biderman P., Gepner A., Barre O. (1997). The comet-tail artifact. An ultrasound sign of alveolar-interstitial syndrome. Am. J. Respir. Crit. Care Med..

[208] Volpicelli G., Elbarbary M., Blaivas M., Lichtenstein D.A., Mathis G., Kirkpatrick A.W., Melniker L., Gargani L., Noble V.E., Via G. (2012). International evidence-based recommendations for point-of-care lung ultrasound. Intensive Care Med..

[209] Picano E., Frassi F., Agricola E., Gligorova S., Gargani L., Mottola G. (2006). Ultrasound lung comets: A clinically useful sign of extravascular lung water. J. Am. Soc. Echocardiogr..

[210] Gargani L. (2011). Lung ultrasound: A new tool for the cardiologist. Cardiovasc. Ultrasound.

[211] Chouihed T., Coiro S., Zannad F., Girerd N. (2016). Lung ultrasound: A diagnostic and prognostic tool at every step in the pathway of care for acute heart failure. Am. J. Emerg. Med..

[212] Mottola C., Girerd N., Coiro S., Lamiral Z., Rossignol P., Frimat L., Girerd S. (2018). Evaluation of Subclinical Fluid Overload Using Lung Ultrasound and Estimated Plasma Volume in the Postoperative Period Following Kidney Transplantation. Transplant. Proc..

[213] Lichtenstein D.A., Meziere G.A. (2008). Relevance of lung ultrasound in the diagnosis of acute respiratory failure: The BLUE protocol. Chest.

[214] Dubon-Peralta E.E., Lorenzo-Villalba N., Garcia-Klepzig J.L., Andres E., Mendez-Bailon M. (2022). Prognostic value of B lines detected with lung ultrasound in acute heart failure. A systematic review. J. Clin. Ultrasound.

[215] Gargani L., Volpicelli G. (2014). How I do it: Lung ultrasound. Cardiovasc. Ultrasound.

[216] Yuriditsky E., Horowitz J.M., Panebianco N.L., Sauthoff H., Saric M. (2021). Lung Ultrasound Imaging: A Primer for Echocardiographers. J. Am. Soc. Echocardiogr..

[217] Lichtenstein D.A. (2014). Lung ultrasound in the critically ill. Ann. Intensive Care.

[218] Biswas A., Lascano J.E., Mehta H.J., Faruqi I. (2017). The Utility of the “Shred Sign” in the Diagnosis of Acute Respiratory Distress Syndrome Resulting from Multifocal Pneumonia. Am. J. Respir. Crit. Care Med..

[219] Soldati G., Demi M., Inchingolo R., Smargiassi A., Demi L. (2016). On the Physical Basis of Pulmonary Sonographic Interstitial Syndrome. J. Ultrasound Med..

[220] Soldati G., Demi M. (2017). The use of lung ultrasound images for the differential diagnosis of pulmonary and cardiac interstitial pathology. J. Ultrasound.

[221] Laursen C.B., Clive A., Hallifax R., Pietersen P.I., Asciak R., Davidsen J.R., Bhatnagar R., Bedawi E.O., Jacobsen N., Coleman C. (2021). European Respiratory Society statement on thoracic ultrasound. Eur. Respir. J..

[222] Marini T.J., Rubens D.J., Zhao Y.T., Weis J., O’Connor T.P., Novak W.H., Kaproth-Joslin K.A. (2021). Lung Ultrasound: The Essentials. Radiol. Cardiothorac. Imaging.

[223] Picano E., Scali M.C., Ciampi Q., Lichtenstein D. (2018). Lung Ultrasound for the Cardiologist. JACC Cardiovasc. Imaging.

[224] Jambrik Z., Monti S., Coppola V., Agricola E., Mottola G., Miniati M., Picano E. (2004). Usefulness of ultrasound lung comets as a nonradiologic sign of extravascular lung water. Am. J. Cardiol..

[225] Volpicelli G., Mussa A., Garofalo G., Cardinale L., Casoli G., Perotto F., Fava C., Frascisco M. (2006). Bedside lung ultrasound in the assessment of alveolar-interstitial syndrome. Am. J. Emerg. Med..

[226] Buessler A., Chouihed T., Duarte K., Bassand A., Huot-Marchand M., Gottwalles Y., Penine A., Andre E., Nace L., Jaeger D. (2020). Accuracy of Several Lung Ultrasound Methods for the Diagnosis of Acute Heart Failure in the ED: A Multicenter Prospective Study. Chest.

[227] Liteplo A.S., Marill K.A., Villen T., Miller R.M., Murray A.F., Croft P.E., Capp R., Noble V.E. (2009). Emergency thoracic ultrasound in the differentiation of the etiology of shortness of breath (ETUDES): Sonographic B-lines and N-terminal pro-brain-type natriuretic peptide in diagnosing congestive heart failure. Acad. Emerg. Med..

[228] Platz E., Jhund P.S., Girerd N., Pivetta E., McMurray J.J.V., Peacock W.F., Masip J., Martin-Sanchez F.J., Miro O., Price S. (2019). Expert consensus document: Reporting checklist for quantification of pulmonary congestion by lung ultrasound in heart failure. Eur. J. Heart Fail..

[229] Pivetta E., Goffi A., Nazerian P., Castagno D., Tozzetti C., Tizzani P., Tizzani M., Porrino G., Ferreri E., Busso V. (2019). Lung ultrasound integrated with clinical assessment for the diagnosis of acute decompensated heart failure in the emergency department: A randomized controlled trial. Eur. J. Heart Fail..

[230] Frassi F., Gargani L., Gligorova S., Ciampi Q., Mottola G., Picano E. (2007). Clinical and echocardiographic determinants of ultrasound lung comets. Eur. J. Echocardiogr..

[231] Volpicelli G., Caramello V., Cardinale L., Mussa A., Bar F., Frascisco M.F. (2008). Bedside ultrasound of the lung for the monitoring of acute decompensated heart failure. Am. J. Emerg. Med..

[232] Cortellaro F., Ceriani E., Spinelli M., Campanella C., Bossi I., Coen D., Casazza G., Cogliati C. (2017). Lung ultrasound for monitoring cardiogenic pulmonary edema. Intern. Emerg. Med..

[233] Ohman J., Harjola V.P., Karjalainen P., Lassus J. (2018). Assessment of early treatment response by rapid cardiothoracic ultrasound in acute heart failure: Cardiac filling pressures, pulmonary congestion and mortality. Eur. Heart J. Acute Cardiovasc. Care.

[234] Facchini C., Malfatto G., Giglio A., Facchini M., Parati G., Branzi G. (2016). Lung ultrasound and transthoracic impedance for noninvasive evaluation of pulmonary congestion in heart failure. J. Cardiovasc. Med..

[235] Coiro S., Porot G., Rossignol P., Ambrosio G., Carluccio E., Tritto I., Huttin O., Lemoine S., Sadoul N., Donal E. (2016). Prognostic value of pulmonary congestion assessed by lung ultrasound imaging during heart failure hospitalisation: A two-centre cohort study. Sci. Rep..

[236] Platz E., Campbell R.T., Claggett B., Lewis E.F., Groarke J.D., Docherty K.F., Lee M.M.Y., Merz A.A., Silverman M., Swamy V. (2019). Lung Ultrasound in Acute Heart Failure: Prevalence of Pulmonary Congestion and Short- and Long-Term Outcomes. JACC Heart Fail..

[237] Coiro S., Rossignol P., Ambrosio G., Carluccio E., Alunni G., Murrone A., Tritto I., Zannad F., Girerd N. (2015). Prognostic value of residual pulmonary congestion at discharge assessed by lung ultrasound imaging in heart failure. Eur. J. Heart Fail..

[238] Rivas-Lasarte M., Maestro A., Fernandez-Martinez J., Lopez-Lopez L., Sole-Gonzalez E., Vives-Borras M., Montero S., Mesado N., Pirla M.J., Mirabet S. (2020). Prevalence and prognostic impact of subclinical pulmonary congestion at discharge in patients with acute heart failure. ESC Heart Fail..

[239] Rastogi T., Bozec E., Pellicori P., Bayes-Genis A., Coiro S., Domingo M., Gargani L., Palazzuoli A., Girerd N. (2022). Prognostic Value and Therapeutic Utility of Lung Ultrasound in Acute and Chronic Heart Failure: A Meta-Analysis. JACC Cardiovasc. Imaging.

[240] Miglioranza M.H., Gargani L., Sant’Anna R.T., Rover M.M., Martins V.M., Mantovani A., Weber C., Moraes M.A., Feldman C.J., Kalil R.A. (2013). Lung ultrasound for the evaluation of pulmonary congestion in outpatients: A comparison with clinical assessment, natriuretic peptides, and echocardiography. JACC Cardiovasc. Imaging.

[241] Pellicori P., Shah P., Cuthbert J., Urbinati A., Zhang J., Kallvikbacka-Bennett A., Clark A.L., Cleland J.G.F. (2019). Prevalence, pattern and clinical relevance of ultrasound indices of congestion in outpatients with heart failure. Eur. J. Heart Fail..

[242] Dwyer K.H., Merz A.A., Lewis E.F., Claggett B.L., Crousillat D.R., Lau E.S., Silverman M.B., Peck J., Rivero J., Cheng S. (2018). Pulmonary Congestion by Lung Ultrasound in Ambulatory Patients With Heart Failure With Reduced or Preserved Ejection Fraction and Hypertension. J. Card. Fail..

[243] Domingo M., Conangla L., Lupon J., de Antonio M., Moliner P., Santiago-Vacas E., Codina P., Zamora E., Cediel G., Gonzalez B. (2021). Prognostic value of lung ultrasound in chronic stable ambulatory heart failure patients. Rev. Esp. Cardiol..

[244] Morvai-Illes B., Polestyuk-Nemeth N., Szabo I.A., Monoki M., Gargani L., Picano E., Varga A., Agoston G. (2021). The Prognostic Value of Lung Ultrasound in Patients With Newly Diagnosed Heart Failure With Preserved Ejection Fraction in the Ambulatory Setting. Front. Cardiovasc. Med..

[245] Rivas-Lasarte M., Alvarez-Garcia J., Fernandez-Martinez J., Maestro A., Lopez-Lopez L., Sole-Gonzalez E., Pirla M.J., Mesado N., Mirabet S., Fluvia P. (2019). Lung ultrasound-guided treatment in ambulatory patients with heart failure: A randomized controlled clinical trial (LUS-HF study). Eur. J. Heart Fail..

[246] Araiza-Garaygordobil D., Gopar-Nieto R., Martinez-Amezcua P., Cabello-Lopez A., Alanis-Estrada G., Luna-Herbert A., Gonzalez-Pacheco H., Paredes-Paucar C.P., Sierra-Lara M.D., Briseno-De la Cruz J.L. (2020). A randomized controlled trial of lung ultrasound-guided therapy in heart failure (CLUSTER-HF study). Am. Heart J..

[247] Reddy Y.N.V., Obokata M., Wiley B., Koepp K.E., Jorgenson C.C., Egbe A., Melenovsky V., Carter R.E., Borlaug B.A. (2019). The haemodynamic basis of lung congestion during exercise in heart failure with preserved ejection fraction. Eur. Heart J..

[248] Simonovic D., Coiro S., Carluccio E., Girerd N., Deljanin-Ilic M., Cattadori G., Ambrosio G. (2018). Exercise elicits dynamic changes in extravascular lung water and haemodynamic congestion in heart failure patients with preserved ejection fraction. Eur. J. Heart Fail..

[249] Scali M.C., Cortigiani L., Simionuc A., Gregori D., Marzilli M., Picano E. (2017). Exercise-induced B-lines identify worse functional and prognostic stage in heart failure patients with depressed left ventricular ejection fraction. Eur. J. Heart Fail..

[250] Coiro S., Simonovic D., Deljanin-Ilic M., Duarte K., Carluccio E., Cattadori G., Girerd N., Ambrosio G. (2020). Prognostic Value of Dynamic Changes in Pulmonary Congestion During Exercise Stress Echocardiography in Heart Failure With Preserved Ejection Fraction. Circ. Heart Fail..

[251] Pugliese N.R., Masi S. (2020). The emerging role of endothelial function in cardiovascular oncology. Eur. J. Prev. Cardiol..

[252] Fudim M., Kaye D.M., Borlaug B.A., Shah S.J., Rich S., Kapur N.K., Costanzo M.R., Brener M.I., Sunagawa K., Burkhoff D. (2022). Venous Tone and Stressed Blood Volume in Heart Failure: JACC Review Topic of the Week. J. Am. Coll. Cardiol..

[253] Fudim M., Hernandez A.F., Felker G.M. (2017). Role of Volume Redistribution in the Congestion of Heart Failure. J. Am. Heart Assoc..

[254] Kanitkar S., Soni K., Vaishnav B. (2024). Venous Excess Ultrasound for Fluid Assessment in Complex Cardiac Patients With Acute Kidney Injury. Cureus.

[255] Melo R.H., Gioli-Pereira L., Melo E., Rola P. (2025). Venous excess ultrasound score association with acute kidney injury in critically ill patients: A systematic review and meta-analysis of observational studies. Ultrasound J..

[256] Dimopoulos S., Antonopoulos M. (2024). Portal vein pulsatility: An important sonographic tool assessment of systemic congestion for critical ill patients. World J. Cardiol..

[257] Jain C.C., Reddy Y.N.V. (2022). Approach to Echocardiography in Heart Failure with Preserved Ejection Fraction. Cardiol. Clin..

[258] Torres-Arrese M., Mata-Martinez A., Luordo-Tedesco D., Garcia-Casasola G., Alonso-Gonzalez R., Montero-Hernandez E., Cobo-Marcos M., Sanchez-Sauce B., Cuervas-Mons V., Tung-Chen Y. (2023). Usefulness of Systemic Venous Ultrasound Protocols in the Prognosis of Heart Failure Patients: Results from a Prospective Multicentric Study. J. Clin. Med..

[259] Longino A.A., Martin K.C., Leyba K.R., McCormack L., Siegel G., Sharma V.M., Riscinti M., Lopez C.O., Douglas I.S., Gill E.A. (2024). Reliability and reproducibility of the venous excess ultrasound (VExUS) score, a multi-site prospective study: Validating a novel ultrasound technique for comprehensive assessment of venous congestion. Crit. Care.

