# Supplementary material for: Assessing Venous Congestion in Acute and Chronic Heart Failure: A Review of Splanchnic, Cardiac and Pulmonary Ultrasound: Part 1: Conventional B-Mode, Colordoppler, and Vexus Protocol
Source: J Clin Med. 2025 Nov 17;14(22):8147. doi: 10.3390/jcm14228147 (PMC12653296; doi:10.3390/jcm14228147)
Supplement: Supplementary file 1 [file jcm-14-08147-s001.zip › jcm-3950694 PRISMA 2020 Compliance Statement.pdf]

PRISMA 2020 Compliance Statement

This systematic review, titled **"Assessing Venous Congestion In Acute And Chronic Heart Failure: A Review Of Splanchnic, Cardiac And Pulmonary Ultrasound: Part 1: Conventional B-Mode, Colordoppler, And Vexus Protocol,"** was conducted and reported in accordance with the **PRISMA (Preferred Reporting Items for Systematic Reviews and Meta-Analyses) 2020 guidelines**.

Specifically, this manuscript fulfills the criteria for both the PRISMA 2020 Abstract Checklist and the Main Checklist:

- **Abstract Checklist:** The provided abstract clearly outlines the **Background and Objectives**, **Materials and Methods** (including databases searched and eligibility criteria), **Results** (number of studies and participants, key findings), and **Conclusions**, as mandated by the PRISMA 2020 for Abstracts.
- **Main Checklist:** The main body of the text adheres to the comprehensive PRISMA 2020 checklist:
    - **Introduction:** The rationale and objectives are explicitly stated.
    - **Methods:** A detailed and reproducible search strategy for multiple databases is provided. The study selection process, data extraction, and data synthesis methods are thoroughly described, including the use of the PRISMA flow diagram (Table 1 in the manuscript) to document the screening process.
    - **Results:** The results of the study selection, characteristics of the included studies, and a synthesis of the findings are presented.
    - **Discussion:** A summary of the main findings, limitations, and conclusions is provided.
    - **Registration:** The manuscript transparently acknowledges the lack of a prospectively registered protocol as a limitation, which is a recommended point of disclosure under PRISMA 2020.

The completed PRISMA 2020 checklist is available as **Supplementary File S1**, ensuring full methodological transparency and reproducibility

The completed PRISMA 2020 abstract checklist is available as **Supplementary File S2**, ensuring full methodological transparency and reproducibility
