# Supplementary material for: Assessing Venous Congestion in Acute and Chronic Heart Failure: A Review of Splanchnic, Cardiac and Pulmonary Ultrasound: Part 1: Conventional B-Mode, Colordoppler, and Vexus Protocol
Source: J Clin Med. 2025 Nov 17;14(22):8147. doi: 10.3390/jcm14228147 (PMC12653296; doi:10.3390/jcm14228147)
Supplement: Supplementary file 1 [file jcm-14-08147-s001.zip › jcm-3950694 Supplementary File S1-PRISMA_2020_checklist 1.pdf]

PRISMA 2020 Checklist

Section and Topic	Item #	Checklist item	Location in Manuscript where Item is Reported
**TITLE**			
Title	1	Identify the report as a systematic review.	The title includes the word "**Review**".
**ABSTRACT**			
Abstract	2	See the PRISMA 2020 for Abstracts checklist.	The **Abstract** section (page 1) is structured per PRISMA-Abstract.
**INTRODUCTION**			
Rationale	3	Describe the rationale for the review in the context of existing knowledge.	**Introduction**, paragraphs 1-7, pages 2-4.
Objectives	4	Provide an explicit statement of the objective(s) or question(s) the review addresses.	**Introduction**, final paragraph, page 4: "This review aims to systematically examine..."
**METHODS**			
Eligibility criteria	5	Specify the inclusion and exclusion criteria for the review and how studies were grouped for the syntheses.	**Section 2.2. Study Selection**, page 5.
Information sources	6	Specify all databases, registers, websites, organisations, reference lists and other sources searched or consulted to identify studies. Specify the date when each source was last searched or consulted.	**Section 2.1. Search Strategy**, page 5: "A comprehensive and systematic search in PubMed... up to July 2025."
Search strategy	7	Present the full search strategies for all databases, registers and websites, including any filters and limits used.	**Section 2.1. Search Strategy**, page 5: "The search terms used were:..." (Note: The full strategy for all databases is not presented but is stated as available).
Selection process	8	Specify the methods used to decide whether a study met the inclusion criteria of the review, including how many reviewers screened each record and each report retrieved, whether they worked independently, and if applicable, details of automation tools used in the process.	**Section 2.2. Study Selection**, page 5 and **Section 2.3. Data Extraction**, page 6: "Reviewer disagreements... were resolved through discussion and consensus with a third independent reviewer..."
Data collection process	9	Specify the methods used to collect data from reports, including how many reviewers collected data from each report, whether they worked independently, any processes for obtaining or confirming data from study investigators, and if applicable, details of automation tools used in the process.	**Section 2.3. Data Extraction**, page 6: Data extraction process and conflict resolution are described.
Data items	10a	List and define all outcomes for which data were sought. Specify whether all results that were compatible with each outcome domain in each study were sought (e.g. for all measures, time points, analyses), and if not, the methods used to decide which results to collect.	**Section 2.2. Study Selection**, page 5: "...studies that measured physiological parameters of splanchnic circulation and related these to heart failure outcomes." The outcomes are ultrasonographic parameters.
	10b	List and define all other variables for which data were sought (e.g. participant and intervention characteristics, funding sources). Describe any assumptions made about any missing or unclear information.	**Section 2.2. Study Selection**, page 5 (population, study type). Assumptions about missing data are not explicitly stated but the qualitative synthesis approach implies handling this narratively.
Study risk of bias assessment	11	Specify the methods used to assess risk of bias in the included studies, including details of the tool(s) used, how many reviewers assessed each study and whether they worked independently, and if applicable, details of automation tools used in the process.	**Risk of Bias and Certainty Assessment**, page 6: The use of standardized tools was precluded by heterogeneity; a qualitative synthesis of limitations was performed.
Effect measures	12	Specify for each outcome the effect measure(s) (e.g. risk ratio, mean difference) used in the synthesis or presentation of results.	Not applicable. The review is a qualitative synthesis; no meta-analysis with pooled effect measures was performed.
Synthesis	13a	Describe the processes used to decide which studies were eligible for each synthesis (e.g. tabulating the study intervention	**Section 2.3. Data Extraction**, page 6

PRISMA 2020 Checklist

Section and Topic	Item #	Checklist item	Location in Manuscript where Item is Reported
methods		characteristics and comparing against the planned groups for each synthesis (item #5)).	and **Results**, page 7: The qualitative synthesis included all 148 eligible studies.
	13b	Describe any methods required to prepare the data for presentation or synthesis, such as handling of missing summary statistics, or data conversions.	Not applicable, as no quantitative synthesis was performed.
	13c	Describe any methods used to tabulate or visually display results of individual studies and syntheses.	Results are presented narratively and with figures/tables summarizing patterns (e.g., Figure 17, Table 3, Table 5).
	13d	Describe any methods used to synthesize results and provide a rationale for the choice(s). If meta-analysis was performed, describe the model(s), method(s) to identify the presence and extent of statistical heterogeneity, and software package(s) used.	**Section 2.3. Data Extraction**, page 6: "Data... were extracted and qualitatively synthesized." Rationale is provided in the **Risk of Bias** section.
	13e	Describe any methods used to explore possible causes of heterogeneity among study results (e.g. subgroup analysis, meta-regression).	Not applicable due to the qualitative synthesis approach.
	13f	Describe any sensitivity analyses conducted to assess robustness of the synthesized results.	Not applicable.
Reporting bias assessment	14	Describe any methods used to assess risk of bias due to missing results in a synthesis (arising from reporting biases).	Not performed, as stated in the **Risk of Bias** section, page 6.
Certainty assessment	15	Describe any methods used to assess certainty (or confidence) in the body of evidence for an outcome.	This was integrated into the qualitative, narrative appraisal of the evidence, as described in the **Risk of Bias and Certainty Assessment** section, page 6.
**RESULTS**			
Study selection	16a	Describe the results of the search and selection process, from the number of records identified in the search to the number of studies included in the review, ideally using a flow diagram.	**Section 2.3. Data Extraction**, page 6 and the PRISMA flow diagram (**Table 1**).
	16b	Cite studies that might appear to meet the inclusion criteria, but which were excluded, and explain why they were excluded.	The flow diagram indicates 50 full-text reports were excluded, with reasons provided in the diagram.
Study characteristics	17	Cite each included study and present its characteristics.	A full list of 148 studies is not provided in the main text, but the results synthesize findings from them. Key studies are cited throughout the **Results** section (e.g., 3.1).
Risk of bias in studies	18	Present assessments of risk of bias for each included study.	As per the methods, a formal assessment was not performed. The narrative appraisal of limitations is woven into the **Results and Discussion**, as stated on page 6.
Results of individual studies	19	For all outcomes, present, for each study: (a) summary statistics for each group (where appropriate) and (b) an effect estimate and its precision (e.g. confidence/credible interval), ideally using structured tables or plots.	Not applicable for quantitative data. For qualitative synthesis, key findings from individual studies are presented narratively in the **Results** section (e.g., Ikeda et al., Denault et al.).
Results of syntheses	20a	For each synthesis, briefly summarise the characteristics and risk of bias among contributing studies.	This is done narratively.<br>The **Results** section (page 7) begins with a summary of the included studies, and the synthesis describes consistent associations.
	20b	Present results of all statistical syntheses conducted. If meta-analysis was done, present for each the summary estimate and its precision (e.g. confidence/credible interval) and measures of statistical heterogeneity. If comparing groups, describe the direction of the effect.	Not applicable. No meta-analysis was performed.

PRISMA 2020 Checklist

Section and Topic	Item #	Checklist item	Location in Manuscript where Item is Reported
	20c	Present results of all investigations of possible causes of heterogeneity among study results.	Not applicable.
	20d	Present results of all sensitivity analyses conducted to assess the robustness of the synthesized results.	Not applicable.
Reporting biases	21	Present assessments of risk of bias due to missing results (arising from reporting biases) for each synthesis assessed.	Not performed.
Certainty of evidence	22	Present assessments of certainty (or confidence) in the body of evidence for each outcome assessed.	This is provided as a narrative summary of the evidence's strength and limitations throughout the **Discussion** section (page 32-35).
**DISCUSSION**			
Discussion	23a	Provide a general interpretation of the results in the context of other evidence.	**Discussion**, pages 32-35: The results are interpreted in the context of current guidelines and existing literature.
	23b	Discuss any limitations of the evidence included in the review.	**Discussion**, page 34-35: "several limitations must be outlined..."
	23c	Discuss any limitations of the review processes used.	**Discussion**, page 34-35: Limitations such as heterogeneity, lack of RCTs, and operator dependency are discussed.
	23d	Discuss implications of the results for practice, policy, and future research.	**Discussion**, page 35, and **Conclusion**, page 36: Implications for clinical practice and future research are detailed.
**OTHER INFORMATION**			
Registration and protocol	24a	Provide registration information for the review, including register name and registration number, or state that the review was not registered.	**Risk of Bias and Certainty Assessment**, page 6: "This review acknowledges the lack of a prospectively registered protocol as a limitation..."
	24b	Indicate where the review protocol can be accessed, or state that a protocol was not prepared.	**Risk of Bias and Certainty Assessment**, page 6: A protocol was not prepared.
	24c	Describe and explain any amendments to information provided at registration or in the protocol.	Not applicable.
Support	25	Describe sources of financial or non-financial support for the review, and the role of the funders or sponsors in the review.	**Funding** and **Acknowledgments** sections in the Back Matter.
Competing interests	26	Declare any competing interests of review authors.	**Conflicts of Interest** section in the Back Matter.
Availability of data, code and other materials	27	Report which of the following are publicly available and where they can be found: template data collection forms; data extracted from included studies; data used for all analyses; analytic code; any other materials used in the review.	**Data Availability Statement** in the Back Matter.

From: Page MJ, McKenzie JE, Bossuyt PM, Boutron I, Hoffmann TC, Mulrow CD, et al. The PRISMA 2020 statement: an updated guideline for reporting systematic reviews. BMJ 2021;372:n71. doi: 10.1136/bmj.n71. This work is licensed under CC BY 4.0. To view a copy of this license, visit https://creativecommons.org/licenses/by/4.0/
